# Genomic Epidemiology Reconstructs the Introduction and Spread of Zika Virus in Central America and Mexico

**DOI:** 10.1016/j.chom.2018.04.017

**Published:** 2018-06-13

**Authors:** Julien Thézé, Tony Li, Louis du Plessis, Jerome Bouquet, Moritz U.G. Kraemer, Sneha Somasekar, Guixia Yu, Mariateresa de Cesare, Angel Balmaseda, Guillermina Kuan, Eva Harris, Chieh-hsi Wu, M. Azim Ansari, Rory Bowden, Nuno R. Faria, Shigeo Yagi, Sharon Messenger, Trevor Brooks, Mars Stone, Evan M. Bloch, Michael Busch, José E. Muñoz-Medina, Cesar R. González-Bonilla, Steven Wolinsky, Susana López, Carlos F. Arias, David Bonsall, Charles Y. Chiu, Oliver G. Pybus

**Affiliations:** 1 Department of Zoology, University of Oxford, Oxford, UK; 2 Department of Laboratory Medicine, University of California, San Francisco, San Francisco, CA, USA; 3 UCSF-Abbott Viral Diagnostics and Discovery Center, San Francisco, CA, USA; 4 Boston Children's Hospital, Boston, MA, USA; 5 Harvard Medical School, Harvard University, Boston, MA, USA; 6 Oxford Genomics Centre, Wellcome Trust Centre for Human Genetics, University of Oxford, Oxford, UK; 7 Laboratory Nacional de Virología, Centro Nacional de Diagnóstico y Referencia, Ministerio de Salud, Managua, Nicaragua; 8 Centro de Salud Sócrates Flores Vivas, Ministerio de Salud, Managua, Nicaragua; 9 Division of Infectious Diseases and Vaccinology, School of Public Health, University of California, Berkeley, CA, USA; 10 Department of Statistics, University of Oxford, Oxford, UK; 11 Nuffield Department of Medicine, University of Oxford, Oxford, UK; 12 California Department of Public Health, Richmond, CA, USA; 13 Blood Systems Research Institute, San Francisco, CA, USA; 14 Department of Pathology, Johns Hopkins University School of Medcine, Baltimore, MD, USA; 15 División de Laboratorios de Vigilancia e Investigación Epidemiológica, Instituto Mexicano del Seguro Social, Mexico City, Mexico; 16 Division of Infectious Diseases, Feinberg School of Medicine, Northwestern University, Chicago, IL, USA; 17 Instituto de Biotecnología, Universidad Nacional Autónoma de México, Cuernavaca, Mexico; 18 Department of Medicine, Division of Infectious Diseases, University of California, San Francisco, CA, USA

**Keywords:** Zika virus, Central America, Mexico, transmission, genomics, phylodynamics, effective reproductive number, metagenomic sequencing, “spiked” primer enrichment, bait capture enrichment

## Abstract

The Zika virus (ZIKV) epidemic in the Americas established ZIKV as a major public health threat and uncovered its association with severe diseases, including microcephaly. However, genetic epidemiology in some at-risk regions, particularly Central America and Mexico, remains limited. We report 61 ZIKV genomes from this region, generated using metagenomic sequencing with ZIKV-specific enrichment, and combine phylogenetic, epidemiological, and environmental data to reconstruct ZIKV transmission. These analyses revealed multiple independent ZIKV introductions to Central America and Mexico. One introduction, likely from Brazil via Honduras, led to most infections and the undetected spread of ZIKV through the region from late 2014. Multiple lines of evidence indicate biannual peaks of ZIKV transmission in the region, likely driven by varying local environmental conditions for mosquito vectors and herd immunity. The spatial and temporal heterogeneity of ZIKV transmission in Central America and Mexico challenges arbovirus surveillance and disease control measures.

## Highlights

• Zika virus was introduced from Brazil to Central America via Honduras in summer 2014
• Undetected spread to other Central American countries and Mexico occurred from late 2014
• Genetic and epidemiological data indicate biannual peaks of transmission
• Local variation in vector abundance could explain the biannual trend of transmission

## Graphical Abstract

**Figure:**
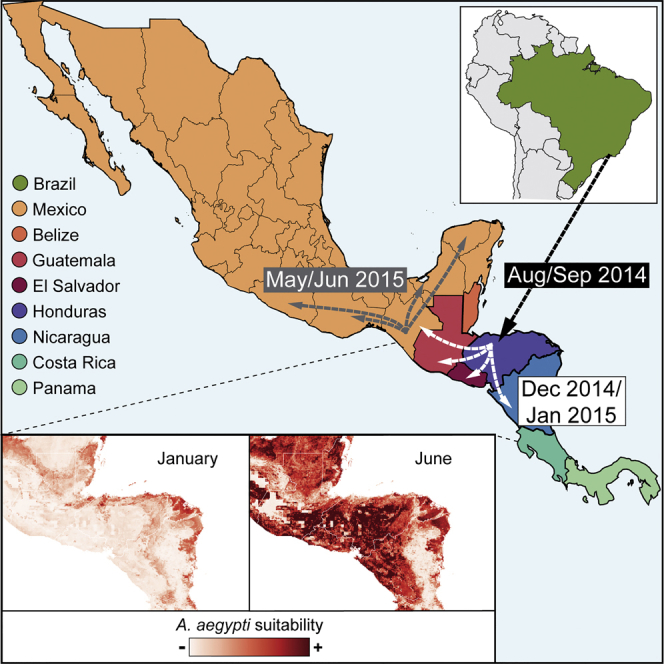

## Teaser

Thézé et al. examine the genomic epidemiology of Zika virus in Central America and Mexico. Following its likely introduction to Honduras in 2014, the virus spread undetected in the region. Genetic and epidemiological data indicate that biannual transmission peaks occurred, and could potentially be explained by local variation in mosquito abundance.

## Introduction

Zika virus (ZIKV), first discovered in 1947 in a Ugandan macaque, is an RNA virus of the *Flavivirus* genus. Vector-borne transmission of ZIKV occurs primarily from the bite of *Aedes* sp. mosquitoes, although transmission has also been described via blood transfusion, sexual contact, and from mother to child (Musso and Gubler, 2016). Until comparatively recently, reports of ZIKV infection in humans were limited to small outbreaks, resulting in relatively mild, self-limited disease known as Zika fever, whose symptoms include maculopapular rash, headache, conjunctivitis, and myalgia (Musso and Gubler, 2016).

In May 2015, ZIKV cases were reported in the Americas for the first time, in Brazil. ZIKV was subsequently reported in many countries of South America (October 2015), Central America (November 2015), and the Caribbean (December 2015) (Kindhauser et al., 2016). To date, 47 of 55 countries and territories in the Americas have confirmed autochthonous ZIKV transmission (WHO situation report March 10, 2017). By the end of the epidemic, it is estimated that ZIKV will have infected ∼100 million people in the Americas (Perkins et al., 2016). The emergence of ZIKV in the Americas also revealed a link between ZIKV infection during pregnancy and fetal congenital malformations, including severe microcephaly (Cauchemez et al., 2016, Mlakar et al., 2016), an association now considered proven by the weight of available evidence (Rasmussen et al., 2016). ZIKV infection has also been associated with severe neurological and autoimmune complications, such as encephalitis and Guillain-Barré syndrome (Cao-Lormeau et al., 2016, Parra et al., 2016). At least 24 countries in the Americas have reported cases of ZIKV-associated birth defects and 15 have reported ZIKV-linked neurological syndromes (WHO situation report March 10, 2017).

Early genetic studies of the American ZIKV epidemic showed that it arose from a single Asian genotype lineage that was introduced to the Americas sometime between late 2013 and early 2014 (Faria et al., 2016), and may have been imported from French Polynesia (Cao-Lormeau et al., 2014). Phylogenetic analyses indicate that ZIKV was present in northeast Brazil by mid-2014, suggesting that ZIKV circulated and expanded its geographic range in the Americas for at least a year prior to its detection (Faria et al., 2017, Metsky et al., 2017, Naccache et al., 2016). These genetic studies have clarified the timescale of the establishment and spread of ZIKV in Brazil, the Caribbean, and the United States (Grubaugh et al., 2017).

Our current understanding of the genomic epidemiology of ZIKV in the Americas remains limited, in part due to the difficulties in recovering ZIKV genomes directly from clinical samples (Faria et al., 2017, Lanciotti et al., 2016, Metsky et al., 2017, Quick et al., 2017). Despite millions of potential infections, only ∼400 full or partial (>1,500 nt) ZIKV genomes from the Americas have been reported. Genetic data from key affected locations remain scarce. Comparatively little is known about ZIKV genetic diversity in Central America and Mexico, where transmission was first reported in late 2015 (Kindhauser et al., 2016) and where the estimated climatic suitability for *Aedes* sp. vectors is high (Kraemer et al., 2015). Central America and Mexico have been predicted to be at high risk for ZIKV epidemics (Messina et al., 2016) and for infections among childbearing women (Perkins et al., 2016).

In this study, we investigate the genetic diversity and transmission history of ZIKV in Central America and Mexico (hereafter referred to as CAM). We report 61 complete and partial ZIKV genome sequences, representing infections from returning travelers to the United States and autochthonous infections of residents of Mexico, Nicaragua, Honduras, Guatemala, and El Salvador. Using a combination of phylogenetic, epidemiological, and environmental data, we reveal the timing of the introduction and the spread of ZIKV in CAM and uncover the spatial and temporal heterogeneity of ZIKV transmission in the region.

## Results

### Sample Collection and qRT-PCR Testing

**Figure 1:**
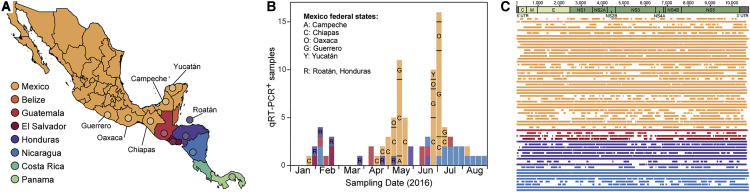
Zika Virus Sampling and Sequencing in Central America and Mexico (A) Map of Central America and Mexico. Colored circles indicate the sampling locations of Zika virus sequences generated in this study, and the locations of publicly available sequences from Central America and Mexico. (B) The temporal and geographic distribution of Zika virus qRT-PCR-positive samples tested in this study. Samples are colored according to their sampling location. (C) Consensus genome coverage of the Zika virus sequences generated in this study. Sequences are colored according to their sampling location and the Zika virus genome structure is shown above the plot.

Serum and urine samples obtained from patients living in, or who had traveled to, CAM and who exhibited symptoms consistent with ZIKV infection (Table S1) were screened for ZIKV by real-time qRT-PCR. A total of 95 specimens, sampled between January and August 2016, were qRT-PCR positive (59 from Mexico, 16 from Nicaragua, 9 from Honduras, 8 from Guatemala, 3 from El Salvador; Figures 1A and 1B; Table S1). For 52 Mexico samples, the federal states where samples were collected were known (Campeche, Chiapas, Guerrero, Oaxaca, and Yucatán). Positive samples were collected, on average, 2 days after symptom onset (Table S1), consistent with previous ZIKV studies in Brazil (Faria et al., 2017) and Colombia (Pacheco et al., 2016). This period likely reflects the narrow 3-day overlap between ZIKV viremia (which persists for ∼9 days after infection) and the onset of symptoms (at ∼6 days after infection) (Lessler et al., 2016). The median cycle threshold (Ct) value of qRT-PCR-positive samples was 36, similar to previous studies (Faria et al., 2017), and corresponded to a low RNA titer approaching the detection threshold for PCR (Table S1).

### Zika Virus Genome Sequencing

An approach that combined metagenomic next-generation sequencing (mNGS) (Luk et al., 2015) with a newly developed “spiked” primer enrichment strategy (see STAR Methods) was applied to 81 of the 95 qRT-PCR-positive samples. This strategy successfully identified mNGS reads that matched ZIKV in 71 of those 81 samples. Coverage of the consensus ZIKV genomes generated from each sample ranged from 2% to 100%, with an average of 64% (Table S1). Further bait capture probe enrichment for ZIKV genome recovery was attempted on 10 samples, whose original genome coverages ranged from 9% to 73%. Bait capture probe enrichment expanded coverage for all cases but one, with an average gain of 10.3% (0.0%–22.3%) coverage (Table S1).

The remaining 14 ZIKV samples, all from Nicaragua (Table S1), were processed in a separate laboratory using an alternative mNGS method that employed bait probe capture of metagenomic libraries without the use of spiked primers (Bonsall et al., 2015). Coverage of the consensus ZIKV genomes generated from the Nicaraguan samples ranged from 1% to 100%, with an average of 47% (Table S1).

Many sequenced samples had low genome coverage (Table S1). Coverage was variable for samples with Ct values >30 (Table S1), and missing regions appeared to be randomly distributed across the ZIKV genome (Figure 1C). We undertook a preliminary phylogenetic analysis to explore the trade-off between minimum genome coverage and the number of sequences included in the alignment (Figure S1). Specifically, we measured ZIKV phylogenetic accuracy on pseudoreplicate alignments in which the number and incompleteness of genomes was varied. A notable decrease in phylogenetic accuracy was observed when partial genome coverage was reduced from 40% to 20% (Figure S1). Following this, we chose to retain only those ZIKV sequences with >30% genome coverage. Other analyses confirmed that our dataset contained sufficient phylogenetic and temporal information for further analysis (see Figure S2). This resulted in a final dataset of 61 sequences with an average genome coverage of 82.6% (Figure 1C and Table S1).

### Phylogenetic Analyses

The sequence alignment (Data S1) used for phylogenetic analyses comprised the 61 ZIKV sequences generated here, plus 298 published and available sequences, as of June 2017. We first estimated a maximum-likelihood (ML) phylogeny with bootstrap node support values (Figure S2). This tree revealed that 102 of the 107 ZIKV sequences from CAM fell into a single monophyletic clade (clade B in Figure S2; bootstrap score = 65%), which also contained two sequences from the United States (see Grubaugh et al., 2017, Metsky et al., 2017). This CAM clade was most closely related to ZIKV sequences from Brazil (clade A in Figure S2). Four ZIKV sequences from Panama and one from Mexico did not fall within clade B and were instead placed within a different clade (clade C in Figure S2; bootstrap score = 85%). Within clade C, Panama sequences were most closely related to those from Colombia, whereas the Mexico sequence groups were related to strains from Martinique. Thus, ZIKV had been introduced to CAM from other locations on multiple occasions, but most CAM infections descended from just one importation event (clade B).

**Figure 2:**
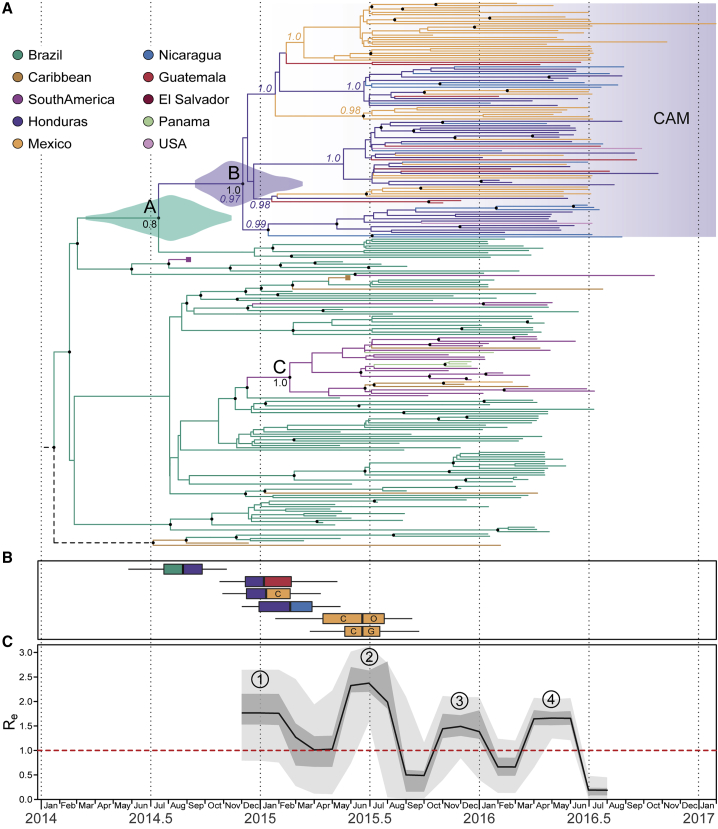
Genomic Epidemiology of Zika Virus in Central America and Mexico (A) A maximum clade credibility phylogeny estimated from complete and partial Zika virus sequences from the Americas (see STAR Methods for details). For visual clarity, basal Asian and Pacific lineages are not displayed, and two large clades (corresponding to groups of sequences in South America and the Caribbean) have been collapsed and their positions indicated by purple and brown squares, respectively. Violin plots show the posterior distributions of the estimated dates of nodes A and B (see main text). Branch colors indicate the most probable ancestral lineage locations of isolates from the Central America and Mexico region. Circles at internal nodes denote clade posterior probabilities >0.75. For selected nodes, colored numbers show the posterior probabilities of inferred ancestral locations, while black numbers are the clade posterior probabilities. (B) Earliest inferred dates of Zika virus introduction to and within Central America and Mexico. Each box-and-whisker plot corresponds to the earliest movement between a pair of locations with well-supported virus lineage migration (left color, source location; right color, destination location). Letters indicate federal states of Mexico (C, Chiapas; O, Oaxaca; G, Guerrero). (C) Effective reproductive number (R_e_) through time, estimated using a birth-death skyline approach. The black line, darker shading, and lighter shading represent, respectively, the median posterior estimate of R_e_, and its 50% and 95% highest posterior density credible intervals. Circled numbers indicate the four periods of epidemic dynamics mentioned in the main text.

A regression of genetic divergence against sampling time confirmed that the dataset was suitable for molecular clock analysis (Figure S2; R^2^ = 0.65). To reconstruct the dissemination of ZIKV within CAM, we used a well-established Bayesian molecular clock phylogeographic approach (Lemey et al., 2009). The resulting maximum clade credibility tree was largely consistent with previous studies (Figure 2A; see also Figure S3) (Faria et al., 2017, Faria et al., 2016, Grubaugh et al., 2017, Metsky et al., 2017, Naccache et al., 2016) and with the ML phylogeny (Figure S2). As before, most sequences from CAM were placed in a single clade (clade B in Figure 2A; posterior probability = 1.0). We estimated the date of the most recent common ancestor (MRCA) of clade B to be December 2014 (Figure 2A; 95% highest posterior density [HPD] = September 2014 to March 2015), diverging from Brazilian strains around July 2014 (node A in Figure 2A; 95% HPD = March 2014 to November 2014; posterior probability = 0.8). Hence we estimated that the clade B lineage was exported from Brazil to Central America between July and December 2014. This timescale was approximately three months earlier than that estimated in previous studies (Faria et al., 2017, Metsky et al., 2017), a refinement likely due to the larger number of strains from CAM included in this analysis. Four ZIKV strains from Panama and Mexico did not result from the clade B introduction and were instead likely introduced from Colombia or the Caribbean during the second half of 2015 (clade C; Figure 2A).

We used a discrete trait analysis to infer the ancestral location of each phylogeny branch (Lemey et al., 2009). This indicated that the most likely location of the common ancestor of clade B was Honduras (Figure 2A; posterior probability = 0.97). This result was unlikely to be an artifact of sampling intensity because clade B contained more sequences from Mexico (n = 47) than from Honduras (n = 31) and because random subsampling of the dataset confirmed that Honduras as the ancestral node location was the most likely scenario (Figure S4). Despite being smaller and less populous than Mexico, Honduras accounted for >50% of all suspected ZIKV cases in the CAM region (WHO, 2017). Our phylogeographic analysis estimated that ZIKV was introduced to Honduras from Brazil around July to September 2014 (Figure 2B), and that subsequent dissemination of ZIKV to Guatemala and Nicaragua and to southern Mexico likely occurred in late 2014 to early 2015 (December 2014 to February/March 2015). The state-level sampling of viruses from Mexico indicated that ZIKV was most likely first introduced into Mexico (from Honduras) via the southern state of Chiapas. Our reconstruction suggested that ZIKV subsequently spread within Mexico, from Chiapas to Oaxaca and Guerrero states, and that this within-country movement occurred in mid-2015 (April/May to July 2015) (Figure 2B).

### Genetic Estimates of Epidemic Dynamics

We used the Bayesian birth-death skyline model (Stadler et al., 2013) to estimate temporal changes in R_e_, the effective reproductive number of the CAM clade of ZIKV, directly from virus sequence data (Figure 2C). For each point in time, R_e_ represents the average number of secondary infections caused by a case (hence R_e_ > 1 and R_e_ < 1 represents epidemic growth and decline, respectively). We observed four periods of epidemic growth (estimated R_e_ > 1; red dotted line in Figure 2C) within 2015 and 2016, although only the second and fourth periods were statistically significant, with a ≥95% posterior probability that R_e_ > 1. The first period coincided with ZIKV spread from Honduras to other CAM countries. The second growth period, mid-2015, reached a median R_e_ > 2 and coincided with the within-country movement in Mexico (Figure 2C). This second period also corresponded to a rapid radiation of ZIKV lineages in clade B (Figure 2A) and preceded the first reported cases of ZIKV in CAM. The third period occurred immediately prior to the rapid increase in reported ZIKV cases in CAM in early 2016 (Ikejezie et al., 2017). The fourth growth period corresponded to the epidemic observed during April–July 2016 in CAM (Ikejezie et al., 2017).

### Epidemiological Data and Environmental Suitability

**Figure 3:**
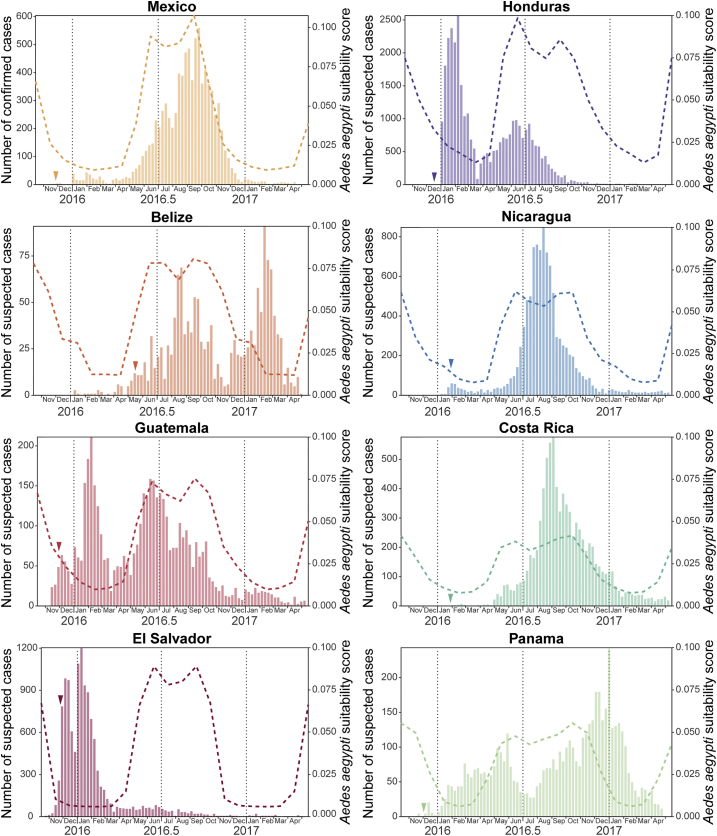
Geographic and Temporal Distribution of Zika Virus Cases in Central America and Mexico Each panel corresponds to a country within the Central America and Mexico region. In each panel, the bar plots show notified Zika virus cases per week until May 2017 (plots adapted from PAHO); dashed lines indicate the estimated climatic vector suitability score, averaged across the country (see STAR Methods for details); and a small colored arrowhead indicates the date of earliest confirmation of autochthonous Zika virus cases in that country.

To place the above genomic findings in their epidemiological context, we analyzed available national-level epidemiological data for countries in the CAM region. Weekly suspected ZIKV cases from Central American countries and confirmed cases for Mexico from 2015 to 2017 were extracted from the Pan American Health Organization (PAHO) epidemiological reports (June 2017; Figure 3). The date of first detection of ZIKV in each country ranged from November 2015 in El Salvador to May 2016 in Belize. Countries reported a variety of epidemic trajectories; Costa Rica, Mexico, and Nicaragua exhibited one epidemic peak in late summer 2016, while two peaks in transmission (winter and summer) were observed in Belize, Honduras, and Guatemala. (Note that here we use Northern Hemisphere definitions of summer and winter, which correspond approximately to wet and dry seasons in tropical regions north of the equator.) Suspected ZIKV cases in El Salvador peaked only once, at the beginning of January 2016, while those in Panama showed no clear temporal pattern during 2016. These data should be interpreted cautiously because (1) case reporting varies among countries, (2) syndromic surveillance may not be able to distinguish between ZIKV and other infections with similar symptoms, and (3) reporting intensity may vary through time, e.g., during national holidays.

To better understand these temporal patterns, we computed, for each country, a measure of environmental suitability for the vector *Aedes aegypti* through time. The score was derived from monthly temperature, relative humidity, and precipitation data, as previously described (Bogoch et al., 2016, Kraemer et al., 2015). We observed high climatic suitability scores between May and October for most Central American countries (Belize, Guatemala, Honduras, El Salvador, Nicaragua) and Mexico. Honduras was found to have the highest average suitability score (Figure 3). Vector suitability scores in Costa Rica and Panama were typically lower and exhibited less seasonal variation. The scores in Figure 3 represent average suitability across each country, except for Mexico, for which the suitability score represents only those 11 federal states that correspond to 95% of confirmed ZIKV cases (Chiapas, Colima, Guerrero, Hidalgo, Morelos, Nuevo León, Oaxaca, Quintana Roo, Tabasco, Veracruz, and Yucatán).

We observed a strong association between estimated vector suitability and weekly suspected ZIKV cases for Mexico, Nicaragua, and Costa Rica (R^2^ > 0.5; p < 0.001; Figure 3 and Table S2), a trend previously reported in different Brazilian regions (Faria et al., 2017). However, Belize, El Salvador, Guatemala, Honduras, and Panama did not show any such association (R^2^ < 0.3; p > 0.01; Figure 3 and Table S2). Suspected cases peaked twice in Belize, Guatemala, and Honduras, once between May and October (corresponding to the annual peak of mosquito suitability) and once between November and March. Unexpectedly, this latter rise in cases corresponded to a period of low predicted vector suitability, and a similar winter peak was also observed in El Salvador.

### Spatial Heterogeneity of Vector Suitability

**Figure 4:**
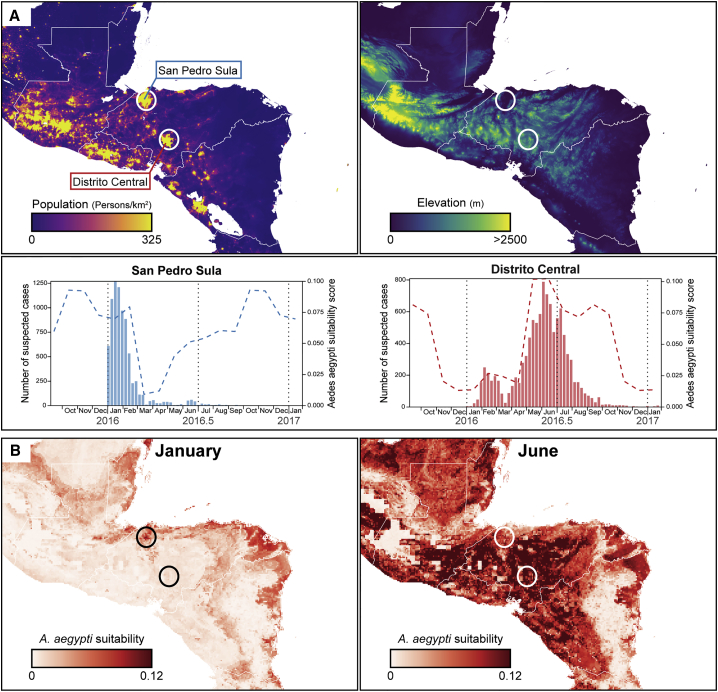
Spatial and Temporal Heterogeneity of Zika Virus Transmission in Honduras (A) Maps of Central America centered on Honduras showing population density (left panel) and elevation (right panel). In the bottom panel, the bar plots show notified Zika virus cases per week for the two main cities of Honduras highlighted on the population density map. For each bar plot, dashed lines indicate the estimated climatic vector suitability score for the two cities. (B) Maps of estimated *Aedes aegypti* climatic suitability scores. Monthly averages for January and June are shown.

The viral genetic (Figure 2) and epidemiological (Figure 3) results presented above both indicated the presence of two ZIKV epidemics in the CAM region, most notably in Honduras, Guatemala, and Belize (Figure 3). To explore whether this unexpected pattern was due to spatial heterogeneity, we sought city-wide regional data on ZIKV incidence. Such information was available for Honduras, which accounted for >50% of reported ZIKV cases in CAM. Specifically, total numbers of suspected ZIKV cases were available for the two main cities of Honduras (Distrito Central, 1.19 million inhabitants, and San Pedro Sula, 1.07 million inhabitants; data from 2016 Honduran Ministry of Health situation reports). The case numbers are shown in Figure 4A together with estimated mosquito suitability scores specific to each city. Remarkably, the two peaks in ZIKV cases observed at the national level (Figure 3) corresponded to distinct, single epidemics in each city; cases in San Pedro Sula are almost exclusively in winter while those reported in Distrito Central were overwhelmingly during the summer (Figure 4A). The mosquito suitability scores for the two main cities were asynchronous, with suitability in San Pedro Sula peaking between November and February, and in Distrito Central between May and October (Figure 4A). For Honduras, reported ZIKV cases and mosquito suitability scores were much more strongly associated at the local than at the national level (Figures 3 and 4A). Notably, the ZIKV epidemic in San Pedro Sula ended abruptly in March as predicted suitability rapidly declined. The difference in the vector suitability scores between these two cities likely results from their distinct geographies, as Distrito Central is situated in the central highlands of Honduras and San Pedro Sula in the Atlantic lowlands (Figure 4B), the average precipitation in winter being much higher in the latter than in the former (see Discussion).

## Discussion

A tropical climate makes many locations in CAM susceptible to mosquito-borne diseases. Dengue virus (DENV) has caused outbreaks in the region regularly for several decades (Brathwaite Dick et al., 2012) and chikungunya virus (CHIKV) was introduced in 2014 and subsequently spread throughout the region (Sahadeo et al., 2017). Consequently, the CAM region was predicted to be at high risk for ZIKV transmission (Messina et al., 2016) with a concomitant risk of cases of severe ZIKV-associated disease (Perkins et al., 2016). The first ZIKV cases in CAM were reported in November 2015 (Kindhauser et al., 2016), about 6 months later than the date of introduction estimated by previous studies (Faria et al., 2017, Metsky et al., 2017). Analysis of the genomes reported in this study provides a more detailed understanding of the introduction and progression of ZIKV in CAM.

Viral genome sequence recovery from low-titer clinical samples is a substantial technical challenge for viral genomic epidemiology. ZIKV is difficult to sequence given the brief period of detectable viremia (Waggoner and Pinsky, 2016) and lower overall viral titers in returning travelers, for whom medical care is often delayed. Here we used metagenomic sequencing that employed “spiked” primers and/or oligonucleotide capture probes to sufficiently enrich low-titer samples for genome recovery. The coverage achieved across 61 assembled ZIKV genomes was sufficient for robust molecular evolutionary analysis, with missing sequence regions approximately randomly distributed across the genome. Other viruses or panels of viruses can be specifically targeted using both of these approaches (Bonsall et al., 2015, Briese et al., 2015, Koehler et al., 2014, Wylie et al., 2015). The spiked primer strategy retains the capability to detect co-infections metagenomically (Sardi et al., 2016), which may be particularly useful for arboviruses, because DENV and CHIKV share mosquito vectors with ZIKV, co-circulate in many populations, and exhibit overlapping sets of clinical symptoms.

Our phylogenetic analyses show that ZIKV was introduced into CAM multiple times, but only one such introduction has become epidemiologically dominant and spread between countries in the region. We infer that this lineage (clade B; Figures 2A, S1, and S2) originated from Brazil, where ZIKV transmission is thought to have been established since early 2014 (Faria et al., 2017, Grubaugh et al., 2017, Metsky et al., 2017). This main introduction into CAM likely occurred in Honduras in late summer 2014, when the country had a notably high overall predicted environmental suitability for the ZIKV mosquito vector (Figures 2B and 3). Thus ZIKV circulated in CAM for at least a year before being first detected there in November 2015, corroborating previous reports of undetected ZIKV spread in this and other regions in the Americas (Faria et al., 2017, Grubaugh et al., 2017, Metsky et al., 2017).

Our analysis suggests that ZIKV firstly spread from Honduras to other countries in CAM (notably Guatemala, southern Mexico, and Nicaragua) between late 2014 and early 2015 (Figures 2A and 2B), when mosquito suitability in the Atlantic lowlands of Honduras was highest (Figures 4A and 4B). Alternatively, if current data underestimate the genetic diversity of ZIKV in Honduras, dissemination events from Honduras may have been more numerous and more recent (i.e., during summer 2015). However, the former scenario is more consistent with the early reported presence of ZIKV in southeastern states of Mexico in January–March 2015 (Díaz-Quiñonez et al., 2016) and a subsequent dissemination of ZIKV within Mexico during summer 2015 (Figures 2A and 2B).

Multiple lines of evidence point to complex annual trends in ZIKV transmission in CAM, contrasting with a single transmission season observed elsewhere (Faria et al., 2017). Firstly, the time series of qRT-PCR-positive samples exhibits two waves within 2016; a larger wave in spring and summer dominated by samples from Mexico and Nicaragua, and a smaller winter wave comprising samples from Honduras, Guatemala, El Salvador, Nicaragua, and elsewhere (Figures 1A and 1B). Secondly, ZIKV lineage movements among countries also occur in both winter and summer (Figure 2B). Thirdly, genetic analysis of the effective reproductive number of ZIKV clade B (Figure 2C) reveals periods of epidemic growth approximately every 6 months rather than once a year. Lastly, reported suspected ZIKV cases in 2016 and 2017 peak twice a year in Belize, Honduras, and Guatemala (Figure 3). Although each of these observations carries substantial uncertainty, their convergence is noteworthy.

The reasons for this biannual trend are unknown and a number of hypotheses can be put forward: (1) it is possible that ZIKV cases were over-reported in some locations in winter 2016, perhaps due to heightened awareness immediately following the first suggestions of a link between ZIKV and microcephaly; (2) ZIKV introduction into a wholly susceptible population might generate substantial transmission even when vector abundance is comparatively low—if true, however, this implies that little herd immunity accumulated in the CAM region during 2014 and 2015; (3) the contribution of latent infection and sexual transmission (Gao et al., 2016, Maxian et al., 2017, Towers et al., 2016) to ZIKV incidence is currently not well characterized, and requires further investigation, and could in theory contribute to reported cases in winter (Allard et al., 2017); (4) variation in vector abundance at the regional or city-wide level may hide local environmental heterogeneity.

In this study we were able to investigate the fourth hypothesis and found evidence that, in Honduras at least, the two epidemic peaks correspond to outbreaks in distinct locations of the country at different times. If this hypothesis is correct then spatiotemporal variation in vector abundance should also affect the transmission of other viruses transmitted by *Aedes* sp. (Perkins et al., 2013). In Honduras there is evidence for sustained transmission of CHIKV in January–March 2015 in addition to an epidemic later in the year (Zambrano et al., 2017). Furthermore, a previous study (Brooks et al., 2017) reported only a winter peak in ZIKV cases on Roatán island, Honduran Bay, whose climate likely differs from the Honduran highlands. We note that the hypotheses outlined above are not mutually exclusive. Indeed, herd immunity might explain why a second ZIKV epidemic was not observed in San Pedro Sula, Honduras, even though vector suitability there rose again in summer (Figure 4A).

Annual variation in mosquito suitability, which is strongly associated with precipitation in the tropics (Kraemer et al., 2015), varies greatly not only within Honduras but across the CAM region as a whole. In general, during winter there is high vector suitability in the Atlantic lowlands and low suitability in central/Pacific highlands, while in summer months there is typically high or medium suitability in most regions (Figure 4B). The epidemiological impact of this annual variation may be particularly pronounced in Honduras because its Atlantic lowlands are highly populated (Figure 4A). Further investigation and testing of these ideas will require both genetic and epidemiological data at local as well as national scales, and for multiple countries in the CAM region.

The spatial and temporal heterogeneity of ZIKV in CAM contributes to the challenges faced by arbovirus control programs in the region. New surveillance methods, such as real-time epidemic sequencing or large-scale active mosquito trapping, combined with the broad-based metagenomics approaches reported in this study, could in theory provide timely information to inform epidemic response and control interventions. Further research is needed to understand subtle temporal fluctuations in vector abundance and herd immunity in different locations, particularly in CAM (Lourenço et al., 2017).

## STAR★Methods

### Key Resources Table

**Table:** 

REAGENT or RESOURCE	SOURCE	IDENTIFIER
**Bacterial and Virus Strains**		

Zika virus strains from Central America and Mexico	This study	N/A

**Biological Samples**		

Serum and urine samples obtained from patients living in, or who had travelled to, Central America or Mexico and who exhibited symptoms consistent with Zika virus infection (see Table S1)	- Health Center Sócrates Flores Vivas, Managua, Nicaragua - California Department of Public Health, Richmond, CA, USA - Blood System Research Institue, San Francisco, CA, USA - Central Laboratory of Epidemiology, Mexico City, Mexico	N/A

**Critical Commercial Assays**		

QIAamp Viral RNA Mini Kit	Qiagen	Cat # 204443
QuantiTect Probe RT-PCR Kit	Qiagen	Cat # 204445
EZ1 Virus Mini Kit v2.0	Qiagen	Cat # 955134
SuperScript III Reverse Transcription Kit	Invitrogen	Cat # 18080085
Turbo DNase kit	Thermo-Fisher Scientific	Cat # AM2238
Baseline-ZERO DNase	Epicentre	Cat # DB0715K
SuperScript III Platinum One-Step qRT-PCR kit	Invitrogen	Cat # 11745500
Sequenase version 2.0 DNA Polymerase	Life Technologies	Cat # 70775Y200UN
DNA Clean & Concentrator kit	Zymo Research	Cat # D4014
RNA Clean & Concentrator kit	Zymo Research	Cat # R1016
RNA 6000 Pico kit	Agilent	Cat # 5067-1513
Nextera XT DNA Library Preparation Kit	Illumina	Cat # FC-131-1024
NEB Ultra Directional library kit	New England Biolabs	Cat # E7420S
SeqCap EZ Hybridization and Wash Kit	Roche	Cat # 05634261001
SeqCap EZ Accessory kit	Roche	Cat # 07145594001
xGen Universal Blockers - TS	Integrated DNA Technologies	Cat # 1075474
xGen Lockdown Probes	Integrated DNA Technologies	N/A
Dynabeads M-270 Streptavidin	Invitrogen	Cat # 65305
High Sensitivity D1000 ScreenTape	Agilent	Cat # 5067- 5584
Agencourt AMPure XP	Beckman Coulter	Cat # A63880
Qubit dsDNA HS Assay Kit	Qiagen	Cat # Q32851
ZCD assay	Waggoner et al., 2016	N/A
CDC Trioplex assay	Santiago et al., 2018	N/A
CDC monoplex assay	Lanciotti et al., 2016	N/A

**Deposited Data**		

61 Zika virus sequences from Central America and Mexico	This study	National Center for Biotechnology Information (NCBI) Genbank: KY606271-KY606274, MF434516-MF434522 and MF801377-MF801426
298 publicly available Zika virus sequences	N/A	National Center for Biotechnology Information (NCBI) Genbank: EU545988, JN860885, KJ776791, KU312312-KU312315, KU321639, KU365777-KU365780, KU497555, KU501215-KU501217, KU509998, KU527068, KU646827, KU646828, KU647676, KU681081, KU681082, KU707826, KU729217, KU729218, KU740184, KU758868-KU758877, KU761564, KU820897, KU820898, KU853012, KU870645, KU922960, KU926309, KU926310, KU937936, KU940224, KU940227, KU940228, KU955590, KU991811, KX051563, KX056898, KX087101, KX087102, KX101060-KX101067, KX156774-KX156776, KX197192, KX197205, KX198135, KX212103, KX247646, KX262887, KX269878, KX280026, KX369547, KX377337, KX421195, KX446950, KX446951, KX447509-KX447521, KX520666, KX548902, KX601168, KX673530, KX702400, KX766028, KX766029, KX811222, KX830930, KX856011, KX879603, KX879604, KY003153-KY003157, KY014295-KY014329, KY120349, KY272991, KY317936-KY317940, KY325465, KY328289, KY348640, KY558989-KY559032, KY631492, KY631493, KY693676-KY693680, KY765317, KY765318, KY765323-KY765325, KY785409-KY785485, KY817930, KY927808, KY989971, MF098764-MF098771

**Oligonucleotides**		

ZIKV 1086 5'-CCGCTGCCCAACACAAG-3'	Lanciotti et al., 2016	N/A
ZIKV 1162c 5'-CCACTAACGTTCTTTTGCAGACAT-3'	Lanciotti et al., 2016	N/A
ZIKV 1107-FAM 5'-AGCCTACCTTGACAAGCAGTCA GACACTCAA-3'	Lanciotti et al., 2016	N/A
ZIKV Forward 5'-CAGCTGGCATCATGAAGAAYC-3'	Waggoner et al., 2016	N/A
ZIKV Reverse 1 5'-CACTTGTCCCATCTTCTTCTCC-3'	Waggoner et al., 2016	N/A
ZIKV Reverse 2 5'-CACCTGTCCCATCTTTTTCTCC-3'	Waggoner et al., 2016	N/A
ZIKV Probe 5'-CYGTTGTGGATGGAATAGTGG-3'	Waggoner et al., 2016	N/A
13-mer spiked primers for Zika virus (see Table S3)	This study	N/A
Bait capture probes for Zika virus (see Table S4)	This study	N/A

**Software and Algorithms**		

BEAST	Drummond et al., 2012	http://beast.community
BEAST2	Bouckaert et al., 2014	https://www.beast2.org/
BLAST	Altschul et al., 1997	https://blast.ncbi.nlm.nih.gov/Blast.cgi
bwa	Li and Durbin, 2009, Li, 2013	http://bio-bwa.sourceforge.net/
CD-HIT	Li and Godzik, 2006	http://weizhongli-lab.org/cd-hit/
GATK	McKenna et al., 2010	https://software.broadinstitute.org/gatk/
jModelTest2	Darriba et al., 2012	https://github.com/ddarriba/jmodeltest2
MAFFT	Katoh and Standley, 2013	https://mafft.cbrc.jp/alignment/server/
PhyML	Guindon and Gascuel, 2003	http://www.atgc-montpellier.fr/phyml/
Primer3	Untergasser et al., 2012	https://primer3plus.com
QGIS	QGIS Development Team	https://qgis.org
R Statistical Computing Software	The R Foundation	https://www.r-project.org/
R-package bdskytools	N/A	https://github.com/laduplessis/bdskytools
R-package ggplot2	Wickham, 2016	http://ggplot2.org/
R-package ggtree	Yu et al., 2016	https://github.com/GuangchuangYu/ggtree
SURPI	Naccache et al., 2014	https://github.com/chiulab/surpi
TempEst	Rambaut et al., 2016	http://beast.community/tempest
Tracer	Rambaut et al., 2018	http://beast.community/tracer

**Other**		

Alignment used in phylogenetic analyses, including 298 publicly available Zika virus sequences and 61 Zika virus sequences generated in this study (see Data S1)	This study	N/A

### Contact for Reagent and Resource Sharing

Further information and requests for laboratory resources and reagents should be directed to and will be fulfilled by the corresponding author, Charles Y. Chiu (Charles.Chiu@ucsf.edu). Requests for computional resources and files should be directed to and will be fulfilled by the corresponding author, Oliver G. Pybus (oliver.pybus@zoo.ox.ac.uk).

### Experimental Model and Subject Details

#### Sample Collection

##### Mexico Samples

From December 2015, serum samples from all suspected Zika virus (ZIKV) cases detected through passive surveillance from the 35 Mexican Social Security Institute (IMSS) delegations nationwide (located in 32 Mexican states) were submitted for ZIKV diagnosis to the Central Laboratory of Epidemiology (CLE), IMSS in Mexico City. All cases met the following suspect case definition: a person of any age who present exanthema accompanied by two or more of the following symptoms: fever, headache, conjunctivitis, arthralgia, myalgia, edema, pruritus and retroocular pain plus living in or having travelled to, within two weeks of fever onset, an area endemic for *Aedes aegypti* or *A*. *albopictus* with confirmed cases within the locality. Using red cap tubes (without anticoagulant), 5 mL of peripheral blood were taken by venipuncture of the inside part of the elbow, from which 2 to 3 mL of serum were obtained and sent under refrigeration conditions (2-8°C) to the Central Laboratory of Epidemiology of IMSS in compliance with International Air Transport Association (IATA) triple packaging standards. All samples were taken during the acute phase of the disease (0-5 days following symptom onset).

ZIKV samples from Mexico were collected as part of the national epidemiological surveillance program of the Mexican Institute of Social Security, which is a branch of the Ministry of Health. Samples along with accompanying clinical and epidemiological data were de-identified prior to analysis, and are thus considered exempt from human subject regulations with waiver of informed consent according to 45 CFR 46.101(b) of the United States Department of Health and Human Services.

##### Returning Travelers and Honduras Samples

From December 2015, serum and urine samples were obtained and provided by the California Department of Public Health (CDPH) from 31 returning travelers from Mexico and the Central American Isthmus (El Salvador, Guatemala and Honduras). An additional 6 samples from patients in Roatán, Honduras were provided by the Blood Systems Research Institute (BSRI). These samples were extracted from patients matching the above suspect case definition.

ZIKV samples from the CDPH were de-identified prior to analysis and are considered exempt from human subject regulations. The 6 samples from Honduras were collected under protocols approved by the institutional review boards of the University of California, San Francisco, and Universidad Nacional Autonoma de Honduras. Patients were enrolled and blood collected after obtaining informed consent from patients or their surrogates (parental permission for minors).

##### Nicaragua Samples

From December 2015, children enrolled in the Nicaraguan Pediatric Dengue Cohort Study, a community-based prospective study of children 2 to 14 years of age that has been ongoing since August 2004 in Managua, Nicaragua (Kuan et al., 2009), were screened for Zika virus infection. Participants present to the Health Center Sócrates Flores Vivas at the first sign of illness and are followed daily during the acute phase of illness. Acute and convalescent (∼14-21 days after onset of symptoms) blood samples are drawn for dengue (DENV), chikungunya (CHIKV) and Zika virus diagnostic testing. The case definition for DENV or ZIKV virus infection for children presenting with an undifferentiated febrile illness or rash with one or more of the following signs and symptoms: conjunctivitis, arthralgia, myalgia, and/or periarticular edema regardless of fever.

The Institutional Review Boards of the Nicaraguan Ministry of Health and the University of California, Berkeley approved the study. Parents or legal guardians of all subjects provided written informed consent and subjects ≥6 years old provided assent. ZIKV samples from Nicaragua were de-identified prior to analysis and are considered exempt from human subject regulations.

Most patients in this study were enrolled through passive outbreak surveillance programs and therefore were not selected on basis of gender, gender identity or developmental stage. Samples from Nicaragua were obtained from children enrolled in a pediatric dengue study who were under 14 years of age. Gender information and information about whether patients were involved in previous procedures, or whether they were drug or test naïve, is not available. These variables have little or no relevance to the regional characterisation of ZIKV genetic diversity undertaken in this study. Further, human samples were de-identified prior to the viral characterisation and analysis presented in this study and therefore patient information cannot be reported. There were no inclusion or exclusion criteria for subjects other than those described above.

### Method Details

#### Diagnosis and Viral RNA Isolation

##### Mexico Samples

ZIKV diagnosis by real-time quantitative reverse transcription PCR (qRT-PCR) was made according to guidelines from the National Institute of Diagnosis and Epidemiological Reference (InDRE) of Mexico. Forward and reverse primers (ZIKV 1086 and ZIKV 1162c, respectively) and *Carboxyfluorescein* (FAM)-labeled probes (ZIKV 1107-FAM) were used as described by Lanciotti et al., 2016 (CDC Monoplex assay). Viral RNA was extracted from 200μL of patient serum using the QiAmp Viral RNA Extraction Mini Kit (Qiagen, Hilden, Germany). The presence of ZIKV RNA was evaluated using QuantiTec Probe RT-PCR kit (Qiagen). Each reaction consisted of 12.5 μL of 2x reverse transcription master mix, 0.5 μL of QuantiTect RT mix, 0.25μL of each primer (1μM final concentration), 0.15μL of probe (0.15μM final concentration), 6.35 μL of water and 5μL of RNA. Using the Applied Biosystems 7500 Fast system (Applied Biosystems, Foster City, USA) reverse transcription was carried out at 50°C for 30 mins followed by 95°C for 10 minutes and 45 cycles of 95°C for 15 seconds and 69°C for 1 minute. A few ZIKV samples that were borderline positive in Mexico at the time of initial screening were subsequently found to be negative upon repeat testing immediately prior to sequencing (Table S1, “No Ct” samples).

##### Returning Travelers and Honduras Samples

Viral nucleic acids were extracted using the EZ1 Virus Mini Kit v2.0 (Qiagen), and RNA was reverse transcribed using Superscript III Reverse Transcription Kit (Invitrogen). Nucleic acid extracts were subjected to DNase treatment at 37°C for 30 minutes using Turbo DNase (Thermo-Fisher Scientific) and Baseline-ZERO DNase (Epicentre), followed by qRT-PCR testing for ZIKV as described above and as previously described by Lanciotti et al., 2016. RNA integrity was assessed using RNA 6000 Pico kit on the Bioanalyzer (Agilent). A few ZIKV samples that were borderline positive at the CDPH at the time of initial screening were subsequently found to be negative upon repeat qRT-PCR testing immediately prior to sequencing (Table S1, “No Ct” samples).

##### Nicaragua Samples

Viral RNA of suspected ZIKV cases were extracted with the QiAmp Viral RNA Extraction Mini Kit (Qiagen, Hilden, Germany) by using 140 ìL of serum and a 60ìL elution volume. The presence of ZIKV RNA was tested by qRT-PCR using triplex assays (ZCD (Waggoner et al., 2016) and CDC Trioplex (Santiago et al., 2018) assays, which simultaneously screen for DENV, CHIKV and ZIKV infections. In some cases the CDC ZIKV Monoplex assay was also used, as described above, and as previously described (see Lanciotti et al., 2016). The ZCD assay qRT-PCR reactions were performed with primers and probes previously described by Waggoner et al., 2016, using 25ìL master mix reactions of the SuperScript III Platinum One-Step qRT-PCR kit (Invitrogen) and 5 ìL of RNA. Cycling conditions for the ZCD assay were as follows: 52°C for 15 minutes; 94°C for 2 minutes; 45 cycles at 94°C for 15 seconds, 55°C for 20 seconds (acquisition), and 68°C for 20 seconds (Waggoner et al., 2016). The qRT-PCR reactions of the CDC Trioplex used primers and probes described by Lanciotti et al., 2016. The reactions were assembled by mixing 10 μL of sample RNA with 12.5 μL of PCR master mix reaction buffer (SuperScript III), virus-specific primers to a final concentration of 1 μM. Thermocylcling protocols were as follows: reverse transcription (RT) at 50 °C for 30 min, RT inactivation at 95 °C for 2 min, fluorescence detection at 95 °C for 15 s, and annealing at 60 °C for 1 min (Santiago et al., 2018).

#### Zika Virus Primer Design

Short 13-mer primers were designed using an in-house developed computational algorithm. Briefly, a multiple sequence alignment of the 44 ZIKV reference genomes available in the National Center for Biotechnological Information (NCBI) GenBank at the time of the design (March 2016) was performed using MAFFT software (Katoh and Standley, 2013). The consensus sequence was then partitioned into 250-nt segments, followed by automated selection of reverse 13-nt primers within 50-nt windows at the edges of each segment. Primers were designed by the algorithm according to the following criteria: (i) no degeneracy, (ii) no self-dimers or cross-dimers with hybridization ΔG < -9 kcal/mol, (iii) no homopolymer repeats >5 nt in length, and (iv) ranked by number of segments covered. Additional primers were designed manually at the 3’ end of the consensus sequence using Primer3 (Untergasser et al., 2012). The complete 13-mer ZIKV primer set consisted of 51 reverse primers (Table S3).

#### Construction of Metagenomic Libraries

Metagenomic next-generation sequencing (mNGS) libraries were prepared using a modified protocol similar to that previously described (Naccache et al., 2016). Briefly, DNase-treated extracted RNA was reverse-transcribed to cDNA using a mixture of 13-mer ZIKV spiked primers and random hexamer primers at a 5:1 ratio, followed by library preparation using the Nextera XT kit (Illumina).

#### mNGS and Bait Capture Probe Enrichment

Individual sample libraries were dual-index barcoded and pooled into sets of 8-12 for sequencing on a HiSeq 2500 instrument (Illumina). To assess for potential ZIKV cross-contamination, we included a negative “no-template control” sample (consisting of extraction buffer alone) that was processed and sequenced in parallel for each run. No reads mapping to ZIKV were found in any of the sequenced negative control samples. Libraries were sequenced as 150 base pair (bp) paired-end runs on a HiSeq 2500 instrument (Illumina). Data was scanned for ZIKV reads using the SURPI (sequence-based ultra-rapid pathogen identification) computational pipeline (Naccache et al., 2014) and direct NCBI BLASTn (Altschul et al., 1997) alignment to ZIKV reference genome KJ776791 at an e-value threshold of 1x10^-8^. Metagenomic ZIKV reads were then mapped on KJ776791 reference genome using bwa-mem program (Li and Durbin, 2009, Li, 2013) and GATK (McKenna et al., 2010) was used to perform variant calling and generate consensus sequences with a 3x minimum read depth coverage.

A subset of mNGS libraries were enriched for ZIKV sequences using xGen biotinylated lockdown bait capture probes (Integrated DNA Technologies) designed to tile across all 44 sequenced ZIKV genomes in GenBank as of March 2016 (Table S4). Capture probes were curated for redundancy at a 99% nucleotide similarity cutoff using CD-HIT (Li and Godzik, 2006). Enrichment was performed on the mNGS libraries in pools of 8 libraries (including ZIKV–negative serum samples as controls) using the xGen lockdown probe protocol and the SeqCap EZ Hybridization and Wash Kit (Roche).

A subset of ZIKV infected serum collected from 14 subjects residing in Nicaragua were sequenced using a separate bait capture method previously described for Hepatitis C virus (Bonsall et al., 2015). Total RNA-seq libraries were prepared using the NEB Ultra Directional library kit with adaptations to the manufacturer’s protocol as previously described (Bonsall et al., 2015). By this method, RNA was heat-fragmented, reverse-transcribed using random hexamers then ligated to adapters that bind the manufacturers barcoded-PCR primers. Equal masses of amplified libraries are pooled for hybridization to a mixture of biotinylated 120mer oligonucleotides derived from 60 mer overlapping windows of the complete genome of the ZIKV strain KJ776791 (Integrated DNA Technologies) and captured with streptavidin-conjugated beads (Nimblegen) then PCR amplified to produce the final library for sequencing. The final library was sequenced using a MiSeq (Illumina) instrument using v3 chemistry producing 150 nt paired-ends reads. Reads were mapped on KJ776791 reference genome using bwa-mem program (Li and Durbin, 2009, Li, 2013) and GATK (McKenna et al., 2010) was used to perform variant calling and generate consensus sequences with a 3x minimum read depth coverage.

#### Sequence Alignment

Published and available ZIKV coding sequences of the Asian genotype longer than 1500 nucleotides were retrieved from GenBank database as of June 2017. These 298 sequences were aligned together with the ZIKV sequences generated here using MAFFT (Katoh and Standley, 2013) and manual editing.

#### Maximum Likelihood Phylogenetic Analysis

A maximum likelihood (ML) phylogeny was estimated from this alignment using PhyML (Guindon and Gascuel, 2003) under a general time reversible nucleotide substitution model, with a gamma distributed among site rate variation and a proportion of invariant sites (GTR + Γ + I), as determined by jModelTest2 (Darriba et al., 2012). Statistical support for nodes of the ML phylogeny was assessed using a bootstrap approach with 100 replicates.

#### Molecular Clock and Phylogeographic Analyses

Temporal evolutionary signal in our alignment was evaluated using TempEst (Rambaut et al., 2016), which plots sample collection dates against root-to-tip genetic distances obtained from the ML phylogeny (see above). The plot indicated that the data set contained sufficient temporal signal for molecular clock analysis. Molecular clock phylogenies were estimated using the Bayesian MCMC approach implemented in BEAST v1.8.4 (Drummond et al., 2012). We computed 4 independent runs of 100 million MCMC steps, sampling parameters and trees every 5000 steps. An uncorrelated lognormal relaxed molecular clock model (Drummond et al., 2006) and a Bayesian skyline coalescent model (Drummond, 2005) were used; previous studies have demonstrated this combination to be the best fitting model combination for ZIKV in the Americas (Faria et al., 2017, Grubaugh et al., 2017, Metsky et al., 2017). In each run, a SRD06 substitution model (Shapiro et al., 2006) was used, which employs a Hasegawa, Kishino and Yano nucleotide substitution model, a gamma distribution among site rate variation (HKY+Γ) and a codon position partition (positions (1+2) versus position 3). A non-informative continuous-time Markov chain (CTMC) reference prior (Ferreira and Suchard, 2008) was placed on the molecular clock rate for all analyses. The program Tracer v1.7 (Rambaut et al., 2018) was used to evaluate MCMC chain convergence and to compute marginal posterior distributions of parameters, after removal of 10% of the chain as burn-in. The program logcombiner was used to combine and subsample posterior tree distributions, after a 10% burn-in, thereby generating an empirical distribution of 1,500 molecular clock trees.

This empirical tree distribution was then used in subsequent phylogeographic analyses to infer ancestral branch locations using the Bayesian asymmetric discrete trait evolution model (Lemey et al., 2009) implemented in BEAST v1.8.4 (Drummond et al., 2012). To account for the possibility of sampling bias that may arise from a larger number of sequences from particular locations, we performed eleven phylogeographic analyses using (i) the full dataset (n = 359) and (ii) ten jackknife resampled datasets (n = 97) in which taxa from each location were randomly sub-sampled to ten sequences. We counted lineage movement events among pairs of discrete locations using the robust counting approach (Minin and Suchard, 2008, O'Brien et al., 2009). An in-house script was used to identify the earliest estimated ZIKV introductions into distinct locations from the results of the robust counting method. Viral lineage movement events were statistically supported (with Bayes factors >3) using the BSSVS (Bayesian stochastic search variable selection) approach (Lemey et al., 2009), as implemented in BEAST v1.8.4 (Drummond et al., 2012). TreeAnnotator was used to generate a summary maximum clade credibility (MCC) tree from the posterior distribution of trees (after removal of MCMC burn-in of 10%). The MCC phylogeny was drawn using the using ggtree package (Yu et al., 2016) of the R software platform (http://www.R-project.org/). Box plots for node ages were generated using the ggplot2 package (Wickham, 2016).

#### Birth-Death Skyline Analyses

We analyzed the 104 sequences comprising the Central American clade (clade B, Figure 2A) using the serially sampled birth-death skyline model (Stadler et al., 2013), implemented in BEAST2 v2.4.7 (Bouckaert et al., 2014). We computed 2 independent runs of 100 million MCMC steps and sampled parameters every 10,000 steps. In each run, an uncorrelated lognormal relaxed clock model (Drummond et al., 2006) and a SRD06 substitution model (Shapiro et al., 2006) were used, as in the phylogeographic analyses, above. An informative lognormal prior was placed on the molecular clock rate parameter, with mean equal to the median rate from the phylogeographic analyses and standard deviation set to include its 95% highest posterior densities (HPDs). A Laplace distribution was placed on the date of the MRCA with mean equal to the median estimated date in the phylogeographic analyses and scale parameter set to include its 95% HPDs. A lognormal prior with mean of 0 and standard deviation of 1.25 was placed on the effective reproductive number parameter (R_e_). A Beta prior with α and β set to 1 and 999, respectively, was placed on the sampling proportion. The rate at which patients recover (becoming non-infectious rate) was fixed to 18.25, which corresponds to a mean infectious period of 20 days (this was based on the estimated mean generation time for ZIKV estimated by Ferguson et al., 2016). The origin time of the Central American epidemic was bounded to be no older than March 1, 2014. A lognormal prior with mean equal to March 1, 2014 and standard deviation of 1 was also placed on the origin time.

The R_e_ parameter was allowed to change at 9 time points, equally spaced between the TMRCA and the time of the most recent sample. The sampling proportion parameter was assumed to be 0 before the time of the oldest sample and allowed to change at 9 time points, equally spaced between the oldest and most recent samples. The rate at which individuals become non-infectious rate was assumed to be constant through time. To assess the robustness of the estimates of R_e_ with respect to prior assumptions about the sampling proportion we repeated the above analyses with a sampling proportion prior favoring a lower sampling proportion (Beta distribution with α =1, β = 9999) and a higher sampling proportion (Beta distribution with α =2, β = 99).

The program Tracer v1.7 (Rambaut et al., 2018) was used to check MCMC chain convergence and logcombiner was used to combine and subsample posterior distributions, after the removal of 25% of the chains as burn-in. Figures were produced using the R software platform using in-house scripts and the R-package bdskytools (available at https://github.com/laduplessis/bdskytools).

#### Climatic Vector Suitability Scores

To predict for seasonal variation in the geographical distribution of the ZIKV vector *Aedes aegypti* in Central America and Mexico we used monthly *Aedes aegypti* suitability maps at a 5km x 5km spatial resolution (Bogoch et al., 2016, Faria et al., 2017). We then aggregated these high resolution maps at the country level.

#### Spatial Analyses

Geographical information system (GIS)-based maps were generated using the open source QGIS software (https://qgis.org). Data for population density and elevation were downloaded from the Worldpop (http://www.worldpop.org.uk/) and CGIAR-CSI (http://srtm.csi.cgiar.org/) website projects, respectively. *Aedes aegypti* predicted climatic suitability maps at a 5km x 5km spatial resolution were extracted from Faria et al., 2017.

### Quantification and Statistical Analysis

#### Maximum Likelihood Phylogenetic Analysis

To assess the suitability of substitution models for our ZIKV alignment (Data S1) we performed a statistical model selection procedure based on the Akaike information criterion, using jModelTest2 (Darriba et al., 2012). This identified the best fitting substitution model (GTR + Γ + I) for ML phylogenetic analysis. A phylogenetic bootstrap analysis with 100 replicates using PhyML (Guindon and Gascuel, 2003) was conducted to evaluate the statistical support for nodes of the ML phylogeny (Figure S2). In phylogenetic analyses, *n* refers to the number of viral gene/genome sequences.

#### Molecular Clock and Phylogeographic Analyses

To assess whether our data was suitable for a molecular clock phylogenetic analysis, we evaluated the temporal evolutionary signal in our ZIKV alignment using the statistical approaches in TempEst (Rambaut et al., 2016). A linear regression bewteen sample collection dates and root-to-tip genetic distances obtained from the ML phylogeny (Figure S2) indicated that the feasibility of a molecular clock approach. A Bayesian MCMC approach implemented in BEAST v1.8.4 (Drummond et al., 2012) was used to infer molecular clock phylogenies (Figure 2A), using an uncorrelated lognormal relaxed molecular clock model (Drummond et al., 2006), a Bayesian skyline coalescent model (Drummond, 2005), a SRD06 substitution model (Shapiro et al., 2006) and with a CTMC reference prior placed on the molecular clock rate for all analyses. A Bayesian asymmetric discrete trait evolution model (Lemey et al., 2009) implemented in BEAST v1.8.4 (Drummond et al., 2012) was used to infer ancestral branch locations in the molecular clock phylogenies (Figure 2A). A robust counting (Minin and Suchard, 2008, O'Brien et al., 2009) approach was used to count lineage movement events among pairs of discrete locations (Figure 2B) and a BSSVS measure (Lemey et al., 2009), was used to estimate their statistical supports.

#### Birth-Death Skyline Analyses

The serially sampled birth-death skyline model (Stadler et al., 2013), implemented in BEAST2 v2.4.7 (Bouckaert et al., 2014) was used to estimate the effective reproductive number (R_e_) and the sampling proportion of ZIKV in CAM (Figure 2C), using an uncorrelated lognormal relaxed clock model (Drummond et al., 2006), a SRD06 substitution model (Shapiro et al., 2006), with an informative lognormal prior placed on the molecular clock rate and a Laplace distribution placed on the date of the MRCA, spanning mean rate and standard deviation of both parameters from the phylogeographic analyses. A lognormal prior with mean of 0 and standard deviation of 1.25 was placed on the effective reproductive number parameter (R_e_), a Beta prior with α and β set to 1 and 999, respectively, was placed on the sampling proportion and the rate at which patients recover was fixed to 18.25.

#### Climatic Vector Suitability Scores

A linear regression model, developed and described in Faria et al., (2017), was used to assess the correlation between monthly *Aedes aegypti* predicted climatic suitability and the number of weekly ZIKV notified cases, for each Central America country and for Mexico. This model tests how well vector suitability explains the variation in the number of ZIKV notified cases (Table S2).

### Data and Software Availability

Genome sequences generated in this study are publicly available in GenBank database under the accession numbers: KY606271-KY606274, MF434516-MF434522 and MF801377-MF801426.

## Supplemental Information

Document S1. Figures S1–S4 and Tables S1–S4

Data S1. Alignment Used in Phylogenetic Analyses, Related to Figure 2A The alignment in fasta format includes 298 publicly available Zika virus sequences and 61 Zika virus sequences generated in this study.

Document S2. Article plus Supplemental Information
