# Supplementary material for: Genomic Epidemiology Reconstructs the Introduction and Spread of Zika Virus in Central America and Mexico
Source: Cell Host Microbe. 2018 Jun 13;23(6):855–864.e7. doi: 10.1016/j.chom.2018.04.017 (PMC6006413; doi:10.1016/j.chom.2018.04.017)
Supplement: Document S1. Figures S1–S4 and Tables S1–S4 [file mmc1.pdf]

Cell Host & Microbe, Volume *23*

Supplemental Information

Genomic Epidemiology Reconstructs the Introduction and Spread of Zika Virus in Central America and Mexico

Julien Thézé, Tony Li, Louis du Plessis, Jerome Bouquet, Moritz U.G. Kraemer, Sneha Somasekar, Guixia Yu, Mariateresa de Cesare, Angel Balmaseda, Guillermina Kuan, Eva Harris, Chieh-hsi Wu, M. Azim Ansari, Rory Bowden, Nuno R. Faria, Shigeo Yagi, Sharon Messenger, Trevor Brooks, Mars Stone, Evan M. Bloch, Michael Busch, José E. Muñoz-Medina, Cesar R. González-Bonilla, Steven Wolinsky, Susana López, Carlos F. Arias, David Bonsall, Charles Y. Chiu, and Oliver G. Pybus

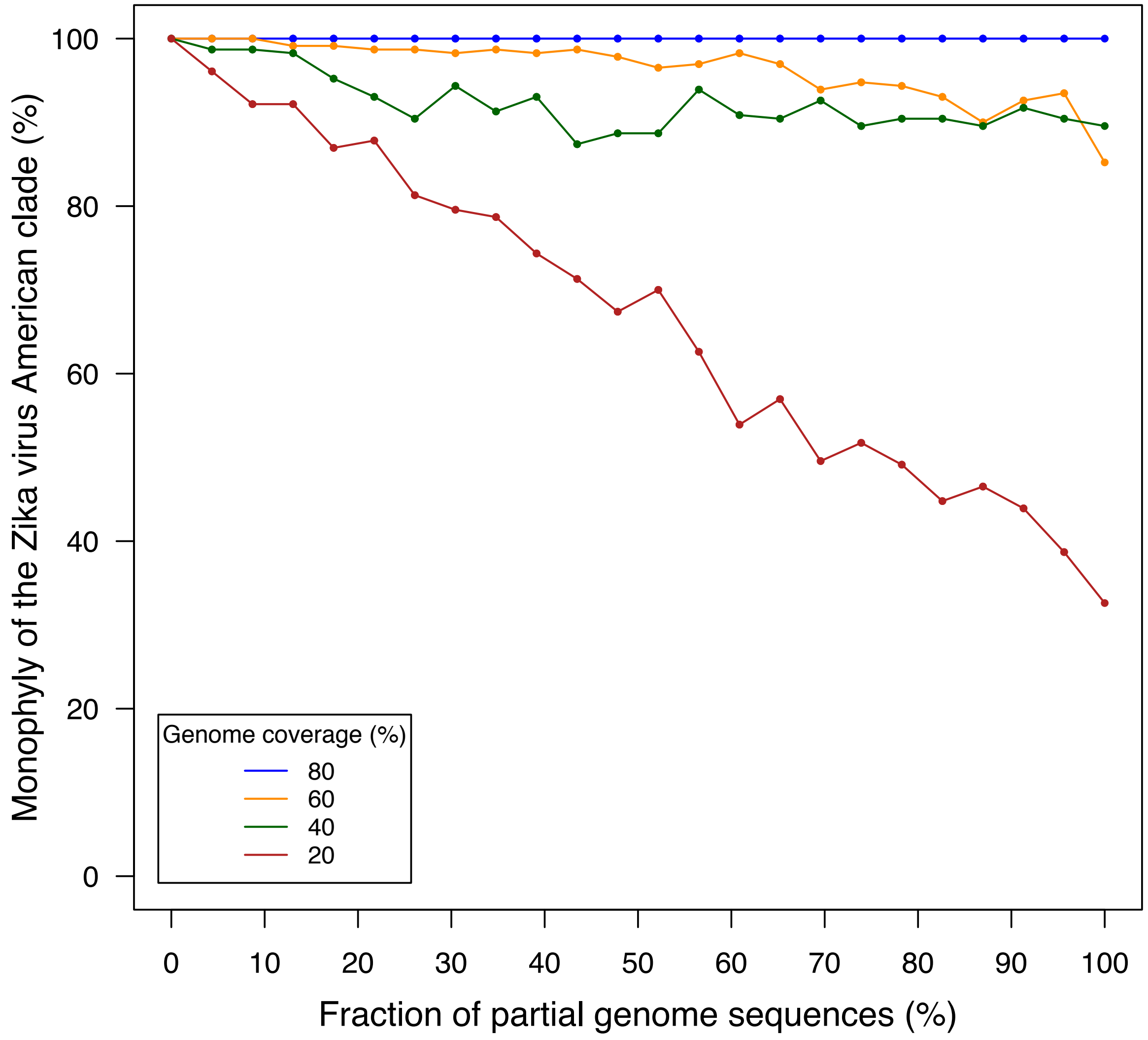

Figure S1. The effect of partial genome sequences on ZIKV phylogenetic inference (related to Figure 1 and Figure 2A). An initial dataset comprising 28 ZIKV complete genome sequences of the Asian genotype was generated (comprising 5 basal genomes from South East Asia and 23 genomes from the Americas, forming the American outbreak clade). Replicate datasets were derived from the initial dataset by subsampling one or more of the 23 American ZIKV genomes, as follows. For each genome that was subsampled, we reduced its coverage from 100% to 20, 40, 60, or 80% (colors; inset) by randomly mapping simulated reads to the genome until the required coverage was reached, then replacing nucleotides at the unmapped sites with the ambiguity code "N". The read positions and lengths of the simulated reads were chosen to be characteristic of metagenomic sequencing of clinical ZIKV samples. This procedure was then repeated by varying the number of subsampled genomes from 0 to 23 (x-axis). Therefore, for each combination of coverage and fraction of partial genomes (i.e. each dot in the figure), 230 replicate alignments were generated. Maximum likelihood trees were estimated from each replicate alignment and, for each, the percentage of trees in which the American ZIKV outbreak clade is inferred to be monophyletic is shown on the y-axis.

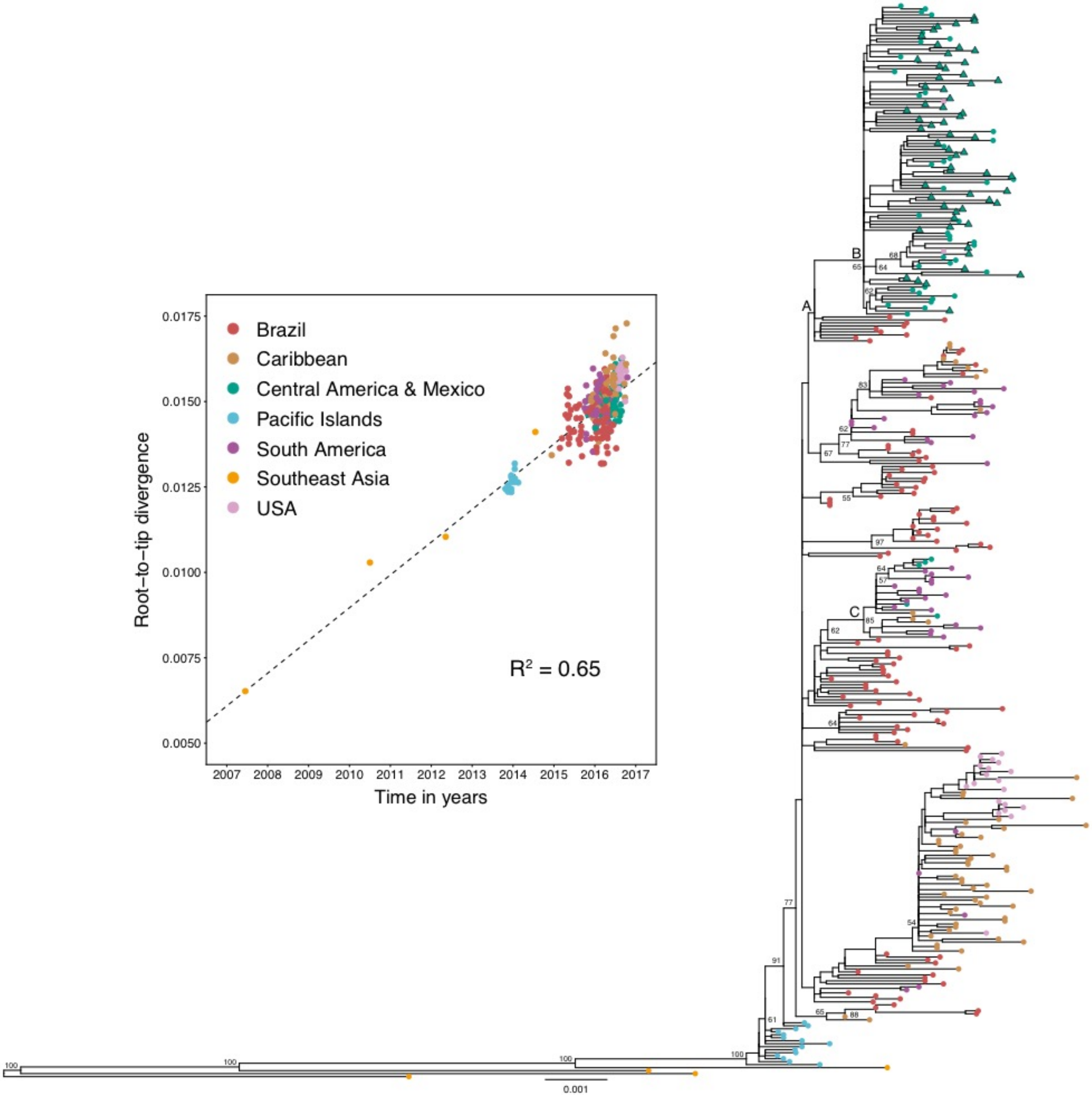

Figure S2. Maximum likelihood phylogeny and temporal signal of the Zika virus Asian genotype lineage (related to Figure 2A). The phylogeny was estimated using PhyML (Guindon and Gascuel, 2003) based on complete and partial (>1500 nt) coding genome sequences. Statistical supports for nodes were assessed using a bootstrap approach (100 replicates). Only bootstrap supports >50 at internal nodes are displayed. Colored symbols at phylogenetic tips denote sampling locations of the sequences. Circles and triangles denote sequences publicly available and sequences generated in this study, respectively. The regression plot shows the correlation between the sampling date of each sequence and the genetic distance of that sequence from the root of the phylogeny. Colored circles denote tips of the phylogeny with its corresponding sampling location.

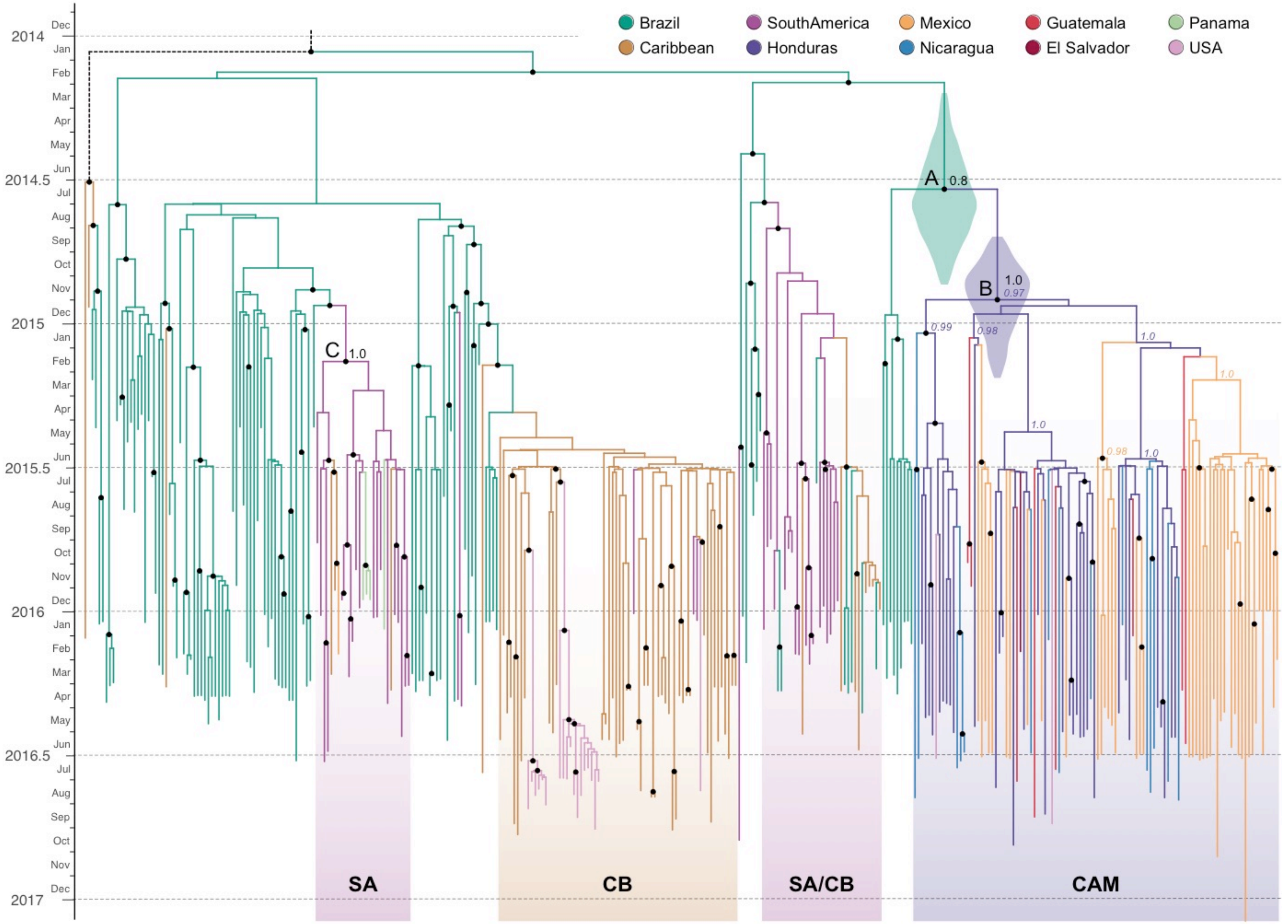

Figure S3. Maximum clade credibility phylogeny estimated from complete and partial ZIKV sequences from the Americas (related to Figure 2A). For visual clarity, basal Asian and Pacific lineages are not displayed. Violin plots show the posterior distributions of the estimated dates of nodes A and B (see main text). Branch colors indicate the most probable ancestral lineage locations. Circles at internal nodes denote clade posterior probabilities >0.75. For selected nodes, colored numbers show the posterior probabilities of inferred ancestral locations, whilst black numbers are the clade posterior probabilities. Clades from outside Brazil are highlighted and denoted CB (Caribbean), SA (South America), and CAM (Central America and Mexico).

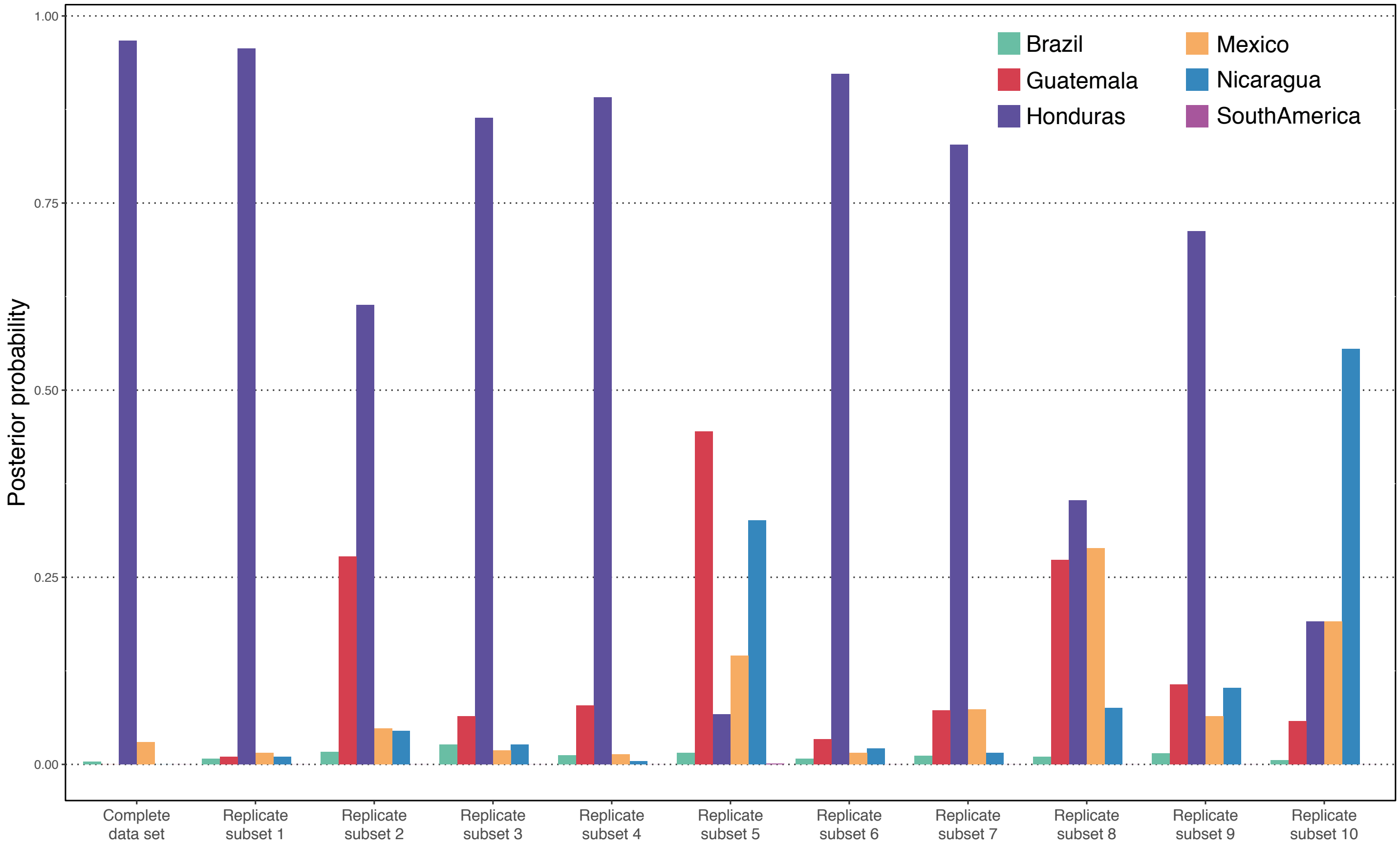

Figure S4. Ancestral node location posterior probabilities for node B (related to Figure 2A), estimated using the full dataset, and on ten jackknife resampled datasets (see STAR Methods for details). For each dataset, a Bayesian asymmetric discrete trait evolution model was used to estimate ancestral node locations.

Table S1. Sample and sequencing information (related to Figure 1)

Sequencing Method	Accession Number	Sample Name	Source	Sample type	Region	Collection Date	Days between symptom onset and date of sampling	ZIKV Ct value during sequencing (diagnostic Ct)	Illumina					Illumina with Probe Enrichment					Merged	Used in phylogenetic analyses
									Reads mapped	Average coverage depth	Deduplicated reads mapped	Deduplicated average coverage depth	Reference coverage (%)	Reads mapped	Average coverage depth	Deduplicated reads mapped	Deduplicated average coverage depth	Reference coverage (%)	Reference coverage (%)	
Bait capture mNGS	MF434516	1304.13.A.1	HCSFV	Serum	Nicaragua: Managua	2016-08-05	1	31	162,621	2,166.26	829	19.86	99.85	-	-			-	-	yes
	MF434517	1659.13.A.1	HCSFV	Serum	Nicaragua: Managua	2016-08-19	2	28	131,556	1,785.42	11,065	154.78	99.99	-	-			-	-	yes
	NA	4445.13.A.1	HCSFV	Serum	Nicaragua: Managua	2016-08-03	2	28	19	1.09	19	1.09	4.61	-	-			-	-	no
	NA	4858.13.A.1	HCSFV	Serum	Nicaragua: Managua	2016-07-13	3	35	5	1.00	5	1.00	1.40	-	-			-	-	no
	MF434518	5256.13.A.1	HCSFV	Serum	Nicaragua: Managua	2016-08-26	1	36	145,718	2,072.49	269	6.23	92.60	-	-			-	-	yes
	MF434519	5338.13.A.1	HCSFV	Serum	Nicaragua: Managua	2016-07-22	1	29	14,369	325.58	91	2.04	58.96	-	-			-	-	yes
	NA	5376.13.A.1	HCSFV	Serum	Nicaragua: Managua	2016-07-07	2	36	4	1.00	4	1.00	1.68	-	-			-	-	no
	NA	5604.12.B.1	HCSFV	Serum	Nicaragua: Managua	2016-02-07	4	26	9	1.33	9	1.33	2.28	-	-			-	-	no
	NA	5660.13.A.1	HCSFV	Serum	Nicaragua: Managua	2016-07-27	2	32	4,088	304.40	18	1.33	16.05	-	-			-	-	no
	MF434520	5847.12.B.1	HCSFV	Serum	Nicaragua: Managua	2016-02-07	1	39	258,362	2,564.90	202	4.72	53.18	-	-			-	-	yes
	NA	5953.13.A.1	HCSFV	Serum	Nicaragua: Managua	2016-08-11	1	37	704	35.41	36	1.79	26.93	-	-			-	-	no
	MF434521	6547.13.A.1	HCSFV	Serum	Nicaragua: Managua	2016-07-19	1	21	158,162	2,142.25	5,661	83.79	100.00	-	-			-	-	yes
	NA	7208.13.A.1	HCSFV	Serum	Nicaragua: Managua	2016-07-15	1	38	20	1.38	20	1.38	6.33	-	-			-	-	no
	MF434522	7253.13.A.1	HCSFV	Serum	Nicaragua: Managua	2016-08-29	1	32	41,299	554.15	3,803	52.75	99.96	-	-			-	-	yes
Spiked primers mNGS	MF801381	CDPH_CentralAm-24	CDPH	urine	Honduras	2016	NA	32	6,846	66.15	2,185	22.30	99.14	-	-			-	-	yes
	MF801380	CDPH_CentralAm-2411	CDPH	urine	Guatemala	2016-04-11	NA	39	844	9.12	366	4.14	76.46	-	-			-	-	yes
	MF801382	CDPH_CentralAm-25	CDPH	urine	Honduras	2016	NA	28	408	5.15	241	3.20	82.24	-	-			-	-	yes
	NA	CDPH_CentralAM-3263	CDPH	serum	Guatemala	2016-04-11	NA	38	123	4.37	32	1.24	26.05	-	-			-	-	no
	MF801426	CDPH_CentralAM-3912	CDPH	urine	Nicaragua	2016-07-25	NA	29	89,647	844.26	4,826	70.40	99.34	-	-			-	-	yes
	MF801378	CDPH_CentralAM-3921	CDPH	urine	Guatemala	2016-07-21	NA	33	10,510	88.46	1,889	20.21	99.27	-	-			-	-	yes
	NA	CDPH_ElSalvador-0S28	CDPH	serum	El Salvador	2016-02-22	NA	No Ct (37)	-	-	-	-	-	-	-			-	-	NA
	MF801377	CDPH_ElSalvador-1055	CDPH	urine	El Salvador	2016-02-22	NA	35	656	9.43	131	2.20	71.47	-	-			-	-	yes
	NA	CDPH_ElSalvador-S261	CDPH	serum	El Salvador	2016-02-03	NA	No Ct (38)	-	-	-	-	-	-	-			-	-	NA
	NA	CDPH_Guatemala-S840	CDPH	urine	Guatemala	2016-02-11	NA	39	-	-	-	-	12.00							no
	MF801379	CDPH_Guatemala-3294	CDPH	urine	Guatemala	2016-06-18	NA	35	171	5.01	78	2.47	35.94	-	-			-	-	yes
	NA	CDPH_Guatemala-4516	CDPH	serum	Guatemala	2016-06-19	NA	41	548	151.93	6	1.68	4.59	-	-			-	-	no
	NA	CDPH_Guatemala-4530	CDPH	serum	Guatemala	2016-06-18	NA	40	129	7.18	21	1.35	15.86	-	-			-	-	no
	NA	CDPH_Guatemala-S559	CDPH	serum	Guatemala	2016-02-17	NA	No Ct (37)	-	-	-	-	-	-	-			-	-	NA
	MF801383	CDPH_Honduras-4102	CDPH	serum	Honduras	2016-06-03	NA	33	4,567	38.85	907	8.35	94.84	-	-			-	-	yes
	NA	CDPH_Mexico_S085	CDPH	serum	Mexico: Chiapas	2016-01-24	NA	37	-	-	-	-	16.92							no
	MF801390	CDPH_Mexico-1	CDPH	urine	Mexico	2016	NA	36	229	4.02	131	2.55	51.53	-	-			-	-	yes
	NA	CDPH_Mexico-20	CDPH	urine	Mexico	2016	NA	Not Ct (37)	-	-	-	-	-	-	-			-	-	NA
	NA	CDPH_Mexico-21	CDPH	urine	Mexico	2016	NA	31	619	29.08	46	3.96	5.02	-	-			-	-	no
	NA	CDPH_Mexico-22	CDPH	urine	Mexico	2016	NA	36	28	14.48	4	2.57	24.94	-	-			-	-	no
	NA	CDPH_Mexico-23	CDPH	urine	Mexico	2016	NA	34	96	13.89	11	1.49	2.15	-	-			-	-	no
	MF801391	CDPH_Mexico-3502	CDPH	urine	Mexico: Oaxaca	2016-07-06	NA	31	17,002	196.13	700	15.28	97.58	-	-			-	-	yes
	MF801392	CDPH_Mexico-4227	CDPH	urine	Mexico	2016-08-13	NA	34	136	3.79	42	1.46	30.39	-	-			-	-	yes
	NA	CDPH_Mexico-5	CDPH	urine	Mexico	2016	NA	37	45	10.30	8	1.32	8.67	-	-			-	-	no
	MF801425	CDPH_Nicaragua-3358	CDPH	urine	Nicaragua	2016-06-24	NA	34	3,059	78.68	92	5.35	47.60	-	-			-	-	yes
	MF801384	Hond-1031	BSRI	Serum	Honduras: Roatán	2016-02-03	1	26	4,462	39.57	1,925	18.98	98.10	-	-			-	-	yes
	MF801385	Hond-1055	BSRI	Serum	Honduras: Roatán	2016-02-12	1	30	704	8.07	400	4.79	82.43	-	-			-	-	yes
	MF801386	Hond-1083	BSRI	Serum	Honduras: Roatán	2016-02-24	4	20	29,644	303.93	1,048	32.73	96.81	-	-			-	-	yes
	MF801387	Hond-1122	BSRI	Serum	Honduras: Roatán	2016-03-29	2	23	15,511	148.90	2,086	28.45	99.18	-	-			-	-	yes
	MF801388	Hond-1138	BSRI	Serum	Honduras: Roatán	2016-04-25	1	32	1,985	73.93	59	8.33	29.40	-	-			-	-	yes
	MF801389	Hond-1162	BSRI	Serum	Honduras: Roatán	2016-05-10	2	29	1,944	19.23	1,178	12.03	94.62	-	-			-	-	yes
	MF801393	mex01	CLE	Serum	Mexico: Chiapas	2016-04-22	2	37	4,142	68.73	444	7.24	77.36	-	-			-	-	yes
	NA	mex02	CLE	Serum	Mexico: Chiapas	2016-04-29	3	40	620	425.10	4	4.51	1.83	-	-			-	-	no
	KY606271	mex03	CLE	Serum	Mexico: Chiapas	2016-05-03	2	35	3,715	42.25	1,365	16.13	99.65	-	-			-	-	yes
	MF801394	mex04	CLE	Serum	Mexico: Chiapas	2016-05-03	4	34	31,022	676.55	231	23.33	61.00	-	-			-	-	yes
	MF801395	mex05	CLE	Serum	Mexico: Chiapas	2016-05-09	1	34	10,769	136.99	3,183	40.37	99.09	-	-			-	-	yes
	NA	mex06	CLE	Serum	Mexico: Chiapas	2016-05-06	4	No Ct (33)	-	-	-	-	-	-	-			-	-	NA
	KY606272	mex07	CLE	Serum	Mexico: Oaxaca	2016-05-11	5	32	14,099	187.73	3,343	46.96	100.00	-	-			-	-	yes
	NA	mex08	CLE	Serum	Mexico: Chiapas	2016-05-11	2	No Ct (40)	-	-	-	-	-	-	-			-	-	NA
	NA	mex09	CLE	Serum	Mexico: Guerrero	2016-05-16	3	40	475	119.72	12	2.91	5.34	-	-			-	-	no
	NA	mex10	CLE	Serum	Mexico: Chiapas	2016-05-12	3	37	1,908	218.62	28	3.26	11.64	-	-			-	-	no
	NA	mex11	CLE	Serum	Mexico: Chiapas	2016-05-16	5	No Ct (33)	-	-	-	-	-	-	-			-	-	NA
	NA	mex12	CLE	Serum	Mexico: Chiapas	2016-05-16	2	37	352	235.71	4	2.68	2.08	-	-			-	-	no
	NA	mex13	CLE	Serum	Mexico: Chiapas	2016-05-16	1	No Ct (34)	-	-	-	-	-	-	-			-	-	NA
	MF801396	mex14	CLE	Serum	Mexico: Chiapas	2016-05-17	3	37	2,081	28.10	681	9.59	98.40	-	-			-	-	yes
	NA	mex15	CLE	Serum	Mexico: Guerrero	2016-05-20	3	41	42	2.01	27	1.31	25.78	-	-			-	-	no
	MF801397	mex16	CLE	Serum	Mexico: Campeche	2016-05-20	2	38	715	9.42	402	5.36	97.16	-	-			-	-	yes
	MF801398	mex17	CLE	Serum	Mexico: Chiapas	2016-05-20	3	35	2,434	31.34	1,343	17.69	99.22	-	-			-	-	yes
	NA	mex18	CLE	Serum	Mexico: Chiapas	2016-05-24	1	No Ct (31)	-	-	-	-	-	-	-			-	-	NA
	MF801399	mex19	CLE	Serum	Mexico: Chiapas	2016-05-20	5	37	406	6.22	150	2.38	85.25	-	-			-	-	yes
	MF801400	mex20	CLE	Serum	Mexico: Chiapas	2016-05-20	3	35	1,227	17.70	377	5.56	93.05	-	-			-	-	yes
	NA	mex21	CLE	Serum	Mexico: Chiapas	2016-05-21	0	No Ct (30)	-	-	-	-	-	-	-			-	-	NA
	NA	mex22	CLE	Serum	Mexico: Chiapas	2016-05-23	3	38	23	1.75	14	1.06	17.57	-	-			-	-	no
	MF801401	mex23	CLE	Serum	Mexico: Chiapas	2016-05-23	1	35	31,247	423.50	860	23.09	93.64	-	-			-	-	yes
	MF801402	mex24	CLE	Serum	Mexico: Chiapas	2016-05-26	1	36	2,614	33.76	1,293	17.05	99.59	-	-			-	-	yes
	MF801403	mex25	CLE	Serum	Mexico: Chiapas	2016-05-25	4	34	150,375	1,965.38	2,402	73.63	98.65	-	-			-	-	yes
	MF801404	mex26	CLE	Serum	Mexico: Guerrero	2016-07-08	4	38	2,823	41.57	462	7.32	72.35	232,891	2,891.04	126	31.93	67.23	77.62	yes
	MF801405	mex27	CLE	Serum	Mexico: Guerrero	2016-07-09	3	37	3,070	76.11	159	4.70	39.99	-	-			-	-	yes
	MF801406	mex28	CLE	Serum	Mexico: Oaxaca	2016-07-07	1	36	23,110	250.45	5,636	66.58	99.03	-	-			-	-	yes
	MF801407	mex29	CLE	Serum	Mexico: Oaxaca	2016-07-05	3	40	4,311	70.84	316	6.49	62.07	671,401	3,509.37	152	9.96	67.17	73.54	yes
	MF801408	mex30	CLE	Serum	Mexico: Chiapas	2016-07-05	1	37	32,580	372.80	1,555	29.63	96.55	-	-			-	-	yes
	MF801409	mex31	CLE	Serum	Mexico: Chiapas	2016-07	4	36	8,519	226.10	156	12.07	44.28	-	-			-	-	yes
	MF801410	mex32	CLE	Serum	Mexico: Guerrero	2016-07-05	2	33	71,930	772.50	11,867	144.59	99.13	-	-			-	-	yes
	MF801411	mex33	CLE	Serum	Mexico: Guerrero	2016-07-05	2	37	26,604	339.18	720	16.32	92.00	-	-			-	-	yes
	MF801412	mex34	CLE	Serum	Mexico: Guerrero	2016-07-07	1	36	22,940	243.15	2,793	34.41	98.98	-	-			-	-	yes
	MF801413	mex35	CLE	Serum	Mexico: Guerrero	2016-07-07	2	33	220,724	2,437.18	9,012	155.98	99.37	-	-			-	-	yes
	MF801414	mex36	CLE	Serum	Mexico: Guerrero	2016-07-05	3	33	228,940	2,514.78	13,551	208.19	99.29	-	-			-	-	yes
	MF801415	mex37	CLE	Serum	Mexico: Guerrero	2016-07-05	2	38	5,517	94.61	344	7.66	60.23	646,950	4,493.69	135	80.99	61.73	68.10	yes
	MF801416	mex38	CLE	Serum	Mexico: Guerrero	2016-07	1	37	10,460	265.93	137	7.04	51.09	-	-			-	-	yes
	KY606274	mex39	CLE	Serum	Mexico: Guerrero	2016-07-07	3	36	41,401	495.93	812	30.82	94.97	-	-			-	-	yes
	MF801417	mex40	CLE	Serum	Mexico: Chiapas	2016-07-01	2	35	360,099	3,875.28	4,574	162.44	98.93	-	-			-	-	yes
	NA	mex41	CLE	Serum	Mexico: Chiapas	2016-07-01	1	40	256	7.05	90	2.81	24.90	18,739	7,870.20	1	0.99	1.51	24.90	no
	MF801418	mex42	CLE	Serum	Mexico: Chiapas	2016-06-30	2	34	120,159	1,402.95	4,187	97.70	99.19	-	-			-	-	yes
	MF801419	mex43	CLE	Serum	Mexico: Chiapas	2016-07-01	1	36	9,163	142.93	560	12.02	71.66	-	-			-	-	yes
	MF801420	mex44	CLE	Serum	Mexico: Chiapas	2016-07-04	4	33	3,187	51.55	364	7.09	60.40	557,586	3,855.77	123	17.55	58.67	66.90	yes
	MF801421	mex45	CLE	Serum	Mexico: Chiapas	2016-07-01	3	37	10,324	303.88	144	7.14	33.59	1,577,022	2,046.37	60	3.08	34.69	48.16	yes
	MF801422	mex46	CLE	Serum	Mexico: Guerrero	2016-07-01	1	38	4,138	104.43	164	5.40	45.48	1,635,805	2,533.24	125	2.77	62.17	67.82	yes
	MF801423	mex47	CLE	Serum	Mexico: Guerrero	2016-06-30	1	37	45,920	746.65	368	31.91	73.15	2,868,323	3,335.21	356	184.26	90.77	94.03	yes
	KY606273	mex48	CLE	Serum	Mexico: Guerrero	2016-06-30	1	31	1,130,251	7,144.51	12,716	484.93	99.29	-	-			-	-	yes
	NA	mex49	CLE	Serum	Mexico: Oaxaca	2016-06-30	2	39	2,666	222.57	48	15.43	9.07	530,355	3,670.94	22	2.69	8.05	12.91	no
	MF801424	mex50	CLE	Serum	Mexico: Yucatan	2016-07-01	3	36	3,573	163.68	102	4.53	22.39	900,457	3,305.70	60	2.73	26.85	32.71	yes

HCSFV: Health Center Sócrates Flores Vivas, Managua, Nicaragua
CDPH: CDPH California Department of Public Health, Richmond, CA, USA
BSRI: Blood System Research Institue, San Francisco, CA, USA
CLE: Central Laboratory of Epidemiology, Mexico City, Mexico

Table S2. Association between mosquito climatic vector suitability and Zika virus notified cases (related to Figure 3)

Country	Belize	Costa Rica	El Salvador	Guatemala	Honduras	Mexico	Nicaragua	Panama
Period	Jan 2016 - May 2017	Jan 2016 - May 2017	Oct 2015 - May 2017	Nov 2015 - May 2017	Jan 2016 - Mar 2017	Jan 2016 - Apr 2017	Jan 2016 - May 2017	Nov 2015 - Apr 2017
P-value	0.0352	0.000213	0.381	0.127	0.0656	2.24E-07	1.61E-06	0.438
Adjusted-R2	0.214	0.585	< 0	0.0806	0.179	0.851	0.78	< 0
Time lag (months)	1.06	0.81	5.91	-5.98	4.62	0.62	0.69	-2.58

For each country, the table provides the estimated correlated time period (*T*), *P*-value of the linear term of suitability in *T*, adjusted R^2 of the model, and time lag (*l*).

Table S3. 13-mer primers used in the metagenomic next-generation sequencing (related to Figure 1)

Primer Name	Primer Direction	Primer Sequence (without adapter)	Primer Sequence (with adapter)
ZIKV-13mer_251R	reverse	CTGCCATAGCTGT	GTTTCCCACTGGAGGATACTGCCATAGCTGT
ZIKV-13mer_501R	reverse	TTGTGATGGCAGG	GTTTCCCACTGGAGGATATTGTGATGGCAGG
ZIKV-13mer_751R	reverse	GTATGCCGGGGCA	GTTTCCCACTGGAGGATAGTATGCCGGGGCA
ZIKV-13mer_1001R	reverse	CAAGGTAGGCTTC	GTTTCCCACTGGAGGATACAAGGTAGGCTTC
ZIKV-13mer_1251R	reverse	TTCGCTCTATTCT	GTTTCCCACTGGAGGATATTCGCTCTATTCT
ZIKV-13mer_1501R	reverse	ATGTGCGTCCTTG	GTTTCCCACTGGAGGATAATGTGCGTCCTTG
ZIKV-13mer_1751R	reverse	CGTACTGTACCTC	GTTTCCCACTGGAGGATACGTACTGTACCTC
ZIKV-13mer_2001R	reverse	CTCACAGTGGCTT	GTTTCCCACTGGAGGATACTCACAGTGGCTT
ZIKV-13mer_2251R	reverse	GGATAAGAAGATC	GTTTCCCACTGGAGGATAGGATAAGAAGATC
ZIKV-13mer_2501R	reverse	GGATTGCGTTGAG	GTTTCCCACTGGAGGATAGGATTGCGTTGAG
ZIKV-13mer_2751R	reverse	ACCCCGAACCCAT	GTTTCCCACTGGAGGATAACCCCGAACCCAT
ZIKV-13mer_3001R	reverse	CTTGGGTATGATC	GTTTCCCACTGGAGGATACTTGGGTATGATC
ZIKV-13mer_3251R	reverse	AGCCATCTTTAGC	GTTTCCCACTGGAGGATAAGCCATCTTTAGC
ZIKV-13mer_3501R	reverse	AAAATTGCAAGCT	GTTTCCCACTGGAGGATAAAAATTGCAAGCT
ZIKV-13mer_3751R	reverse	AACCATCGCTCGT	GTTTCCCACTGGAGGATAAACCATCGCTCGT
ZIKV-13mer_4001R	reverse	TCCGCTTCCCACT	GTTTCCCACTGGAGGATATCCGCTTCCCACT
ZIKV-13mer_4251R	reverse	AAATCACCACTCT	GTTTCCCACTGGAGGATAAAATCACCACTCT
ZIKV-13mer_4501R	reverse	TCCAACTTGTGTT	GTTTCCCACTGGAGGATATCCAACTTGTGTT
ZIKV-13mer_4751R	reverse	CATCCTTTGTCTT	GTTTCCCACTGGAGGATACATCCTTTGTCTT
ZIKV-13mer_5001R	reverse	ACTCTCCTGGTTT	GTTTCCCACTGGAGGATAACTCTCCTGGTTT
ZIKV-13mer_5251R	reverse	GGCCTCATCCATA	GTTTCCCACTGGAGGATAGGCCTCATCCATA
ZIKV-13mer_5501R	reverse	CATTGCCGTTCCT	GTTTCCCACTGGAGGATACATTGCCGTTCCT
ZIKV-13mer_5751R	reverse	GCAGCGCTGGCAT	GTTTCCCACTGGAGGATAGCAGCGCTGGCAT
ZIKV-13mer_6001R	reverse	TTTCATGAGTTCC	GTTTCCCACTGGAGGATATTTCATGAGTTCC
ZIKV-13mer_6251R	reverse	AAGCCGCTCCTCT	GTTTCCCACTGGAGGATAAAGCCGCTCCTCT
ZIKV-13mer_6501R	reverse	GTCACCATTCCAA	GTTTCCCACTGGAGGATAGTCACCATTCCAA
ZIKV-13mer_6751R	reverse	TCTCCTTCCCATT	GTTTCCCACTGGAGGATATCTCCTTCCCATT
ZIKV-13mer_7001R	reverse	TTGTGAGTAGCAA	GTTTCCCACTGGAGGATATTGTGAGTAGCAA
ZIKV-13mer_7251R	reverse	GCGACAGTATGGC	GTTTCCCACTGGAGGATAGCGACAGTATGGC
ZIKV-13mer_7501R	reverse	ATCTGGTTCAAGC	GTTTCCCACTGGAGGATAATCTGGTTCAAGC
ZIKV-13mer_7751R	reverse	TCCTTTCACTTCT	GTTTCCCACTGGAGGATATCCTTTCACTTCT
ZIKV-13mer_8001R	reverse	GGCACAACACCTT	GTTTCCCACTGGAGGATAGGCACAACACCTT
ZIKV-13mer_8251R	reverse	CAGCTTACCACAG	GTTTCCCACTGGAGGATACAGCTTACCACAG
ZIKV-13mer_8501R	reverse	CTGACCATACGGT	GTTTCCCACTGGAGGATACTGACCATACGGT
ZIKV-13mer_8751R	reverse	CTAGAGCCCAGAA	GTTTCCCACTGGAGGATACTAGAGCCCAGAA
ZIKV-13mer_9001R	reverse	ACATATCCGAGTC	GTTTCCCACTGGAGGATAACATATCCGAGTC
ZIKV-13mer_9251R	reverse	CCCCCTTTGGTCT	GTTTCCCACTGGAGGATACCCCCTTTGGTCT
ZIKV-13mer_9501R	reverse	TATCATTCAAGAA	GTTTCCCACTGGAGGATATATCATTCAAGAA
ZIKV-13mer_9751R	reverse	AAATAAAGGAGCT	GTTTCCCACTGGAGGATAAAATAAAGGAGCT
ZIKV-13mer_10001R	reverse	GAGAGATCCACAC	GTTTCCCACTGGAGGATAGAGAGATCCACAC
ZIKV-13mer_10251R	reverse	TTGGTTTCCCAGC	GTTTCCCACTGGAGGATATTGGTTTCCCAGC
ZIKV-13mer_10501R	reverse	TGGTCTTTCCCAG	GTTTCCCACTGGAGGATATGGTCTTTCCCAG
ZIKV-13mer_10751R	reverse	GGATTTCCCCACA	GTTTCCCACTGGAGGATAGGATTTCCCCACA
ZIKV-13mer-3prime_7R	reverse	TCACAGCTAGTCT	GTTTCCCACTGGAGGATATCACAGCTAGTCT
ZIKV-13mer-3prime_6R	reverse	ATGCTGTTTTGCG	GTTTCCCACTGGAGGATAATGCTGTTTTGCG
ZIKV-13mer-3prime_5R	reverse	GGCGGCCAGCGTG	GTTTCCCACTGGAGGATAGGCGGCCAGCGTG
ZIKV-13mer-3prime_4R	reverse	GCGATCTGTGCCT	GTTTCCCACTGGAGGATAGCGATCTGTGCCT
ZIKV-13mer-3prime_3R	reverse	CCGCCGAAGTTCG	GTTTCCCACTGGAGGATACCGCCGAAGTTCG
ZIKV-13mer-3prime_1R	reverse	AGAAACCATGGAT	GTTTCCCACTGGAGGATAAGAAACCATGGAT
ZIKV-13mer-3prime_1R	reverse	AGAAACCATGGAT	GTTTCCCACTGGAGGATAAGAAACCATGGAT
ZIKV-13mer-3prime_3R	reverse	CCGCCGAAGTTCG	GTTTCCCACTGGAGGATACCGCCGAAGTTCG

Table S4. Bait capture probes used in the metagenomic next-generation sequencing (related to Figure 1)

SequenceName	Sequence
LC002520_Zika_virus_genomic_RNA,_complete_genome,_strain_MR766-NIID_1541	GCAAAGGGAGCTTGGTGACATGTGCCAAGTTTACGTGTTCTAAGAAGATGACCGGGAAGAGCATTCAACCGGAAAATCTGGAGTATCGGATAATGCTATCAGTGCATGGCTCCCAGCATA
gi|984874581|gb|KU501215.1| Zika virus strain PRVABC59, complete genome_1	GTTGTTGATCTGTGTGAATCAGACTGCGACAGTTCGAGTTTGAAGCGAAAGCTAGCAACAGTATCAACAGGTTTTATTTGGATTTGGAAACGAGAGTTTCTGGTCATGAAAAACCCAAA
gi|984874581|gb|KU501215.1| Zika virus strain PRVABC59, complete genome_111	AAAACCCAAAAAAGAAATCCGGAGGATTCCGGATTGTCAATATGCTAAAACGCGGAGTAGCCCGTGTGAGCCCCTTTGGGGGCTTGAAGAGGCTGCCAGCCGGACTTCTGCTGGGTCATG
gi|984874581|gb|KU501215.1| Zika virus strain PRVABC59, complete genome_221	CTGGGTCATGGGCCCATCAGGATGGTCTTGGCGATTCTAGCCTTTTTGAGATTCACGGCAATCAAGCCATCACTGGGTCTCATCAATAGATGGGGTTCAGTGGGGAAAAAAGAGGCTATG
gi|984874581|gb|KU501215.1| Zika virus strain PRVABC59, complete genome_331	AGAGGCTATGGAAACAATAAAGAAGTTCAAGAAAGATCTGGCTGCCATGCTGAGAATAATCAATGCTAGGAAGGAGAAGAAGAGACGAGGCGCAGATACTAGTGTCGGAATTGTTGGCCT
gi|984874581|gb|KU501215.1| Zika virus strain PRVABC59, complete genome_441	TTGTTGGCCTCCTGCTGACCACAGCTATGGCAGCGGAGGTCACTAGACGTGGGAGTGCATACTATATGTACTTGGACAGAAACGATGCTGGGGAGGCCATATCTTTTCCAACCACATTGG
gi|984874581|gb|KU501215.1| Zika virus strain PRVABC59, complete genome_551	ACCACATTGGGGATGAATAAGTGTTATATACAGATCATGGATCTTGGACACATGTGTGATGCCACCATGAGCTATGAATGCCCTATGCTGGATGAGGGGGTGGAACCAGATGACGTCGAT
gi|984874581|gb|KU501215.1| Zika virus strain PRVABC59, complete genome_661	TGACGTCGATTGTTGGTGCAACACGACGTCAACTTGGGTTGTGTACGGAACCTGCCATCACAAAAAAGGTGAAGCACGGAGATCTAGAAGAGCTGTGACGCTCCCCTCCCATTCCACCAG
gi|984874581|gb|KU501215.1| Zika virus strain PRVABC59, complete genome_771	ATTCCACCAGGAAGCTGCAAACGCGGTCGCAAACCTGGTTGGAATCAAGAGAATACACAAAGCACTTGATTAGAGTCGAAAATTGGATATTCAGGAACCCTGGCTTCGCGTTAGCAGCAG
gi|984874581|gb|KU501215.1| Zika virus strain PRVABC59, complete genome_881	TTAGCAGCAGCTGCCATCGCTTGGCTTTTGGGAAGCTCAACGAGCCAAAAAGTCATATACTTGGTCATGATACTGCTGATTGCCCCGGCATACAGCATCAGGTGCATAGGAGTCAGCAAT
gi|984874581|gb|KU501215.1| Zika virus strain PRVABC59, complete genome_991	AGTCAGCAATAGGGACTTTGTGGAAGGTATGTCAGGTGGGACTTGGGTTGATGTTGTCTTGGAACATGGAGGTTGTGTCACCGTAATGGCACAGGACAAACCGACTGTCGACATAGAGCT
gi|984874581|gb|KU501215.1| Zika virus strain PRVABC59, complete genome_1101	ACATAGAGCTGGTTACAACAACAGTCAGCAACATGGCGGAGGTAAGATCCTACTGCTATGAGGCATCAATATCAGACATGGCTTCTGACAGCCGCTGCCCAACACAAGGTGAAGCCTACC
gi|984874581|gb|KU501215.1| Zika virus strain PRVABC59, complete genome_1211	GAAGCCTACCTTGACAAGCAATCAGACACTCAATATGTCTGCAAAAGAACGTTAGTGGACAGAGGCTGGGGAAATGGATGTGGACTTTTTGGCAAAGGGAGCCTGGTGACATGCGCTAAG
gi|984874581|gb|KU501215.1| Zika virus strain PRVABC59, complete genome_1321	ATGCGCTAAGTTTGCATGCTCCAAGAAAATGACCGGGAAGAGCATCCAGCCAGAGAATCTGGAGTACCGGATAATGCTGTCAGTTCATGGCTCCCAGCACAGTGGGATGATCGTTAATGA
gi|984874581|gb|KU501215.1| Zika virus strain PRVABC59, complete genome_1431	TCGTTAATGACACAGGACATGAAACTGATGAGAATAGAGCGAAAGTTGAGATAACGCCCAATTCACCGAGAGCCGAAGCCACCCTGGGGGGTTTTGGAAGCCTAGGACTTGATTGTGAAC
gi|984874581|gb|KU501215.1| Zika virus strain PRVABC59, complete genome_1541	GATTGTGAACCGAGGACAGGCCTTGACTTTTCAGATTTGTATTACTTGACTATGAATAACAAGCACTGGTTGGTTCACAAGGAGTGGTTCCACGACATTCCATTACCTTGGCACGCTGGG
gi|984874581|gb|KU501215.1| Zika virus strain PRVABC59, complete genome_1651	GCACGCTGGGGCAGACACCGGAACTCCACACTGGAACAACAAAGAAGCACTGGTAGAGTTCAAGGACGCACATGCCAAAAGGCAAACTGTCGTGGTTCTAGGGAGTCAAGAAGGAGCAGT
gi|984874581|gb|KU501215.1| Zika virus strain PRVABC59, complete genome_1761	AAGGAGCAGTTCACACGGCCCTTGCTGGAGCTCTGGAGGCTGAGATGGATGGTGCAAAGGGAAGGCTGTCCTCTGGCCACTTGAAATGTCGCCTGAAAATGGATAAACTTAGATTGAAGG
gi|984874581|gb|KU501215.1| Zika virus strain PRVABC59, complete genome_1871	AGATTGAAGGGCGTGTCATACTCCTTGTGTACTGCAGCGTTCACATTCACCAAGATCCCGGCTGAAACACTGCACGGGACAGTCACAGTGGAGGTACAGTACGCAGGGACAGATGGACCT
gi|984874581|gb|KU501215.1| Zika virus strain PRVABC59, complete genome_1981	AGATGGACCTTGCAAGGTTCCAGCTCAGATGGCGGTGGACATGCAAACTCTGACCCCAGTTGGGAGGTTGATAACCGCTAACCCCGTAATCACTGAAAGCACTGAGAACTCTAAGATGAT
gi|984874581|gb|KU501215.1| Zika virus strain PRVABC59, complete genome_2091	CTAAGATGATGCTGGAACTTGATCCACCATTTGGGGACTCTTACATTGTCATAGGAGTCGGGGAGAAGAAGATCACCCACCACTGGCACAGGAGTGGCAGCACCATTGGAAAAGCATTTG
gi|984874581|gb|KU501215.1| Zika virus strain PRVABC59, complete genome_2201	AAAGCATTTGAAGCCACTGTGAGAGGTGCCAAGAGAATGGCAGTCTTGGGAGACACAGCCTGGGACTTTGGATCAGTTGGAGGCGCTCTCAACTCATTGGGCAAGGGCATCCATCAAATT
gi|984874581|gb|KU501215.1| Zika virus strain PRVABC59, complete genome_2311	CCATCAAATTTTTGGAGCAGCTTTCAAATCATTGTTTGGAGGAATGTCCTGGTTCTCACAAATTCTCATTGGAACGTTGCTGATGTGGTTGGGTCTGAACACAAAGAATGGATCTATTTC
gi|984874581|gb|KU501215.1| Zika virus strain PRVABC59, complete genome_2421	GATCTATTTCCCTTATGTGCTTGGCCTTAGGGGGAGTGTTGATCTTCTTATCCACAGCCGTCTCTGCTGATGTGGGGTGCTCGGTGGACTTCTCAAAGAAGGAGACGAGATGCGGTACAG
gi|984874581|gb|KU501215.1| Zika virus strain PRVABC59, complete genome_2531	TGCGGTACAGGGGTGTTCGTCTATAACGACGTTGAAGCCTGGAGGGACAGGTACAAGTACCATCCTGACTCCCCCCGTAGATTGGCAGCAGCAGTCAAGCAAGCCTGGGAAGATGGTATC
gi|984874581|gb|KU501215.1| Zika virus strain PRVABC59, complete genome_2641	AGATGGTATCTGCGGGATCTCCTCTGTTTCAAGAATGGAAAACATCATGTGGAGATCAGTAGAAGGGGAGCTCAACGCAATCCTGGAAGAGAATGGAGTTCAACTGACGGTCGTTGTGGG
gi|984874581|gb|KU501215.1| Zika virus strain PRVABC59, complete genome_2751	TCGTTGTGGGATCTGTAAAAAACCCCATGTGGAGAGGTCCACAGAGATTGCCCGTGCCTGTGAACGAGCTGCCCCACGGCTGGAAGGCTTGGGGGAAATCGTATTTCGTCAGAGCAGCAA
gi|984874581|gb|KU501215.1| Zika virus strain PRVABC59, complete genome_2861	AGAGCAGCAAAGACAAATAACAGCTTTGTCGTGGATGGTGACACACTGAAGGAATGCCCACTCAAACATAGAGCATGGAACAGCTTTCTTGTGGAGGATCATGGGTTCGGGGTATTTCAC
gi|984874581|gb|KU501215.1| Zika virus strain PRVABC59, complete genome_2971	GGTATTTCACACTAGTGTCTGGCTCAAGGTTAGAGAAGATTATTCATTAGAGTGTGATCCAGCCGTTATTGGAACAGCTGTTAAGGGAAAGGAGGCTGTACACAGTGATCTAGGCTACTG
gi|984874581|gb|KU501215.1| Zika virus strain PRVABC59, complete genome_3081	TAGGCTACTGGATTGAGAGTGAGAAGAATGACACATGGAGGCTGAAGAGGGCCCATCTGATCGAGATGAAAACATGTGAATGGCCAAAGTCCCACACATTGTGGACAGATGGAATAGAAG
gi|984874581|gb|KU501215.1| Zika virus strain PRVABC59, complete genome_3191	GGAATAGAAGAGAGTGATCTGATCATACCCAAGTCTTTAGCTGGGCCACTCAGCCATCACAATACCAGAGAGGGCTACAGGACCCAAATGAAAGGGCCATGGCACAGTGAAGAGCTTGAA
gi|984874581|gb|KU501215.1| Zika virus strain PRVABC59, complete genome_3301	AGAGCTTGAAATTCGGTTTGAGGAATGCCCAGGCACTAAGGTCCACGTGGAGGAAACATGTGGAACAAGAGGACCATCTCTGAGATCAACCACTGCAAGCGGAAGGGTGATCGAGGAATG
gi|984874581|gb|KU501215.1| Zika virus strain PRVABC59, complete genome_3411	TCGAGGAATGGTGCTGCAGGGAGTGCACAATGCCCCCACTGTCGTTCCGGGCTAAAGATGGCTGTTGGTATGGAATGGAGATAAGGCCCAGGAAAGAACCAGAAAGCAACTTAGTAAGGT
gi|984874581|gb|KU501215.1| Zika virus strain PRVABC59, complete genome_3521	TTAGTAAGGTCAATGGTGACTGCAGGATCAACTGATCACATGGACCACTTCTCCCTTGGAGTGCTTGTGATCCTGCTCATGGTGCAGGAAGGGCTGAAGAAGAGAATGACCACAAAGATC
gi|984874581|gb|KU501215.1| Zika virus strain PRVABC59, complete genome_3631	CACAAAGATCATCATAAGCACATCAATGGCAGTGCTGGTAGCTATGATCCTGGGAGGATTTTCAATGAGTGACCTGGCTAAGCTTGCAATTTTGATGGGTGCCACCTTCGCGGAAATGAA
gi|984874581|gb|KU501215.1| Zika virus strain PRVABC59, complete genome_3741	CGGAAATGAACACTGGAGGAGATGTAGCTCATCTGGCGCTGATAGCGGCATTCAAAGTCAGACCAGCGTTGCTGGTATCTTTCATCTTCAGAGCTAATTGGACACCCCGTGAAAGCATGC
gi|984874581|gb|KU501215.1| Zika virus strain PRVABC59, complete genome_3851	GAAAGCATGCTGCTGGCCTTGGCCTCGTGTCTTTTGCAAACTGCGATCTCCGCCTTGGAAGGCGACCTGATGGTTCTCATCAATGGTTTTGCTTTGGCCTGGTTGGCAATACGAGCGATG
gi|984874581|gb|KU501215.1| Zika virus strain PRVABC59, complete genome_3961	ACGAGCGATGGTTGTTCCACGCACTGATAACATCACCTTGGCAATCCTGGCTGCTCTGACACCACTGGCCCGGGGCACACTGCTTGTGGCGTGGAGAGCAGGCCTTGCTACTTGCGGGGG
gi|984874581|gb|KU501215.1| Zika virus strain PRVABC59, complete genome_4071	CTTGCGGGGGGTTTATGCTCCTCTCTCTGAAGGGAAAAGGCAGTGTGAAGAAGAACTTACCATTTGTCATGGCCCTGGGACTAACCGCTGTGAGGCTGGTCGACCCCATCAACGTGGTGG
gi|984874581|gb|KU501215.1| Zika virus strain PRVABC59, complete genome_4181	AACGTGGTGGGACTGCTGTTGCTCACAAGGAGTGGGAAGCGGAGCTGGCCCCCTAGCGAAGTACTCACAGCTGTTGGCCTGATATGCGCATTGGCTGGAGGGTTCGCCAAGGCAGATATA
gi|984874581|gb|KU501215.1| Zika virus strain PRVABC59, complete genome_4291	GGCAGATATAGAGATGGCTGGGCCCATGGCCGCGGTCGGTCTGCTAATTGTCAGTTACGTGGTCTCAGGAAAGAGTGTGGACATGTACATTGAAAGAGCAGGTGACATCACATGGGAAAA
gi|984874581|gb|KU501215.1| Zika virus strain PRVABC59, complete genome_4401	CATGGGAAAAAGATGCGGAAGTCACTGGAAACAGTCCCCGGCTCGATGTGGCGCTAGATGAGAGTGGTGATTTCTCCCTGGTGGAGGATGACGGTCCCCCCATGAGAGAGATCATACTCA
gi|984874581|gb|KU501215.1| Zika virus strain PRVABC59, complete genome_4511	ATCATACTCAAGGTGGTCCTGATGACCATCTGTGGCATGAACCCAATAGCCATACCCTTTGCAGCTGGAGCGTGGTACGTATACGTGAAGACTGGAAAAAGGAGTGGTGCTCTATGGGAT
gi|984874581|gb|KU501215.1| Zika virus strain PRVABC59, complete genome_4621	TCTATGGGATGTGCCTGCTCCCAAGGAAGTAAAAAAGGGGGAGACCACAGATGGAGTGTACAGAGTAATGACTCGTAGACTGCTAGGTTCAACACAAGTTGGAGTGGGAGTTATGCAAGA
gi|984874581|gb|KU501215.1| Zika virus strain PRVABC59, complete genome_4731	TTATGCAAGAGGGGGTCTTTCACACTATGTGGCACGTCACAAAAGGATCCGCGCTGAGAAGCGGTGAAGGGAGACTTGATCCATACTGGGGAGATGTCAAGCAGGATCTGGTGTCATACT
gi|984874581|gb|KU501215.1| Zika virus strain PRVABC59, complete genome_4841	GTGTCATACTGTGGTCCATGGAAGCTAGATGCCGCCTGGGATGGGCACAGCGAGGTGCAGCTCTTGGCCGTGCCCCCCGGAGAGAGAGCGAGGAACATCCAGACTCTGCCCGGAATATTT
gi|984874581|gb|KU501215.1| Zika virus strain PRVABC59, complete genome_4951	CGGAATATTTAAGACAAAGGATGGGGACATTGGAGCGGTTGCGCTGGATTACCCAGCAGGAACTTCAGGATCTCCAATCCTAGACAAGTGTGGGAGAGTGATAGGACTTTATGGCAATGG
gi|984874581|gb|KU501215.1| Zika virus strain PRVABC59, complete genome_5061	ATGGCAATGGGGTCGTGATCAAAAACGGGAGTTATGTTAGTGCCATCACCCAAGGGAGGAGGGAGGAAGAGACTCCTGTTGAGTGCTTCGAGCCCTCGATGCTGAAGAAGAAGCAGCTAA
gi|984874581|gb|KU501215.1| Zika virus strain PRVABC59, complete genome_5171	AAGCAGCTAACTGTCTTAGACTTGCATCCTGGAGCTGGGAAAACCAGGAGAGTTCTTCCTGAAATAGTCCGTGAAGCCATAAAAACAAGACTCCGTACTGTGATCTTAGCTCCAACCAGG
gi|984874581|gb|KU501215.1| Zika virus strain PRVABC59, complete genome_5281	TCCAACCAGGGTTGTCGCTGCTGAAATGGAGGAGGCCCTTAGAGGGCTTCCAGTGCGTTATATGACAACAGCAGTCAATGTCACCCACTCTGGAACAGAAATCGTCGACTTAATGTGCCA
gi|984874581|gb|KU501215.1| Zika virus strain PRVABC59, complete genome_5391	TAATGTGCCATGCCACCTTCACTTCACGTCTACTACAGCCAATCAGAGTCCCCAACTATAATCTGTATATTATGGATGAGGCCCACTTCACAGATCCCTCAAGTATAGCAGCAAGAGGAT
gi|984874581|gb|KU501215.1| Zika virus strain PRVABC59, complete genome_5501	GCAAGAGGATACATTTCAACAAGGGTTGAGATGGGCGAGGCGGCTGCCATCTTCATGACCGCCACGCCACCAGGAACCCGTGACGCATTTCCGGACTCCAACTCACCAATTATGGACACC
gi|984874581|gb|KU501215.1| Zika virus strain PRVABC59, complete genome_5611	TATGGACACCGAAGTGGAAGTCCCAGAGAGAGCCTGGAGCTCAGGCTTTGATTGGGTGACGGATCATTCTGGAAAAACAGTTTGGTTTGTTCCAAGCGTGAGGAACGGCAATGAGATCGC
gi|984874581|gb|KU501215.1| Zika virus strain PRVABC59, complete genome_5721	ATGAGATCGCAGCTTGTCTGACAAAGGCTGGAAAACGGGTCATACAGCTCAGCAGAAAGACTTTTGAGACAGAGTTCCAGAAAACAAAACATCAAGAGTGGGACTTTGTCGTGACAACTG
gi|984874581|gb|KU501215.1| Zika virus strain PRVABC59, complete genome_5831	GTGACAACTGACATTTCAGAGATGGGCGCCAACTTTAAAGCTGACCGTGTCATAGATTCCAGGAGATGCCTAAAGCCGGTCATACTTGATGGCGAGAGAGTCATTCTGGCTGGACCCATG
gi|984874581|gb|KU501215.1| Zika virus strain PRVABC59, complete genome_5941	TGGACCCATGCCTGTCACACATGCCAGCGCTGCCCAGAGGAGGGGGCGCATAGGCAGGAATCCCAACAAACCTGGAGATGAGTATCTGTATGGAGGTGGGTGCGCAGAGACTGACGAAGA
gi|984874581|gb|KU501215.1| Zika virus strain PRVABC59, complete genome_6051	CTGACGAAGACCATGCACACTGGCTTGAAGCAAGAATGCTCCTTGACAATATTTACCTCCAAGATGGCCTCATAGCCTCGCTCTATCGACCTGAGGCCGACAAAGTAGCAGCCATTGAGG
gi|984874581|gb|KU501215.1| Zika virus strain PRVABC59, complete genome_6161	GCCATTGAGGGAGAGTTCAAGCTTAGGACGGAGCAAAGGAAGACCTTTGTGGAACTCATGAAAAGAGGAGATCTTCCTGTTTGGCTGGCCTATCAGGTTGCATCTGCCGGAATAACCTAC
gi|984874581|gb|KU501215.1| Zika virus strain PRVABC59, complete genome_6271	AATAACCTACACAGATAGAAGATGGTGCTTTGATGGCACGACCAACAACACCATAATGGAAGACAGTGTGCCGGCAGAGGTGTGGACCAGACACGGAGAGAAAAGAGTGCTCAAACCGAG
gi|984874581|gb|KU501215.1| Zika virus strain PRVABC59, complete genome_6381	TCAAACCGAGGTGGATGGACGCCAGAGTTTGTTCAGATCATGCGGCCCTGAAGTCATTCAAGGAGTTTGCCGCTGGGAAAAGAGGAGCGGCTTTTGGAGTGATGGAAGCCCTGGGAACAC
gi|984874581|gb|KU501215.1| Zika virus strain PRVABC59, complete genome_6491	CTGGGAACACTGCCAGGACACATGACAGAGAGATTCCAGGAAGCCATTGACAACCTCGCTGTGCTCATGCGGGCAGAGACTGGAAGCAGGCCTTACAAAGCCGCGGCGGCCCAATTGCCG
gi|984874581|gb|KU501215.1| Zika virus strain PRVABC59, complete genome_6601	CCAATTGCCGGAGACCCTAGAGACCATAATGCTTTTGGGGTTGCTGGGAACAGTCTCGCTGGGAATCTTCTTCGTCTTGATGAGGAACAAGGGCATAGGGAAGATGGGCTTTGGAATGGT
gi|984874581|gb|KU501215.1| Zika virus strain PRVABC59, complete genome_6711	TTGGAATGGTGACTCTTGGGGCCAGCGCATGGCTCATGTGGCTCTCGGAAATTGAGCCAGCCAGAATTGCATGTGTCCTCATTGTTGTGTTCCTATTGCTGGTGGTGCTCATACCTGAGC
gi|984874581|gb|KU501215.1| Zika virus strain PRVABC59, complete genome_6821	ATACCTGAGCCAGAAAAGCAAAGATCTCCCCAGGACAACCAAATGGCAATCATCATCATGGTAGCAGTAGGTCTTCTGGGCTTGATTACCGCCAATGAACTCGGATGGTTGGAGAGAACA
gi|984874581|gb|KU501215.1| Zika virus strain PRVABC59, complete genome_6931	GGAGAGAACAAAGAGTGACCTAAGCCATCTAATGGGAAGGAGAGAGGAGGGGGCAACCATAGGATTCTCAATGGACATTGACCTGCGGCCAGCCTCAGCTTGGGCCATCTATGCTGCCTT
gi|984874581|gb|KU501215.1| Zika virus strain PRVABC59, complete genome_7041	ATGCTGCCTTGACAACTTTCATTACCCCAGCCGTCCAACATGCAGTGACCACCTCATACAACAACTACTCCTTAATGGCGATGGCCACGCAAGCTGGAGTGTTGTTTGGCATGGGCAAAG
gi|984874581|gb|KU501215.1| Zika virus strain PRVABC59, complete genome_7151	ATGGGCAAAGGGATGCCATTCTACGCATGGGACTTTGGAGTCCCGCTGCTAATGATAGGTTGCTACTCACAATTAACACCCCTGACCCTAATAGTGGCCATCATTTTGCTCGTGGCGCAC
gi|984874581|gb|KU501215.1| Zika virus strain PRVABC59, complete genome_7261	CGTGGCGCACTACATGTACTTGATCCCAGGGCTGCAGGCAGCAGCTGCGCGTGCTGCCCAGAAGAGAACGGCAGCTGGCATCATGAAGAACCCTGTTGTGGATGGAATAGTGGTGACTGA
gi|984874581|gb|KU501215.1| Zika virus strain PRVABC59, complete genome_7371	TGGTGACTGACATTGACACAATGACAATTGACCCCCAAGTGGAGAAAAAGATGGGACAGGTGCTACTCATAGCAGTAGCCGTCTCCAGCGCCATACTGTCGCGGACCGCCTGGGGGTGGG
gi|984874581|gb|KU501215.1| Zika virus strain PRVABC59, complete genome_7481	TGGGGGTGGGGGGAGGCTGGGGCTCTGATCACAGCCGCAACTTCCACTTTGTGGGAAGGCTCTCCGAACAAGTACTGGAACTCCTCTACAGCCACTTCACTGTGTAACATTTTTAGGGGA
gi|984874581|gb|KU501215.1| Zika virus strain PRVABC59, complete genome_7591	TTTTAGGGGAAGTTACTTGGCTGGAGCTTCTCTAATCTACACAGTAACAAGAAACGCTGGCTTGGTCAAGAGACGTGGGGGTGGAACAGGAGAGACCCTGGGAGAGAAATGGAAGGCCCG
gi|984874581|gb|KU501215.1| Zika virus strain PRVABC59, complete genome_7701	GGAAGGCCCGCTTGAACCAGATGTCGGCCCTGGAGTTCTACTCCTACAAAAAGTCAGGCATCACCGAGGTGTGCAGAGAAGAGGCCCGCCGCGCCCTCAAGGACGGTGTGGCAACGGGAG
gi|984874581|gb|KU501215.1| Zika virus strain PRVABC59, complete genome_7811	GCAACGGGAGGCCATGCTGTGTCCCGAGGAAGTGCAAAGCTGAGATGGTTGGTGGAGCGGGGATACCTGCAGCCCTATGGAAAGGTCATTGATCTTGGATGTGGCAGAGGGGGCTGGAGT
gi|984874581|gb|KU501215.1| Zika virus strain PRVABC59, complete genome_7921	GGGCTGGAGTTACTACGTCGCCACCATCCGCAAAGTTCAAGAAGTGAAAGGATACACAAAAGGAGGCCCTGGTCATGAAGAACCCGTGTTGGTGCAAAGCTATGGGTGGAACATAGTCCG
gi|984874581|gb|KU501215.1| Zika virus strain PRVABC59, complete genome_8031	ACATAGTCCGTCTTAAGAGTGGGGTGGACGTCTTTCATATGGCGGCTGAGCCGTGTGACACGTTGCTGTGTGACATAGGTGAGTCATCATCTAGTCCTGAAGTGGAAGAAGCACGGACGC
gi|984874581|gb|KU501215.1| Zika virus strain PRVABC59, complete genome_8141	GCACGGACGCTCAGAGTCCTCTCCATGGTGGGGGATTGGCTTGAAAAAAGACCAGGAGCCTTTTGTATAAAAGTGTTGTGCCCATACACCAGCACTATGATGGAAACCCTGGAGCGACTG
gi|984874581|gb|KU501215.1| Zika virus strain PRVABC59, complete genome_8251	GGAGCGACTGCAGCGTAGGTATGGGGGAGGACTGGTCAGAGTGCCACTCTCCCGCAACTCTACACATGAGATGTACTGGGTCTCTGGAGCGAAAAGCAACACCATAAAAAGTGTGTCCAC
gi|984874581|gb|KU501215.1| Zika virus strain PRVABC59, complete genome_8361	GTGTGTCCACCACGAGCCAGCTCCTCTTGGGGCGCATGGACGGGCCTAGGAGGCCAGTGAAATATGAGGAGGATGTGAATCTCGGCTCTGGCACGCGGGCTGTGGTAAGCTGCGCTGAAG
gi|984874581|gb|KU501215.1| Zika virus strain PRVABC59, complete genome_8471	TGCGCTGAAGCTCCCAACATGAAGATCATTGGTAACCGCATTGAAAGGATCCGCAGTGAGCACGCGGAAACGTGGTTCTTTGACGAGAACCACCCATATAGGACATGGGCTTACCATGGA
gi|984874581|gb|KU501215.1| Zika virus strain PRVABC59, complete genome_8581	TTACCATGGAAGCTATGAGGCCCCCACACAAGGGTCAGCGTCCTCTCTAATAAACGGGGTTGTCAGGCTCCTGTCAAAACCCTGGGATGTGGTGACTGGAGTCACAGGAATAGCCATGAC
gi|984874581|gb|KU501215.1| Zika virus strain PRVABC59, complete genome_8691	TAGCCATGACCGACACCACACCGTATGGTCAGCAAAGAGTTTTCAAGGAAAAAGTGGACACTAGGGTGCCAGACCCCCAAGAAGGCACTCGTCAGGTTATGAGCATGGTCTCTTCCTGGT
gi|984874581|gb|KU501215.1| Zika virus strain PRVABC59, complete genome_8801	TCTTCCTGGTTGTGGAAAGAGCTAGGCAAACACAAACGGCCACGAGTCTGCACCAAAGAAGAGTTCATCAACAAGGTTCGTAGCAATGCAGCATTAGGGGCAATATTTGAAGAGGAAAAA
gi|984874581|gb|KU501215.1| Zika virus strain PRVABC59, complete genome_8911	AGAGGAAAAAGAGTGGAAGACTGCAGTGGAAGCTGTGAACGATCCAAGGTTCTGGGCTCTAGTGGACAAGGAAAGAGAGCACCACCTGAGAGGAGAGTGCCAGAGCTGTGTGTACAACAT
gi|984874581|gb|KU501215.1| Zika virus strain PRVABC59, complete genome_9021	TGTACAACATGATGGGAAAAAGAGAAAAGAAACAAGGGGAATTTGGAAAGGCCAAGGGCAGCCGCGCCATCTGGTATATGTGGCTAGGGGCTAGATTTCTAGAGTTCGAAGCCCTTGGAT
gi|984874581|gb|KU501215.1| Zika virus strain PRVABC59, complete genome_9131	GCCCTTGGATTCTTGAACGAGGATCACTGGATGGGGAGAGAGAACTCAGGAGGTGGTGTTGAAGGGCTGGGATTACAAAGACTCGGATATGTCCTAGAAGAGATGAGTCGTATACCAGGA
gi|984874581|gb|KU501215.1| Zika virus strain PRVABC59, complete genome_9241	TATACCAGGAGGAAGGATGTATGCAGATGACACTGCTGGCTGGGACACCCGCATTAGCAGGTTTGATCTGGAGAATGAAGCTCTAATCACCAACCAAATGGAGAAAGGGCACAGGGCCTT
gi|984874581|gb|KU501215.1| Zika virus strain PRVABC59, complete genome_9351	ACAGGGCCTTGGCATTGGCCATAATCAAGTACACATACCAAAACAAAGTGGTAAAGGTCCTTAGACCAGCTGAAAAAGGGAAAACAGTTATGGACATTATTTCGAGACAAGACCAAAGGG
gi|984874581|gb|KU501215.1| Zika virus strain PRVABC59, complete genome_9461	GACCAAAGGGGGAGCGGACAAGTTGTCACTTACGCTCTTAACACATTTACCAACCTAGTGGTGCAACTCATTCGGAATATGGAGGCTGAGGAAGTTCTAGAGATGCAAGACTTGTGGCTG
gi|984874581|gb|KU501215.1| Zika virus strain PRVABC59, complete genome_9571	CTTGTGGCTGCTGCGGAGGTCAGAGAAAGTGACCAACTGGTTGCAGAGCAACGGATGGGATAGGCTCAAACGAATGGCAGTCAGTGGAGATGATTGCGTTGTGAAGCCAATTGATGATAG
gi|984874581|gb|KU501215.1| Zika virus strain PRVABC59, complete genome_9681	TTGATGATAGGTTTGCACATGCCCTCAGGTTCTTGAATGATATGGGAAAAGTTAGGAAGGACACACAAGAGTGGAAACCCTCAACTGGATGGGACAACTGGGAAGAAGTTCCGTTTTGCT
gi|984874581|gb|KU501215.1| Zika virus strain PRVABC59, complete genome_9791	CCGTTTTGCTCCCACCACTTCAACAAGCTCCATCTCAAGGACGGGAGGTCCATTGTGGTTCCCTGCCGCCACCAAGATGAACTGATTGGCCGGGCCCGCGTCTCTCCAGGGGCGGGATGG
gi|984874581|gb|KU501215.1| Zika virus strain PRVABC59, complete genome_9901	GGCGGGATGGAGCATCCGGGAGACTGCTTGCCTAGCAAAATCATATGCGCAAATGTGGCAGCTCCTTTATTTCCACAGAAGGGACCTCCGACTGATGGCCAATGCCATTTGTTCATCTGT
gi|984874581|gb|KU501215.1| Zika virus strain PRVABC59, complete genome_10011	GTTCATCTGTGCCAGTTGACTGGGTTCCAACTGGGAGAACTACCTGGTCAATCCATGGAAAGGGAGAATGGATGACCACTGAAGACATGCTTGTGGTGTGGAACAGAGTGTGGATTGAGG
gi|984874581|gb|KU501215.1| Zika virus strain PRVABC59, complete genome_10121	TGGATTGAGGAGAACGACCACATGGAAGACAAGACCCCAGTTACGAAATGGACAGACATTCCCTATTTGGGAAAAAGGGAAGACTTGTGGTGTGGATCTCTCATAGGGCACAGACCGCGC
gi|984874581|gb|KU501215.1| Zika virus strain PRVABC59, complete genome_10231	CAGACCGCGCACCACCTGGGCTGAGAACATTAAAAACACAGTCAACATGGTGCGCAGGATCATAGGTGATGAAGAAAAGTACATGGACTACCTATCCACCCAAGTTCGCTACTTGGGTGA
gi|984874581|gb|KU501215.1| Zika virus strain PRVABC59, complete genome_10341	ACTTGGGTGAAGAAGGGTCTACACCTGGAGTGCTGTAAGCACCAATCTTAATGTTGTCAGGCCTGCTAGTCAGCCACAGCTTGGGGAAAGCTGTGCAGCCTGTGACCCCCCCAGGAGAAG
gi|984874581|gb|KU501215.1| Zika virus strain PRVABC59, complete genome_10451	CCAGGAGAAGCTGGGAAACCAAGCCTATAGTCAGGCCGAGAACGCCATGGCACGGAAGAAGCCATGCTGCCTGTGAGCCCCTCAGAGGACACTGAGTCAAAAAACCCCACGCGCTTGGAG
gi|984874581|gb|KU501215.1| Zika virus strain PRVABC59, complete genome_10561	GCGCTTGGAGGCGCAGGATGGGAAAAGAAGGTGGCGACCTTCCCCACCCTTCAATCTGGGGCCTGAACTGGAGATCAGCTGTGGATCTCCAGAAGAGGGACTAGTGGTTAGAGGA
LC002520_Zika_virus_genomic_RNA,_complete_genome,_strain	ACACAGGTTGGAGTGGGAGTCATGCAAGAGGGAGTCTTCCACACCATGTGGCACGTTACAAAAGGAGCCGCACTGAGGAGCGGTGAGGGAAGACTTGATCCATACTGGGGGGATGTCAAG
LC002520_Zika_virus_genomic_RNA,_complete_genome,_strain	GGATGTCAAGCAGGACTTGGTGTCATACTGTGGGCCTTGGAAGTTGGATGCAGCTTGGGATGGACTCAGCGAGGTACAGCTTTTGGCCGTACCTCCCGGAGAGAGAGGGCCAGAAACATTCA
LC002520_Zika_virus_genomic_RNA,_complete_genome,_strain	GAAACATTCAGACCCTGCCTGGAATATTCAAGACAAAGGACGGGGACATCGGAGCAGTTGCTCTGGACTACCCTGCAGGGACCTCAGGATCTCCGATCCTAGACAAATGTGGAAGAGTGA
LC002520_Zika_virus_genomic_RNA,_complete_genome,_strain	GGAAGAGTGATAGGACTCTATGGCAATGGGGTTGTGATCAAGAATGGAAGCTATGTTAGTGCTATAACCCAGGGAAAGAGGGAGGAGGAGACTCCGGTTGAATGTTTCGAACCCTCGATG
LC002520_Zika_virus_genomic_RNA,_complete_genome,_strain	ACCCTCGATGCTGAAGAAGAAGCAGCTAACTGTCTTGGATCTGCATCCAGGAGCCGGAAAAACCAGGAGAGTTCTTCCTGAAATAGTCCGTGAAGCCATAAAAAAGAGACTCCGGACAGT
LC002520_Zika_virus_genomic_RNA,_complete_genome,_strain	GTGGATCCCTTATAGGGCACAGACCCCGCACCACTTGGGCTGAAAACATCAAAGACACAGTCAACATGGTGCGCAGGATCATAGGTGATGAAGAAAAGTACATGGACTATCTATCCACCC
LC002520_Zika_virus_genomic_RNA,_complete_genome,_strain	CTATCCACCCAAGTCCGCTACTTGGGTGAGGAAGGGTCCACACCCGGAGTGTTGTAAGCACCAATTTTAGTGTTGTCAGGCCTGCTAGTCAGCCACAGTTTGGGGAAAGCTGTGCAGCCT
LC002520_Zika_virus_genomic_RNA,_complete_genome,_strain	TGTGCAGCCTGTAACCCCCCCAGGAGAAGCTGGGAAACCAAGCTCATAGTCAGGCCGAGAACGCCATGGCACGGAAGAAGCCATGCTGCCTGTGAGCCCCTCAGAGGACACTGAGTCAAA
LC002520_Zika_virus_genomic_RNA,_complete_genome,_strain	CTGAGTCAAAAAACCCCACGCGCTTGGAAGCGCAGGATGGGAAAAGAAGGTGGCGACCTTCCCCACCCTTCAATCTGGGGCCTGAACTGGAGACTAGCTGTGAATCTCCAGCAGAGGGAC
LC002520_Zika_virus_genomic_RNA,_complete_genome,_strain	GCAGAGGGACTAGTGGTTAGAGGAGACCCCCCGGAAAACGCAAAACAGCATATTGACGCTGGGAAAGACCAGAGACTCCATGAGTTTCCACCACGCTGGCCGCCAGGCACAGATCGCCGA
DQ859059_Zika_virus_strain_MR_766_polyprotein_gene,_complete_cds_8801	CCTACCACGGGAGCTACGAAGCCCCCACGCAAGGGTCAGCATCTTCCCTCGTGAATGGGGTTGTTAGACTCCTGTCAAAGCCCTGGGATGTGGTGACTGGAGTTACAGGAATAGCTATGA
DQ859059_Zika_virus_strain_MR_766_polyprotein_gene,_complete_cds_9241	GTGTACAACATGATGGGAAAAAGAGAAAAGAAGCAAGGGGAATTCGGGAAAGCAAAAGGCAGTCGCGCCATCTGGTACATGTGGTTGGGAGCCAGATTCTTGGAGTTTGAAGCCCTTGGA
HQ234499_Zika_virus_isolate_P6-740_polyprotein_gene,_partial_cds_3631	ACTGCAAGCGGAAGGGTGATCGAGGAATGGTGCTGCAGGGAATGCACAATGCCCCCATTGTCGTTCCGGGCAAAAGATGGCTGTTGGTATGGAATGGAGATAAGGCCCAGGAAGGAACCA
HQ234499_Zika_virus_isolate_P6-740_polyprotein_gene,_partial_cds_3741	GAAGGAACCAGAGAGTAACCTAGTAAGGTCAATGGTGACTGCAGGATCAACTGATCACATGGATCACTTCTCCCTTGGAGTGCTTGTGATTCTGCTCATGGTGCAGGAAGGGCTGAAGAA
HQ234499_Zika_virus_isolate_P6-740_polyprotein_gene,_partial_cds_3851	GGCTGAAGAAGAGAATGACCACAAAGATCATCATAAGCACATCAATGGCAGTGTTGGTAGCTATGATCCTGGGAGGATTTTCAATGAGTGACTTGGCTAAGCTTGCAATTCTGATGGGTG
HQ234499_Zika_virus_isolate_P6-740_polyprotein_gene,_partial_cds_3961	CTGATGGGTGCCACCTTCGCGGAAATGAACACTGGAGGAGATGTAGCTCATCTGGCGCTGATAGCGGCATTCAAAGTCAGACCCGCGTTGCTGGTCTCTTTCATCTTCAGAGCCAATTGG
HQ234499_Zika_virus_isolate_P6-740_polyprotein_gene,_partial_cds_4071	AGCCAATTGGACACCCCGTGAGAGCATGCTGCTGGCCTTGGCCTCGTGCCTTCTGCAAACTGNGATCTCCGCCCTGGAAGGCGACCTGATGGTTCTCATCAATGGTTTTGCTTTGGCCTG
HQ234499_Zika_virus_isolate_P6-740_polyprotein_gene,_partial_cds_9131	CACTGGGGGCAATATTTGAAGAGGAGAAAGAATGGAAGACTGCAGTGGAAGCTGTGAACGATCCAAGGTTCTGGGCCCTAGTGGACAAGGAAAGAGAGCACCACTTGAGAGGAGAGTGTC
HQ234499_Zika_virus_isolate_P6-740_polyprotein_gene,_partial_cds_9241	GGAGAGTGTCAGAGCTGTGTGTACAACATGATGGGAAAAAGAGAAAAGAAGCAAGGGGAATTTGGAAAGGCCAAGGGCAGCCGCGCCATTTGGTACATGTGGCTAGGGGCTAGATTTCTA
HQ234499_Zika_virus_isolate_P6-740_polyprotein_gene,_partial_cds_9351	TAGATTTCTAGAGTTTGAAGCCCTTGGATTCTTGAACGAGGATCACTGGATGGGGAGAGAGAATTCAGGAGGTGGTGTTGAAGGGCTGGGATTACAAAGACTTGGATATGTTCTAGAAGA
HQ234499_Zika_virus_isolate_P6-740_polyprotein_gene,_partial_cds_9461	TTCTAGAAGAAATGAGCCGCACACCAGGAGGAAAGATGTATGCAGATGATACCGCTGGCTGGGACACCCGCATCAGTAGGTTTGATCTGGAGAATGAAGCTCTGATCACCAACCAAATGG
HQ234499_Zika_virus_isolate_P6-740_polyprotein_gene,_partial_cds_9571	AACCAAATGGAGAAAGGGCACAGGGCCTTGGCGTTGGCCATAATCAAGTACACATACCAAAACAAAGTGGTAAAGGTCCTTAGACCAGCTGAAAGAGGGAAGACAGTTATGGACATCATC
KF383117_Zika_virus_strain_ArD128000_polyprotein_gene,_complete_cds_3961	CTGATGGGTGCCACTTTCGCAGAAATGAACACTGGAGGAGATGTGGCTCACTTGGCATTGGTTGCGGCATTTAAAGTCAGACCAGCCTTGTTGGTTTCCTTTATCTTCAGAGCCAACTGG
KF383117_Zika_virus_strain_ArD128000_polyprotein_gene,_complete_cds_4181	CTTTGGCCTGGTTGGCAATACGAGCAATGGCCGTGCCACGCACTGACAATATCGCTCTAGCAACTCTGGCCGCTTTAACACCATTGGCCAGAGGCACACTGCTCGTGGCATGGAGAGCGG
KF383117_Zika_virus_strain_ArD128000_polyprotein_gene,_complete_cds_4291	TGGAGAGCGGGCCTCGCCACTTGTGGAGGGTTCATGCTTCTTTCCCTGAAAGGGAAAGGTAGTGTGAAGAAGAACCTGCCTTTTGTCATGGCCTTGGGGTTGGCCGCTGTGAGGATAGTG
KF383117_Zika_virus_strain_ArD128000_polyprotein_gene,_complete_cds_4401	GAGGATAGTGGACCCCATCAATGTGGTAGGACTACTGTTACTCACAAGGAGTGGAAAACGGAGCTGGCCCCCCAGTGAAGTGCTTACAGCTGTTGGCCTGATATGTGCACTGGCCGGAGG
KF383117_Zika_virus_strain_ArD128000_polyprotein_gene,_complete_cds_9571	AACCAGATGGAAGAAGGGCACAGAGCTCTGGCGTTGGCCGTGATTAAATACACATACCAAAACAAAGTGGTGAAGGTTCTCAGACCAGCTGAAGGAGGGAAAACAGTCATGGACATCATC
KF383117_Zika_virus_strain_ArD128000_polyprotein_gene,_complete_cds_9681	GGACATCATCTCAAGACAAGACCAGAGAGGGAGTGGACAAGTTGTTACTTATGCCCTCAACACATTCACCAACCTGGTGGTGCAGCTTATCCGGAACATGGAGGCTGAGGAAGTGCTAGA
KF383117_Zika_virus_strain_ArD128000_polyprotein_gene,_complete_cds_9791	AAGTGCTAGAGATGCATGATCTGTGGTTGTTGAGGAAACCAGAGAAAGTGACCAGATGGTTGCAGAGTAATGGATGGGACAGACTCAAACGGATGGCAGTTAGTGGAGATGACTGCGTTG
KF383117_Zika_virus_strain_ArD128000_polyprotein_gene,_complete_cds_9901	GACTGCGTTGTAAAGCCAATTGATGATAGGTTTGCACATGCCCTCAGGTTCTTGAATGACATGGGAAAGGTTAGGAAAGACACACAGGAATGGAAGCCCTCGACTGGATGGAGCAATTGG
KF383120_Zika_virus_strain_ArD142623_nonfunctional_polyprotein_gene,_partial_sequence_4511	TGGCTGGAGGGTTTGCCAAGGCAGACATAGAGATGGCTGGGCCCATGGCTGCAGTAGGCCTGCTAATTGTCAGCTATGTGGTCTCGGGAAAGAGTGTGGACATGTACATCGAAAGAGCAG
KF383120_Zika_virus_strain_ArD142623_nonfunctional_polyprotein_gene,_partial_sequence_4621	GAAAGAGCAGGTGACATCACATGGGAAAAGGATGCAGAAATAACTGGAAACAGTCCTAGGCTTGATGTGGCATTGGATGAGAGTGGTGACTTCTCTTTGCTGGAAGACGACGGACCCCCG
KF383120_Zika_virus_strain_ArD142623_nonfunctional_polyprotein_gene,_partial_sequence_4731	CGGACCCCCGATGAAGGAGATCATACTCAAAGTGGTTTTGATGGCCGTGTGCGGCGTGAGTCCCATAGCCATACCCTTTGCAGCTGGAGCTTGGTATGTGTACATCAAGACTGGCAAACG
KF383120_Zika_virus_strain_ArD142623_nonfunctional_polyprotein_gene,_partial_sequence_9791	AGGTGCTGGAGACACATGATCTGTGGACACTGAGGAAACCAGCAAAAGTCACCCGCTGGCTGGGTAACAATGGATGGCACAAGCTCAAACGGATGGCAGTTAGTGGAGATGACTGCGTTG
KF383120_Zika_virus_strain_ArD142623_nonfunctional_polyprotein_gene,_partial_sequence_10011	GAGCAATTGGGAGGAGGTCCCGTTCTGTTCTCACCACTTCAACAAGCTGCACCTCAAGGACGGGAGATCCATTGTGGTCCCCTGCCGCCACCAAGATGAACTGATTGGCCGAGCCCGTGT
KF383120_Zika_virus_strain_ArD142623_nonfunctional_polyprotein_gene,_partial_sequence_10121	GAGCCCGTGTCTCACCAGGGGCAGGATGGAGCATCCGTGAGACTGCTTGTCTGGAGAAGTCATACGCGCAAATGTGGCCTCTCCTCTACTTCCATATAAGAGATCTCAGGCTTATGGCGC
KF383120_Zika_virus_strain_ArD142623_nonfunctional_polyprotein_gene,_partial_sequence_10231	CTTATGGCGCACGCAATCTGCTCATCTGTGCCTGTCGATTGGGTCCCAACAGGGAAAACAACCTGCTCGATTCACGGGAAAGGAGAGTGGATGACC
KT365778_Zika_virus_strain_BeH819015_polyprotein_gene,_complete_cds_5061	AGATGTCAAGCAGGATCTGGTGTCATACTGTGGTCCATGGAAGCTAGATGCCGCCTGGGACGGGCACAGCGAGGTGCAGCTCTTGGCCGTGCCCCCCGGAGAGAGAGCGAGGAACATCCA
KT365778_Zika_virus_strain_BeH819015_polyprotein_gene,_complete_cds_5171	GGAACATCCAGACTCTGCCCGGAATATTTAAGACAAAGGATGGGGACATTGGAGCGGTTGCGCTGGATTACCCAGCAGGAACTTCAGGATCTCCAATCCTAGACAAGTGTGGGAGAGTGA
KT365778_Zika_virus_strain_BeH819015_polyprotein_gene,_complete_cds_5281	GGGAGAGTGATAGGACTTTATGGCAATGGGGTCGTGATCAAAAATGGGAGTTATGTTAGTGCCATCACCCAAGGGAGGAGGGAAGAAGAGACTCCTGTTGAGTGCTTCGAGCCCTCGATG
LC002520_Zika_virus_genomic_RNA,_complete_genome,_strain	TATGGAGAGAAGAGAGTGCTCAAACCGAGATGGATGGATGCTAGGGTCTGTTCAGACCATGCGGCCCTGAAGTCGTTCAAAGAATTCGCCGCTGGAAAAAGAGGAGCGGCTTTGGGAGTA
LC002520_Zika_virus_genomic_RNA,_complete_genome,_strain	TTTGGGAGTAATGGAGGCCCTGGGAACACTGCCAGGACACATGACAGAGAGGTTTCAGGAAGCCATTGACAACCTCGCCGTGCTCATGCGAGCAGAGACTGGAAGCAGGCCTTATAAGGC
LC002520_Zika_virus_genomic_RNA,_complete_genome,_strain	CTTATAAGGCAGCGGCAGCCCAACTGCCGGAGACCCTAGAGACCATTATGCTCTTAGGTTTGCTGGGAACAGTTTCACTGGGGATCTTCTTCGTCTTGATGCGGAATAAGGGCATCGGGA
LC002520_Zika_virus_genomic_RNA,_complete_genome,_strain	GGCATCGGGAAGATGGGCTTTGGAATGGTAACCCTTGGGGCCAGTGCATGGCTCATGTGGCTTTCGGAAATTGAACCAGCCAGAATTGCATGTGTCCTCATTGTTGTGTTTTTATTACTG
LC002520_Zika_virus_genomic_RNA,_complete_genome,_strain	TTTATTACTGGTGGTGCTCATACCCGAGCCAGAGAAGCAAAGATCTCCCCAAGATAACCAGATGGCAATTATCATCATGGTGGCAGTGGGCCTTCTAGGTTTGATAACTGCAAACGAACT
HQ234499_Zika_virus_isolate_P6-740_polyprotein_gene,_partial_cds_5501	TCCGCACGGTGATCCTGGCTCCAACCAGGGTTGTCGCTGCTGAAATGGAGGAAGCCCTTAGAGGGCTTCCAGTGCGTTACATGACAACAGCAGTTAATGTCACCCACTCTGGGACAGAAA
HQ234499_Zika_virus_isolate_P6-740_polyprotein_gene,_partial_cds_5611	GGGACAGAAATCGTTGATTTAATGTGCCATGCCACCTTCACTTCACGCCTACTACAACCCATTAGAGTCCCCAACTACAATCTTTACATTATGGATGAGGCCCACTTCACAGATCCCTCA
HQ234499_Zika_virus_isolate_P6-740_polyprotein_gene,_partial_cds_5721	AGATCCCTCAAGTATAGCAGCAAGAGGATACATATCAACAAGGGTTGAGATGGGCGAGGCGGCTGCCATCTTCATGACCGCCACACCACCAGGAACCCGCGACGCATTTCCGGACTCTAA
KF383117_Zika_virus_strain_ArD128000_polyprotein_gene,_complete_cds_441	GCTGCCAGCCGGACTTCTGCTGGGTCATGGACCCATTAGAATGGTTTTGGCGATACTAGCCTTTTTGAGATTTACAGCAATCAAGCCATCACTGGGCCTCATCAATAGATGGGGTTCAGT
KF383117_Zika_virus_strain_ArD128000_polyprotein_gene,_complete_cds_551	GGGGTTCAGTGGGGAAAAAAGAGGCTATGGAGATAATAAAGAAGTTTAAGAAAGATCTTGCTGCCATGTTGAGAATAATCAATGCTAGGAAGGAGAGGAAGAGACGTGGTGCAGACACCA
KF383117_Zika_virus_strain_ArD128000_polyprotein_gene,_complete_cds_5831	CTGACTCTAACTCACCAATCATGGACACAGAAGTGGAAGTCCCAGAGAGAGCCTGGAGCTCAGGCTTTGATTGGGTGACAGACCATTCTGGGAAAACAATTTGGTTTGTTCCAAGTGTGA
KF383117_Zika_virus_strain_ArD128000_polyprotein_gene,_complete_cds_5941	CCAAGTGTGAGAAACGGAAATGAAATCGCAGCCTGTCTGACAAAGGCTGGAAAGCGGGTTATACAGCTCAGCAGGAAGACTTTTGAGACAGAGTTTCAGAAGACAAAAAATCAAGAGTGG
KF383117_Zika_virus_strain_ArD128000_polyprotein_gene,_complete_cds_6051	TCAAGAGTGGGACTTTGTCATAACAACTGACATTTCAGAGATGGGTGCCAATTTCAAGGCTGACCGGGTCATCGATTCCAGGAGATGCCTAAAGCCAGTCATACTTGATGGTGAGAGAGT
KF383120_Zika_virus_strain_ArD142623_nonfunctional_polyprotein_gene,_partial_sequence_661	GCAGACACCAGCATCGGAATCATTGGCCTCCTGCTGACTACAGCCATGGCAGCAGAGATCACTAGACGCGGGAGTGCATACTACATGTACTTGGATAGGAGCGATGCCGGGAAGGCCATT
KF383120_Zika_virus_strain_ArD142623_nonfunctional_polyprotein_gene,_partial_sequence_771	GAAGGCCATTTCGTTTGCTACCACAATGGGAGTGAACAAGTGCCACGTACAGATCATGGACCTCGGGCACATGTGTGACGCCACCATGAGTTATGAGTGCCCTATGCTGGATGAGGGAGT
KF383120_Zika_virus_strain_ArD142623_nonfunctional_polyprotein_gene,_partial_sequence_881	ATGAGGGAGTGGAACCAGATGATGTCGATTGCTGGTGCAACACGACATCAACTTGGGTTGTGTACGGAACCTGTCATCACAAAAAAGGTGAGGCACGGCCCTCAAGAAGAGCTGTCACGC
KF383120_Zika_virus_strain_ArD142623_nonfunctional_polyprotein_gene,_partial_sequence_6161	GTGAGAGAGTCATCCTGGCTGGGCCCATGCCTGTCACGCATGCCAGTGCTGCTCAGAGGAGAGGACGTATAGGCAGGAACCCCAACAAACCTGGAGATGAGTACATGTATGGAGGTGGGT

KF383120_Zika_virus_strain_ArD142623_nonfunctional_polyprotein_gene,_partial_sequence_6271	GGAGGTGGGTGTGCAGAGACTGATGAAGACCATGCACACTGGCTTGAAGCAAGAATGCTTCTTGACAACATTTACCTCCAGGATGGCCTCATAGCCTCGCTCTATCGGCCTGAGGCCGAC
KF383120_Zika_virus_strain_ArD142623_nonfunctional_polyprotein_gene,_partial_sequence_6381	TGAGGCCGACAAGGTAGCCGCCATTGAGGGAGAGTTCAAGCTGAGGACAGAGCAAAGGAAGACCTTTGTGGAACTCATGAAGAGAGGAGACCTTCCCGTTTGGCTGGCCTATCAAGTAGC
KT365778_Zika_virus_strain_BeH819015_polyprotein_gene,_complete_cds_1101	CAGGAACCCTGGCTTCGCGTTAGCAGCAGCTGCCATCGCTTGGCTTTTGGGAAGCTCAACGAGCCAAAAAGTCATATACTTGGTCATGATACTGCTGATTGCCCCGGCATACAGCATCAG
KT365778_Zika_virus_strain_BeH819015_polyprotein_gene,_complete_cds_1211	ACAGCATCAGGTGCATAGGAGTCAGCAATAGGGACTTTGTGGAAGGTATGTCAGGTGGGACTTGGGTTGATGTTGTCTTGGAACATGGAGGTTGTGTCACCGTAATGGCACAGGACAAAC
KT365778_Zika_virus_strain_BeH819015_polyprotein_gene,_complete_cds_1321	CAGGACAAACCGACTGTCGACATAGAGCTGGTTACAACAACAGTCAGCAACATGGCGGAGGTAAGATCCTACTGCTATGAGGCATCAATATCAGACATGGCTTCGGACAGCCGCTGCCCA
KT365778_Zika_virus_strain_BeH819015_polyprotein_gene,_complete_cds_1431	CCGCTGCCCAACACAAGGTGAAGCCTACCTTGACAAGCAATCAGACACTCAATATGTCTGCAAAAGAACGTTAGTGGACAGAGGCTGGGGAAATGGATGTGGACTTTTTGGCAAAGGGAG
KT365778_Zika_virus_strain_BeH819015_polyprotein_gene,_complete_cds_1541	GCAAAGGGAGCCTGGTGACATGCGCTAAGTTTGCATGCTCCAAGAAAATGACCGGGAAGAGCATCCAGCCAGAGAATCTGGAGTACCGGATAATGCTGTCAGTTCATGGCTCCCAGCACA
KT365778_Zika_virus_strain_BeH819015_polyprotein_gene,_complete_cds_6601	CACGGAGAGAAAAGAGTGCTCAAACCGAGGTGGATGGACGCCAGAGTTTGTTCAGATCATGCGGCCCTGAAGTCATTCAAGGAGTTTGCCGCTGGGAAAAGAGGAGCGGCTTTTGGAGTG
KT365778_Zika_virus_strain_BeH819015_polyprotein_gene,_complete_cds_7041	CCTATTGCTGGTGGTGCTCATACCTGAGCCAGAAAAGCAAAGATCTCCCCAGGACAACCAAATGGCAATCATCATCATGGTAGCAGTAGGTCTTCTGGGCTTGATTACCGCCAATGAACT
DQ859059_Zika_virus_strain_MR_766_polyprotein_gene,_complete_cds_5061	GGTGTCATATTGTGGGCCTTGGAAGCTGGACGCAGCTTGGGACGGAGTTAGTGAGGTGCAGCTTCTGGCCGTACCCCCTGGAGAGAGAGCCAGAAACATTCAGACTCTGCCTGGAATATT
LC002520_Zika_virus_genomic_RNA,_complete_genome,_strain	CAGGACAAGCCAACAGTTGACATAGAGTTGGTCACGACGACGGTTAGTAACATGGCCGAGGTAAGATCCTATTGCTACGAGGCATCGATATCGGACATGGCTTCGGACAGTCGTTGCCCA
LC002520_Zika_virus_genomic_RNA,_complete_genome,_strain	GCAAAGGGAGCTTGGTGACATGTGCCAAGTTTACGTGTTCTAAGAAGATGACCGGGAAGAGCATTCAACCGGAAAATCTGGAGTATCGGATAATGCTATCAGTGCATGGCTCCCAGCATA
LC002520_Zika_virus_genomic_RNA,_complete_genome,_strain	CAGATTCTTGGAGTTTGAAGCCCTTGGATTCTTGAACGAGGACCATTGGATGGGAAGAGAAAACTCAGGAGGTGGAGTCGAAGGGTTAGGATTGCAAAGACTTGGATACATTCTAGAAGA
DQ859059_Zika_virus_strain_MR_766_polyprotein_gene,_complete_cds_7701	TTGGGGATGGGGGGAGGCTGGAGCTTTGATCACAGCAGCAACTTCCACCCTGTGGGAAGGCTCCCCAAACAAATACTGGAACTCCTCCACAGCCACCTCACTGTGCAACATCTTCAGAGG
DQ859059_Zika_virus_strain_MR_766_polyprotein_gene,_complete_cds_7811	TCTTCAGAGGAAGTTACTTGGCAGGAGCTTCCCTTATTTACACAGTGACAAGAAATGCCGGCCTGGTTAAGAGACGTGGAGGTGGAACGGGAGAAACTCTGGGAGAGAAGTGGAAAGCCC
DQ859059_Zika_virus_strain_MR_766_polyprotein_gene,_complete_cds_7921	TGGAAAGCCCGCCTGAATCAGATGTCGGCCTTGGAGTTCTACTCTTACAAAAAGTCAGGCATCACTGAAGTATGTAGAGAGGAGGCTCGCCGCGCCCTCAAGGATGGAGTGGCCACAGGA
DQ859059_Zika_virus_strain_MR_766_polyprotein_gene,_complete_cds_8141	GGGGCTGGAGTTATTATGCCGCCACCATCCGCAAAGTGCAGGAGGTGAGAGGATACACAAAGGGAGGTCCCGGTCATGAAGAGCCCATGCTGGTGCAAAGCTATGGGTGGAACATAATTC
HQ234499_Zika_virus_isolate_P6-740_polyprotein_gene,_partial_cds_2531	ACTCATTGGGCAAGGGCATCCATCAAATTTTTGGAGCAGCTTTCAAATCATTGTTTGGAGGAATGTCCTGGTTCTCACAAATCCTCATAGGAACGTTGCTGGTGTGGTTGGGTCTGAACA
HQ234499_Zika_virus_isolate_P6-740_polyprotein_gene,_partial_cds_2641	GGTCTGAACACAAAGAATGGATCTATTTCCCTTACGTGCTTGGCCTTAGGGGGAGTGTTGATCTTCCTATCTACAGCCGTCTCTGCTGATGTGGGGTGTTCGGTGGACTTCTCAAAGAAG
HQ234499_Zika_virus_isolate_P6-740_polyprotein_gene,_partial_cds_2751	CTCAAAGAAGGAAACGAGATGCGGTACGGGGGTGTTCGTCTATAACGACGTTGAAGCCTGGAGGGACAGGTACAAGTACCATCCTGACTCCCCTCGTAGATTGGCAGCAGCAGTCAAGCA
HQ234499_Zika_virus_isolate_P6-740_polyprotein_gene,_partial_cds_2861	CAGTCAAGCAGGCCTGGGAAGATGGGATCTGTGGGATCTCCTCTGTTTCAAGAATGGAAAACATTATGTGGAGATCAGTAGAAGGGGAGCTCAACGCAATTCTGGAAGAGAATGGAGTTC
HQ234499_Zika_virus_isolate_P6-740_polyprotein_gene,_partial_cds_2971	AATGGAGTTCAACTGACGGTCGTTGTGGGATCTGTAAAAAACCCCATGTGGAGAGGTCCGCAGAGGTTGCCTGTGCCTGTGAATGAGCTGCCCCACGGTTGGAAGGCCTGGGGGAAATCG
HQ234499_Zika_virus_isolate_P6-740_polyprotein_gene,_partial_cds_8031	CGCCCTCAAGGACGGAGTGGCAACAGGAGGCCATGCTGTGTCCCGAGGAAGCGCAAAGCTTAGATGGTTGGTGGAGAGAGGATACCTGCAGCCCTATGGAAAGGTCATTGATCTTGGATG
HQ234499_Zika_virus_isolate_P6-740_polyprotein_gene,_partial_cds_8141	ATCTTGGATGTGGCAGAGGGGGCTGGAGTTACTACGCCGCCACCATCCGCAAAGTTCAAGAGGTGAAAGGATACACAAAGGGAGGCCCTGGTCATGAAGAACCCACGTTGGTGCAAAGCT
HQ234499_Zika_virus_isolate_P6-740_polyprotein_gene,_partial_cds_8251	GTGCAAAGCTATGGATGGAACATAGTCCGTCTTAAGAGTGGGGTGGACGTCTTTCACATGGCGGCGGAGTCGTGTGACACTTTGCTGTGTGACATAGGTGAGTCATCATCTAGTCCTGAA
HQ234499_Zika_virus_isolate_P6-740_polyprotein_gene,_partial_cds_8361	TAGTCCTGAAGTGGAAGAAGCACGGACGCTCAGAGTACTCTCCATGGTGGGGGATTGGCTTGAAAAAAGACCAGGGGCCTTTTGTATAAAGGTGTTGTGCCCATACACCAGCACCATGAT
HQ234499_Zika_virus_isolate_P6-740_polyprotein_gene,_partial_cds_8471	GCACCATGATGGAAACCCTAGAGCGACTGCAGCGTAGGTATGGGGGAGGACTGGTCAGAGTGCCACTCTCCCGCAACTCTACACATGAGATGTACTGGGTCTCTGGAGCGAAAAGCAACA
DQ859059_Zika_virus_strain_MR_766_polyprotein_gene,_complete_cds_2641	GGGTCTATCTCCCTCACATGCTTAGCCCTGGGGGGAGTGATGATCTTCCTCTCCACGGCTGTTTCTGCTGACGTGGGGTGCTCGGTGGACTTCTCAAAAAAAGAAACGAGATGTGGCACG
KF383117_Zika_virus_strain_ArD128000_polyprotein_gene,_complete_cds_2861	CTGTTAAGCAGGCTTGGGAAGAGGGGATTTGTGGGATCTCCTCCGTTTCGAGAATGGAAAACATCATGTGGAAATCAGTGGAAGGGGAGCTTAATGCAATCCTAGAGGAGAATGGAGTCC
KF383117_Zika_virus_strain_ArD128000_polyprotein_gene,_complete_cds_3081	GGGGAAATCGTACTTTGTCAGAGCGGCAAAGACCAACAACAGTTTTGTTGTCGACGGAGACACACTGAAGGAGTGTCCGCTCAAACATAGAGCATGGAATAGCTTCCTTGTGGAGGATCA
KF383117_Zika_virus_strain_ArD128000_polyprotein_gene,_complete_cds_3191	TGGAGGATCACGGGTTTGGGATTTTCCACACCAGTGTTTGGCTGAAGGTCAGAGAGGACTACTCATTAGAGTGTGACCCAGCCGTCATAGGAACAGCTGTCAAGGGAAAGGAAGCTGCAC
KF383117_Zika_virus_strain_ArD128000_polyprotein_gene,_complete_cds_3301	GAAGCTGCACACAGTGATCTATGCTATTGGATTGAGAGTGAAAAGAATGACACATGGAGGCTGAGGAGGGCCCACCTGATTGAGATGAAAACATGTGAATGGCCAAAGTCTCACACATTG
KF383117_Zika_virus_strain_ArD128000_polyprotein_gene,_complete_cds_8581	AAAAGTAACATCATAAAGAGTGTGTCCACCACAAGTCAGCTCCTCTTGGGACGCATGGATGGGCCTAGGAGGCCAGTGAAATATGAGGAGGATGTGAACCTCGGCTCAGGCACACGAGCT
KF383117_Zika_virus_strain_ArD128000_polyprotein_gene,_complete_cds_8691	CACACGAGCTGTGGCAAGCTGCGCTGAGGCTCCCAACATGAAGATCATTGGTAGGCGCATTGAGAGAATCCGCAATGAACATGCAGAGACATGGTTCTTTGATGAAAACCACCCATACAG
KF383117_Zika_virus_strain_ArD128000_polyprotein_gene,_complete_cds_8801	ACCCATACAGGACATGGGCCTACCATGGGAGCTACGAAGCCCCCACGCAGGGGTCAGCGTCATCCCTCGTGAATGGGGTTGTTAGACTCTTGTCAAAGCCCTGGGATGTGGTGACTGGAG
KF383120_Zika_virus_strain_ArD142623_nonfunctional_polyprotein_gene,_partial_sequence_3191	TGGAGGACCATGGGTTTGGAGTTTTCCACACTAGTGTGTGGCTCAAAGTCAGGGAGGATTACTCATTAGAGTGTGATCCAGCCGTGATTGGAACAGCCATCAAAGGGAAGGAAGCTGCTC
KF383120_Zika_virus_strain_ArD142623_nonfunctional_polyprotein_gene,_partial_sequence_3301	GAAGCTGCTCACAGTGATCTTGGTTATTGGATTGAGAGCGAGAAGAACGACACCTGGAGACTGAAGAGGGCTCACATGATCGAGATGAAGACGTGTGAGTGGCCAAGATCTCACACCCTA
KF383120_Zika_virus_strain_ArD142623_nonfunctional_polyprotein_gene,_partial_sequence_3411	TCACACCCTATGGACAGATGGAGTAGAAGGAGAGTGATTGATCATACCCAAGTCCTTAGCTGGTCCACTCAGTCACCACAACACCAGAGAGGGTTACAGAACTCAAGTGAAAGGGCCATG
KF383120_Zika_virus_strain_ArD142623_nonfunctional_polyprotein_gene,_partial_sequence_3521	AAGGGCCATGGCATAGTGAAGAGCTCGAAATCCGGTTTGAGGAGTGCCCAGGAACCAAGGTTCACGTGGAGGAGACATGCGGAACTAGAGGACCATCTCTGAGATCAACTACTGCAAGTG
KF383120_Zika_virus_strain_ArD142623_nonfunctional_polyprotein_gene,_partial_sequence_8911	GTGACTGGAGTTACAGGAATAGCTATGACTGACACCACGCCATACGGCCAACAAAGAGTCTTCAAGGAAAAAGTTGACACTAGGGTGCCAGATCCCCAAGAAGGCACTCGCCAAGTAATG
KF383120_Zika_virus_strain_ArD142623_nonfunctional_polyprotein_gene,_partial_sequence_9021	CCAAGTAATGAACATGGTCTCGTCTTGGCTATGGAAGGAGCTGGGAAAACGCAAGCGGCCACGTGTCTGCACCAAAGAAGAGTTCATTAATAAGGTGCGCAGCAATGCAGCACTGGGAGC
KT365778_Zika_virus_strain_BeH819015_polyprotein_gene,_complete_cds_4181	CTTTGGCCTGGTTGGCAATACGAGCGATGGTTGTTCCACGCACTGATAACATCACCTTGGCAATCCTGGCTGCTCTGACACCACTGGCCCGGGGCACACTGCTTGTGGCGTGGAGAGCAG
KT365778_Zika_virus_strain_BeH819015_polyprotein_gene,_complete_cds_4291	TGGAGAGCAGGCCTTGCTACTTGCGGGGGGTTTATGCTCCTCTCTCTGAAGGGAAAAGGCAGTGTGAAGAAGAACTTACCATTTGTCATGGCCCTGGGACTAACCGCTGTGAGGCTGGTC
KT365778_Zika_virus_strain_BeH819015_polyprotein_gene,_complete_cds_9681	GGACATTATTTCGAGACAAGACCAAAGGGGGAGCGGACAAGTTGTCACTTACGCTCTTAACACATTTACCAACCTAGTGGTGCAACTCATTCGGAATATGGAGGCTGAGGAAGTTCTAGA
KT365778_Zika_virus_strain_BeH819015_polyprotein_gene,_complete_cds_9791	AAGTTCTAGAGATGCAAGACTTGTGGCTGCTGCGGAGGTCAGAGAAAGTGACCAACTGGTTGCAGAGCAACGGATGGGATAGGCTCAAACGAATGGCAGTCAGTGGAGATGATTGCGTTG
LC002520_Zika_virus_genomic_RNA,_complete_genome,_strain	GGAGAGTGTCACAGCTGTGTGTACAACATGATGGGAAAAAGAGAAAAGAAGCAAGGAGAGTTCGGGAAAGCAAAAGGTAGCCGCGCCATCTGGTACATGTGGTTGGGAGCCAGATTCTTG
DQ859059_Zika_virus_strain_MR_766_polyprotein_gene,_complete_cds_7151	TGGAGAGAACAAAAAATGACATAGCTCATCTGATGGGAAAGAGAGAAGAGGGAACAACCGTGGGATTCTCAATGGACATCGATCTGCGACCAGCCTCCGCATGGGCTATTTATGCCGCAT
DQ859059_Zika_virus_strain_MR_766_polyprotein_gene,_complete_cds_7591	GTGGTAACTGACATTGACACAATGACAATTGACCCCCAAGTGGAGAAGAAGATGGGACAAGTGCTACTTATAGCAGTGGCTGTCTCCAGTGCTGTGTTGCTGCGGACCGCTTGGGGATGG
HQ234499_Zika_virus_isolate_P6-740_polyprotein_gene,_partial_cds_1981	GTGGTTCTAGGGAGTCAAGAAGGAGCCGTTCACACGGCTCTTGCTGGAGCCCTGGAGGCTGAGATGGATGGTGCAAAGGGAAGGCTGTCCTCTGGCCACTTGAAATGTCGCTTGAAAATG
HQ234499_Zika_virus_isolate_P6-740_polyprotein_gene,_partial_cds_2091	CTTGAAAATGGACAAACTTAGATTGAAGGGCGTGTCATACTCCTTATGTACCGCGGCGTTCACATTCACCAAGATCCCGGCTGAAACGCTGCATGGGACAGTCACAGTGGAGGTACAGTA
HQ234499_Zika_virus_isolate_P6-740_polyprotein_gene,_partial_cds_2201	AGGTACAGTATGCAGGGACAGATGGACCCTGCAAGGTTCCAGCTCAGATGGCGGTGGATATGCAAACTCTGACCCCAGTTGGGAGGTTGATAACCGCTAACCCTGTGATCACTGAAAGCA
HQ234499_Zika_virus_isolate_P6-740_polyprotein_gene,_partial_cds_2311	ACTGAAAGCACTGAGAATTCAAAGATGATGTTGGAACTTGACCCACCATTTGGGGATTCTTACATTGTCATAGGAGTTGGGGATAAGAAGATCACCCACCACTGGNACAGGAGTGGCAGC
HQ234499_Zika_virus_isolate_P6-740_polyprotein_gene,_partial_cds_2421	GAGTGGCAGCACCATCGGAAAAGCATTTGAAGCCACTGTGAGAGGCGCCAAGAGAATGGCAGTCTTGGGAGACACAGCCTGGGACTTTGGATCAGTCGGAGGTGCTCTCAACTCATTGGG
DQ859059_Zika_virus_strain_MR_766_polyprotein_gene,_complete_cds_1651	TCCCAGCACAGTGGGATGATTGTTAATGATGAAAACAGAGCGAAGGTCGAGGTTACGCCCAATTCACCAAGAGCAGAAGCAACCCTGGGAGGCTTTGGAAGCTTAGGACTTGATTGTGAA
DQ859059_Zika_virus_strain_MR_766_polyprotein_gene,_complete_cds_1871	GGCATGCTGGGGCAGACACTGGAACTCCACATTGGAACAACAAGGAGGCATTAGTGGAATTCAAGGACGCCCACGCCAAGAGGCAAACCGTCGTGGTTTTGGGGAGCCAGGAAGGAGCCG
HQ234499_Zika_virus_isolate_P6-740_polyprotein_gene,_partial_cds_7481	TAGTGGCCATCATTCTGCTCGTGGCGCACTACATGTACTTGATCCCAGGTCTACAGGCAGCAGCGGCGCGCGCTGCCCAGAAGAGAACGGCAGCTGGCATCATGAAGAACCCTGTTGTGG
HQ234499_Zika_virus_isolate_P6-740_polyprotein_gene,_partial_cds_7591	CCTGTTGTGGATGGAATAGTGGTGACTGACATTGACACAATGACAATTGACCCCCAAGTGGAGAAAAAGATGGGACAAGTGCTACTCATAGCAGTAGCCATCTCCAGTGCCGTTCTGCTG
HQ234499_Zika_virus_isolate_P6-740_polyprotein_gene,_partial_cds_7701	CGTTCTGCTGCGCACCGCCTGGGGGTGGGGGGAGGCTGGGGCCCTGATCACAGCCGCAACTTCCACTTTGTGGGAAGGCTCTCCGAATAAATACTGGAACTCCTCCACAGCCACTTCACT
HQ234499_Zika_virus_isolate_P6-740_polyprotein_gene,_partial_cds_7811	CCACTTCACTGTGTAACATTTTAGGGGAAGTTACTTGGCTGGAGCTTCTCTTATTTACACAGTAACAAGAAACGCTGGCCTGGTCAAGAGACGTGGAGGTGGAACGGGAGAGACCCTGG
HQ234499_Zika_virus_isolate_P6-740_polyprotein_gene,_partial_cds_7921	GAGACCCTGGGGGAGAAATGGAAGGCCCGCCTGAACCAGATGTCGGCCCTGGAGTTTTACTCCTACAAAAAGTCAGGCATCACCGAAGTGTGCAGAGAAGAAGCCCGCCGCGCCCTCAAG
KF383117_Zika_virus_strain_ArD128000_polyprotein_gene,_complete_cds_2641	GGTCTGAACACAAAGAATGGATCTATCTCCCTCACATGCTTAGCCCTGGGGGGAGTGATGATTTTCCTTTCCACGGCTGTTTCTGCTGATGTTGGGTGCTCGGTGGACTTCTCAAAAAG
KF383120_Zika_virus_strain_ArD142623_nonfunctional_polyprotein_gene,_partial_sequence_2641	GGCTTGAATACAAAAAATGGATCCATTTCCCTTGCATGTCTGGCGCTTGGAGGGGCGATGATCTTTCTCTCCACGGCCGTCTCGGCAGACGTGGGGTGCTCAGTTGACTTCGCTAGAAAA
KF383120_Zika_virus_strain_ArD142623_nonfunctional_polyprotein_gene,_partial_sequence_2751	CGCTAGAAAAGAGACAAGATGTGGCACAGGAGTTTTCGTCTACAACGACGTTGATGCCTGGAGGGACAGATACAAGTACCATCCAGACTCTCCACGTAGACTGGCGGCGGCTGTTAAGCA
KF383120_Zika_virus_strain_ArD142623_nonfunctional_polyprotein_gene,_partial_sequence_2861	CTGTTAAGCAAGCTTGGGAAGATGGAATCTGCGGGATTTCCTCCGTCTCAAGGATGGAAAACATCATGTGGAAGTCGGTGGAGGGAGAGCTCAATGCTATACTCGAAGAGAACGGAGTGC
KF383120_Zika_virus_strain_ArD142623_nonfunctional_polyprotein_gene,_partial_sequence_2971	AACGGAGTGCAGCTCACAGTTGTTGTAGGACCTGTAAAAAATCCTATGTGGAGAGCACCACAACGGCTACCGGTGCCTGTGAATGAGCTTCCTCATGGATGGAAAGCCTGGGGCAAATCA
KF383120_Zika_virus_strain_ArD142623_nonfunctional_polyprotein_gene,_partial_sequence_3081	GGGCAAATCATACTTCATCAAAGCAGCAAAGACCAACAATAGCTTTGTGGTCGATGGCGACACCTTGAAAGAGTGTCCACTCAAACAGAGGGCCTGGAACAGCTTCCTTGTGGAGGACCA
KF383120_Zika_virus_strain_ArD142623_nonfunctional_polyprotein_gene,_partial_sequence_8141	ACCTAGGATGTGGTAGGGGAGGCTGGAGCTACTATGCGGCAACCATCCGCAAAGTCCAAGAGGTCAGAGGATACACCAAAGGAGGACCTGGCCATGAAGAGCCAATGCTAGTGCAGAGCT
KF383120_Zika_virus_strain_ArD142623_nonfunctional_polyprotein_gene,_partial_sequence_8251	GTGCAGAGCTATGGTTGGAACATAGTCCGCCTCAAGAGCGGAGTGGACGTGTTCCACATGGCAGCTGAGCCTTGCGACACCCTCCTATGTGACATTGGAGAGTCATCATCTAGTCCAGAG
KF383120_Zika_virus_strain_ArD142623_nonfunctional_polyprotein_gene,_partial_sequence_8361	TAGTCCAGAGGTGGAGGAAACTAGAACACTCAGAGTGCTTTCCATGGTGGGGGATTGGTTGGAGAAGAGACCAGGAGCCTTTTGCATAAAAGTCCTGTGCCCATACACGAGCACCATGAT
KT365778_Zika_virus_strain_BeH819015_polyprotein_gene,_complete_cds_8801	ACCCATATAGGACATGGGCTTACCATGGAAGCTATGAGGCCCCCACACAAGGGTCAGCGTCCTCTCTAATAAACGGGGTTGTCAGGCTCCTGTCAAAACCCTGGGATGTGGTGACTGGAG
KT365778_Zika_virus_strain_BeH819015_polyprotein_gene,_complete_cds_9021	TCAGGTTATGAGCATGGTCTCTTCCTGGTTGTGGAAAGAGCTAGGCAAACACAAACGGCCACGAGTCTGTACCAAAGAAGAGTTCATCAACAAGGTTCGTAGCAATGCAGCATTAGGGGC
DQ859059_Zika_virus_strain_MR_766_polyprotein_gene,_complete_cds_991	GCCGTCACGCTCCCATCTCACTCCACAAGGAAATTGCAAACGCGGTCGCAGACTTGGCTAGAATCAAGAGAATACACAAAGCACCTGATCAAGGTTGAAAATTGGATATTCAGGAACCCT
LC002520_Zika_virus_genomic_RNA,_complete_genome,_strain	CCACCTCACTGTGCAACATCTTCAGAGGAAGCTATCTGGCAGGAGCTTCCCTTATCTATACAGTGACGAGAAACGCTGGCCTGGTTAAGAGACGTGGAGGTGGGACGGGAGAGACTCTGG
LC002520_Zika_virus_genomic_RNA,_complete_genome,_strain	GAGACTCTGGGAGAGAAGTGGAAAGCTCGTCTGAATCAGATGTCGGCCCTGGAGTTCTACTCTTATAAAAAGTCAGGTATCACTGAAGTGTGTAGAGAGGAGGCTCGCCGTGCCCTCAAG
HQ234499_Zika_virus_isolate_P6-740_polyprotein_gene,_partial_cds_6491	ATCAGGTTGCATCTGCCGGAATAACCTACACAGATAGAAGATGGTGTTTTGATGGCACGACCAACAACACCATAATGGAAGACAGTGTGCCGGCAGAGGTGTGGACCAGATACGGAGAGA
KF383117_Zika_virus_strain_ArD128000_polyprotein_gene,_complete_cds_7151	CAAATGAACTCGGATGGCTGGAAAGGACAAAAAGTGATATAGCTTATCTGATGGGAAGGAAAGAAGAGGGGACAACCATAGGATTCTCAATGGATATTGATCTGCGGCCAGCCTCTGCCT
KF383120_Zika_virus_strain_ArD142623_nonfunctional_polyprotein_gene,_partial_sequence_1651	TCCCAACACAGCGGGATGATTGTGAATGACATAGGACATGAGACCGATGAAAACAGAGCAAAAGTGGAGGTCACACCAAACTCACCAAGAGCTGAAGCGACCCTAGGGGGCTTCGGGAGT
KF383120_Zika_virus_strain_ArD142623_nonfunctional_polyprotein_gene,_partial_sequence_1761	CTTCGGGAGTTTGGGACTCGATTGTGAGCCAAGGACCGGTTTAGATTTCTCCGACCTGTACTATTTAACCATGAACAACAAGCATTGGTTGGTGCACAAGGAGTGGTTCCACGACATTCC
KF383120_Zika_virus_strain_ArD142623_nonfunctional_polyprotein_gene,_partial_sequence_1871	ACGACATTCCATTGCCATGGCACGCTGGAGCGGACACAGGGACTCCCCATTGGAACAACAAAGAGGCATTGGTTGAGTTCAAGGATGCCCACGCCAAAAGACAGACTGTTGTCGTGTTGG
KF383120_Zika_virus_strain_ArD142623_nonfunctional_polyprotein_gene,_partial_sequence_1981	GTCGTGTTGGGAAGTCAGGAGGGTGCCGTCCACACAGCCCTGGCAGGAGCTTTGGAAGCGGAGATGGATGGAGCCAAAGGAAGGCTGTTCTCAGGTCATTTGAAGTGCCGGATAAAGATG
KF383120_Zika_virus_strain_ArD142623_nonfunctional_polyprotein_gene,_partial_sequence_7041	TCTGTTGTTGGTCGTGCTCATACCCGAGCCGGAGAAACAGCGATCCCCGCAAGACAACCAAATGGCCATCATCATCATGGTCGCGGTGGGACTGCTGGGCCTCATTGCGGCCAACGAACT
KF383120_Zika_virus_strain_ArD142623_nonfunctional_polyprotein_gene,_partial_sequence_7151	CCAACGAACTTGGCTGGCTGGAAAGAACAAAGAGTGACATAGCTCACCTAATGGGCAGGAAGGATGAGGAGAGGATGGTGGGGTTCTCAATTGACATAGATCTGCGGCCAGCATCGGCAT
KF383120_Zika_virus_strain_ArD142623_nonfunctional_polyprotein_gene,_partial_sequence_7261	GCATCGGCATGGGCCATTTATGCCGCATTGACAACCCTCATCACCCCGGCCGTTCAACATGCAGTCACCACCTCCTACAACAACTACTCCCTTATGGCGATGGCAACCCAAGCGGGTGTG
KF383120_Zika_virus_strain_ArD142623_nonfunctional_polyprotein_gene,_partial_sequence_7371	AGCGGGTGTGTTGTTCGGCATGGGAAAGGGGATGCCTTTCTACACCTGGGATTTTGGTGTTCCACTGCTGATGATTGGGTGTTATTCTCAATTGACACCTCTCACTCTCATAGTAGCCAT
KF383120_Zika_virus_strain_ArD142623_nonfunctional_polyprotein_gene,_partial_sequence_7481	TAGTAGCCATCACCCTGCTAGTGGCTCATTACATGTACTTGATTCCAGGCCTGCAGGCAGCAGCAGCACGTGCTGCCCAGAAGAGGACAGCAGCTGGCATCATGAAGAATCCCGTTGTGG
DQ859059_Zika_virus_strain_MR_766_polyprotein_gene,_complete_cds_7041	AATACCAGAGCCAGAGAAGCAAAGATCCCCCCAGGACAATCAGATGGCAATCATTATTATGGTGGCAGTGGGCCTTTTGGGGTTGATAACTGCAAATGAACTTGGATGGCTGGAGAGAAC
HQ234499_Zika_virus_isolate_P6-740_polyprotein_gene,_partial_cds_1651	TCCCAGCACAGTGGGATGATTGTTAATGACANAGGACATGAAACTGATGAGAATAGAGCGAAGGTTGAGATAACGCCCAATTCACCAAGAGCCGAAGCCACCCTGGGAGGTTTTGGAAGC
HQ234499_Zika_virus_isolate_P6-740_polyprotein_gene,_partial_cds_1761	TTTTGGAAGCCTAGGACTTGATTGTGAACCGAGGACAGGCCTTGACTTTTCAGATTTGTATTACTTGACTATGAATAACAAGCATTGGTTGGTGCACAAGGAGTGGTTCCATGACATTCC
HQ234499_Zika_virus_isolate_P6-740_polyprotein_gene,_partial_cds_1871	ATGACATTCCACTACCTTGGCATGCTGGGGCAGACACCGGAACTCCACATTGGAACAACAAAGAAGCATTGGTAGAGTTCAAGGACGCACATGCCAAAAGGCAAACTGTCGTGGTTCTAG
HQ234499_Zika_virus_isolate_P6-740_polyprotein_gene,_partial_cds_7151	CCAATGAACTCGGATGGTTGGAGAGAACAAAAAGTGACCTAGGCCATCTAATGGGAAGGAGAGAGGAGGGGGCAACCATGGGATTCTCAATGGACATTGACTTGCGGCCAGCCTCAGCTT
HQ234499_Zika_virus_isolate_P6-740_polyprotein_gene,_partial_cds_7261	GCCTCAGCTTGGGCTATCTATGCCGCTCTGACAACTCTCATCACCCCAGCCGTCCAACATGCGGTAACCACTTCATACAACAACTACTCCTTAATGGCGATGGCCACGCAAGCCGGAGTG
HQ234499_Zika_virus_isolate_P6-740_polyprotein_gene,_partial_cds_7371	AGCCGGAGTGTTGTTTGGCATGGGCAAAGGGATGCCATTCTATGCGTGGGACTTCGGAGTCCCGCTGCTAATGATGGGTTGCTACTCACAATTAACACCCTTGACCTTAATAGTGGCCAT
KF383117_Zika_virus_strain_ArD128000_polyprotein_gene,_complete_cds_2091	CCTAAAAATGGACAAGCTTAGGTTGAAGGGTGTGTCATATTCCCTGTGTACTGCAGCGTTCACATTTACCAAGGTCCCAGCTGAAACATTGCATGGAACAGTTACAGTGGAGGTGCAGTA
KF383120_Zika_virus_strain_ArD142623_nonfunctional_polyprotein_gene,_partial_sequence_2091	GATAAAGATGGACAAGCTTAGGTTGAAGGGTGTCTCATACTCATTGTGCACTGCCGCCTTCACATTCTCAAAGGTTCCGGCTGAAACGCTGCATGGGACAGTCACAGTTGAGGTGCAGTA
KF383120_Zika_virus_strain_ArD142623_nonfunctional_polyprotein_gene,_partial_sequence_2201	AGGTGCAGTATGCGGGCAGAGATGGACCTTGCAAGGTGCCAGCCCAAATGGCAGTGGATATGCAGACTCTTACCCCAGTGGGAAGGTTGATAACGGCAAATCCCATCATAACTGAGAGCA
KF383120_Zika_virus_strain_ArD142623_nonfunctional_polyprotein_gene,_partial_sequence_2311	ACTGAGAGCACTGAAAACTCGAGAATGATGTTGGAGCTGGACCCACCCTTTGGAGACTCTTACATTGTCATTGGGGTTGGAGATAAGAAAATTACCCATCATTGGCACAGAAGTGGAAGC
KF383120_Zika_virus_strain_ArD142623_nonfunctional_polyprotein_gene,_partial_sequence_2421	AAGTGGAAGCACGATTGGGAAGGCATTTGAGGCCACTGTGAGAGGTGCAAAAAGGATGGCAGTCCTGGGAGATACCGCCTGGGATTTTGGTTCTGTTGGAGGCGTGTTCAATTCTCTGGG
KF383120_Zika_virus_strain_ArD142623_nonfunctional_polyprotein_gene,_partial_sequence_2531	ATTCTCTGGGCAAGGGAGTTCATCAGGTCTTCGGAGCAGCCTTTAAATCCTTATTTGGCGGAATGTCCTGGTTTTCACAAATTCTTATTGGCACACTTTTAGTGTGGTTGGGCTTGAATA
KF383120_Zika_virus_strain_ArD142623_nonfunctional_polyprotein_gene,_partial_sequence_7811	CCACATCTCTGTGTAACATCTTTAGGGGCAGTTACCTGGCTGGCGCTTCCCTTATTTACACTGTGACAAGGAACGCTGGTCTAGTCAAGAGGCGCGGAGGAGGAACAGGAGAGACCCTCG
KF383120_Zika_virus_strain_ArD142623_nonfunctional_polyprotein_gene,_partial_sequence_7921	GAGACCCTCGGAGAAAAGTGGAAATCTCGATTGAATCAGATGTCAGCCCTGGAGTTCTACTCATACAAAAAGTCAGGAATAACTGAAGTGTGCAGGGATGAGGCTCGGAGGGCCTTGAAG
KF383120_Zika_virus_strain_ArD142623_nonfunctional_polyprotein_gene,_partial_sequence_8031	GGCCTTGAAGGACGGAGTGGCTACTGGAGGACACGCAGTATCCCGAGGAAGCGCAAAGCTCAGATGGCTTGTGGAGAGAGGGTACCTGCAGCCACATGGGAAGGTTGTTGACCTAGGATG
KT365778_Zika_virus_strain_BeH819015_polyprotein_gene,_complete_cds_3191	TGGAGGATCATGGGTTCGGGGTATTTCACACTAGTGTCTGGCTCAAGGTTAGAGAAGATTATTCATTAGAGTGTGATCCAGCCGTTATTGGAACAGCTGTTAAGGGAAAGGAGGCTGTAC
KT365778_Zika_virus_strain_BeH819015_polyprotein_gene,_complete_cds_8691	CACGCGGGCTGTGGTAAGCTGCGCTGAAGCTCCCAACATGAAGATCATTGGTAACCGCATTGAAAGGATCCGCAGTGAGCACGCGGAAACGTGGTTCTTTGACGAGAACCACCCATATAG
HQ234499_Zika_virus_isolate_P6-740_polyprotein_gene,_partial_cds_4731	TGGTCCCCCCATGAGAGAGATCATACTCAAGGTGGTCCTGATGACCATCTGTGGCATGAACCCAATAGCCATACCCTTTGCAGCTGGAGCGTGGTATGTGTATGTGAAGACTGGAAAGAG
HQ234499_Zika_virus_isolate_P6-740_polyprotein_gene,_partial_cds_4841	CTGGAAAGAGGAGTGGTGCTCTATGGGATGTGCCTGCTCCCAAGGAAGTAAAAAAGGGGGAGACCACAGATGGAGTGTATAGAGTGATGACTCGCAGACTGCTAGGTTCAACACAAGTTG
HQ234499_Zika_virus_isolate_P6-740_polyprotein_gene,_partial_cds_10231	CTGATGGCCAACGCCATTTGTTCATCTGTGCCAGTTGACTGGGTTCCAACTGGGAGAACCACCTGGTCAATCCATGGAAAGGGAGAATGGATGACCACTGAGGACATGCTTGTGGTGTGG
HQ234499_Zika_virus_isolate_P6-740_polyprotein_gene,_partial_cds_10341	TGTGGTGTGGAACAGAGTGTGGATTGAGGAGAACGACCACATGGAGGACAAGACCCCAGTCACGAAATGGACAGACATTCCCTATTTGGGAAAAAGGGAAGACTTATGGTGTGGATCTCT
KF383120_Zika_virus_strain_ArD142623_nonfunctional_polyprotein_gene,_partial_sequence_5391	GCCATCAATGTTGAGGAAGAAGCAGTTAACAGTCCTGGATTTGCACCCTGGAGCTGGAAAGACCCGGAGGGTCCTTCCAGAGATAGTACGCGAGGCAATAAAGAGGAGGCTCCGCACTGT
KF383120_Zika_virus_strain_ArD142623_nonfunctional_polyprotein_gene,_partial_sequence_5501	TCCGCACTGTAATCTTAGCACCAACAAGGGTAGTGGCGGCTGAGATGGAAGAGGCCTTAAGGGGCCTTCCAGTTCGGTATATGACGCCAGCCATAAATGTGGCCCACTCTGGGACCGAGA
KF383120_Zika_virus_strain_ArD142623_nonfunctional_polyprotein_gene,_partial_sequence_5611	GGGACCGAGATTGTTGACCTGATGTGTCATGCCACATTCACCTCGCGTCTGTTGCAGCCTATCAGAGTCCCAAACTACAACTTGTACATCATGGATGAGGCCCATTTCACGGATCCATCG
KF383120_Zika_virus_strain_ArD142623_nonfunctional_polyprotein_gene,_partial_sequence_5721	GGATCCATCGAGCATTGCGGCAAGAGGCTACATCTCGACCAGAGTGGAAATGGGGGAAGCAGCAGCCATCTTCATGACAGCCACGCCGCCAGGCACCAGGGATGCTTTTCCAGATTCCAA
KF383120_Zika_virus_strain_ArD142623_nonfunctional_polyprotein_gene,_partial_sequence_5831	CAGATTCCAACTCCCCTATCATGGACACAGAAATTGAGGTCCCAGAAAGAGCCTGGAACTCGGGTTTCGAATGGGTGACAGATCACGCTGGCAGAACAGTGTGGTTTGTTCCGAGTGTGA
KT365778_Zika_virus_strain_BeH819015_polyprotein_gene,_complete_cds_661	GCAGATACTAGTGTCGGAATTGTTGGCCTCCTGCTGACCACAGCTATGGCAGCGGAGGTCACTAGACGTGGGAGTGCATACTATATGTACTTGGACAGAAACGATGCTGGGGAGGCCATA
KT365778_Zika_virus_strain_BeH819015_polyprotein_gene,_complete_cds_771	GGAGGCCATATCTTTTCCAACCACATTGGGGATGAATAAGTGTTATATACAGATCATGGATCTTGGACACATGTGTGATGCCACCATGAGCTATGAATGCCCTATGCTGGATGAGGGGGT
LC002520_Zika_virus_genomic_RNA,_complete_genome,_strain	GAAAGAGCAGGTGACATCACATGGGAAAAGGACGCGGAAGTCACTGGAAACAGTCCTCGGCTTGACGTGGCACTGGATGAGAGTGGTGATTTCTCCTTGGTAGAGGAAGATGGTCCACCC
LC002520_Zika_virus_genomic_RNA,_complete_genome,_strain	GAGCTCGCGTCTCACCAGGGGCAGGATGGAGCATCCGGGAGACTGCCTGTCTTGCAAAATCATATGCGCAGATGTGGCAGCTCCTTTATTTCCACAGAAGAGACCTTCGACTGATGGCTA
DQ859059_Zika_virus_strain_MR_766_polyprotein_gene,_complete_cds_8251	AACATAATTCGTCTCAAGAGTGGAGTGGACGTCTTCCACATGGCGGCTGAGTCGTGTGACACTTTGCTGTGTGACATAGGTGAGTCATCATCCAGTCCTGAAGTGGAGGAGACGCGAACA
DQ859059_Zika_virus_strain_MR_766_polyprotein_gene,_complete_cds_8361	GACGCGAACACTCAGAGTGCTCTCCATGGTGGGGGACTGGCTTGAGAAGAGACCAGGGGCCTTCTGCATAAAGGTGTTATGCCCATACACCAGCACCATGATGGAGACCATGGAGCGACT
DQ859059_Zika_virus_strain_MR_766_polyprotein_gene,_complete_cds_8691	CTGTGCTGAGGCTCCCAACATGAAGGTCATTGGTAGGCGCATTGAGAGAATCCGTAGTGAACATGCAGAAACATGGTTCTTTGATGAAAACCATCCATACAGGACATGGGCCTACCACGG
DQ859059_Zika_virus_strain_MR_766_polyprotein_gene,_complete_cds_2861	AAGAGGGGATTTGTGGGATCTCCTCTGTTTCTAGAATGGAAAACATAATGTGGAAATCAGTGGAAGGAGAGCTCAATGCAATCCTAGAGGAGAATGGAGTCCAACTGACAGTTGTTGTGG
DQ859059_Zika_virus_strain_MR_766_polyprotein_gene,_complete_cds_3081	TAGGGCGGCAAAGACCAACAACAGTTTTGTTGTCGACGGTGACACATTGAAGGAATGTCCGCTCAAGCACAGAGCATGGAACAGCTTCCTCGTGGAGGATCACGGGTTTGGGGTCTTCCA
DQ859059_Zika_virus_strain_MR_766_polyprotein_gene,_complete_cds_3191	GGGTCTTCCACACCAGTGTTTGGCTTAAGGTTAGAGAAGATTACTCACTGGAGTGTGACCCAGCCGTCATAGGAACAGCTGTTAAGGGAAAGGAGGCCGCGCACAGTGATCTAGGCTATT
KF383120_Zika_virus_strain_ArD142623_nonfunctional_polyprotein_gene,_partial_sequence_9351	CAGGTTTTTGGAGTTTGAATCACTGGGGTTTCTGAACGAAGATCACTGGATGGGAAGAGAAAACTCTGGAGGCGGAGTTGAAGGACTGGGACTGCAAAGACTTGGCTATATCCTTGAGGA
KF383120_Zika_virus_strain_ArD142623_nonfunctional_polyprotein_gene,_partial_sequence_9461	TCCTTGAGGAGATGAGCCGGGCACCAGGAGGAAAGATGTACGCAGATGACACTGCGGGTTGGGACACCAGAATTAGCAAGTTTGACTTGGAAAATGAGGCCCTGATCACCAACCAAATGG
LC002520_Zika_virus_genomic_RNA,_complete_genome,_strain	AGATCGCCGAACAGCGGCGGCCGGTGTGGGGAAATCCATGGTTTCT
DQ859059_Zika_virus_strain_MR_766_polyprotein_gene,_complete_cds_9351	AGCCCTTGGATTCTTGAACGAGGACCATTGGATGGGAAGAGAAAACTCAGGAGGTGGTGTCGAAGGGTTGGGACTGCAAAGACTTGGATACGTTCTAGAAGAAATGAGCCGGGCACCAGG
DQ859059_Zika_virus_strain_MR_766_polyprotein_gene,_complete_cds_9461	GGGCACCAGGAGGAAAGATGTATGCAGATGACACCGCTGGCTGGGACACCCGCATTAGCAAGTTTGATTTGGAGAATGAAGCCTTGATTACTAACCAAATGGATGAAGGGCACAGAACTC
DQ859059_Zika_virus_strain_MR_766_polyprotein_gene,_complete_cds_9571	CACAGAACTCTGGCGTTGGCCGTGATTAAGTACACATACCAAAACAAAGTGGTGAAGGTCCTCAGACCAGCTGAAGGAGGAAAAACAGTCATGGACATCATTTCAAGACAAGACCAGAGG
DQ859059_Zika_virus_strain_MR_766_polyprotein_gene,_complete_cds_9791	ACTTATGGCTGTTGAGGAAGCCAGAGAAAGTAACCAGATGGCTGCAGAGTAGCGGATGGGACAGACTCAAACGAATGGCAGTCAGTGGTGATGACTGTGTTGTAAAGCCAATTGATGACA
KF383117_Zika_virus_strain_ArD128000_polyprotein_gene,_complete_cds_10341	CATGGTGTGGAATAGAGTGTGGATTGAGGAGAACGACCACATGGAGGACAAGACCCCTGTTCCAAAATGGACAGAAATTCCCTACTTGGGGAAAAGGGAGTCCCTATGGTGTGGATACAT
KF383120_Zika_virus_strain_ArD142623_nonfunctional_polyprotein_gene,_partial_sequence_4841	CTGGCAAACGGAGTGGAGCCCTCTGGGACATACCGGCACCCAAGGAAGTGAAGAAAGGGGAAACAACTGATGGCGTGTATCGTGTTATGACCCGCAGACTGTTGGGCTCAACACAGGTTG
KF383120_Zika_virus_strain_ArD142623_nonfunctional_polyprotein_gene,_partial_sequence_4951	ACACAGGTTGGAGTGGGAGTCATGCAGGAGGGGGTTTTCCACACAATGTGGCACGTCACAAAAGGGGCTGCGCTGCGGAGTGGAGAAGGGAGACTGGATCCGTACTGGGGTGATGTGAAG
KF383120_Zika_virus_strain_ArD142623_nonfunctional_polyprotein_gene,_partial_sequence_5061	TGATGTGAAGCAAGACCTGGTGTCATACTGCGGACCTTGGAAGTTGGATGCCGCTTGGGACGGACTCAGCGAGGTCCAACTGTTGGCTGTTCCTCCAGGAGAAAGAGCTAGAAATGTCCA
KF383120_Zika_virus_strain_ArD142623_nonfunctional_polyprotein_gene,_partial_sequence_5171	GAAATGTCCAAACTCTCCCTGGAATCTTCAAGACCAAAGATGGGGACATTGGAGCAGTGGCCTTAGACTACCCAGCGGGGACATCTGGATCCCCAATCCTGGATAAAAACGGAAGAGTCA
KF383120_Zika_virus_strain_ArD142623_nonfunctional_polyprotein_gene,_partial_sequence_5281	GGAAGAGTCATAGGACTCTATGGCAATGGAGTGGTGATCAGAAATGGGAGCTATGTGAGCGCCATAACACAAGGAAAGAGAGAGGAAGAGGCTCCGTTGGAGTGCTTTGAGCCATCAATG
DQ859059_Zika_virus_strain_MR_766_polyprotein_gene,_complete_cds_3961	GCAGAAATGAACACTGGAGGAGACGTAGCTCACTTGGCATTAGTAGCGGCATTTAAAGTCAGACCAGCCTTGCTGGTCTCATTTATCTTCAGAGCCAACTGGACACCTCGTGAGAGCATG
DQ859059_Zika_virus_strain_MR_766_polyprotein_gene,_complete_cds_4181	TACGTGCAATGGCCGTGCCACGCACTGACAACATCGCTCTAGCAATTCTGGCTGCTCTAACACCACTAGCCCGAGGCACACTGCTCGTGGCATGGAGAGCGGGCCTCGCCACTTGTGGAG
DQ859059_Zika_virus_strain_MR_766_polyprotein_gene,_complete_cds_4291	ACTTGTGGAGGGTTCATGCTCCTCTCCCTGAAAGGGAAAGGTAGTGTGAAGAAGAACCTGCCATTCGTCGCGGCCTTGGGATTGACCGCTGTGAGAATAGTGGACCCCATTAATGTGGTG
KT365778_Zika_virus_strain_BeH819015_polyprotein_gene,_complete_cds_331	CGAGAGTTTCTGGTCATGAAAAACCCAAAAAAGAAATCCGGAGGATTCCGGATTGTCAATATGCTAAAACGCGGAGTAGCCCGTGTGAGCCCCTTTGGGGGCTTGAAGAGGCTGCCAGCC
DQ859059_Zika_virus_strain_MR_766_polyprotein_gene,_complete_cds_5611	TTGATGTGCCATGCCACCTTCACTTCACGCCTACTACAACCCATTAGAGTCCCTAATTACAACCTCTACATCATGGATGAAGCCCATTTCACAGACCCCTCAAGCATAGCTGCAAGAGGA
LC002520_Zika_virus_genomic_RNA,_complete_genome,_strain	TCCCAGCATAGCGGGATGACTGTCAATGATATAGGATATGAAACTGACGAAAATAGAGCGAAAGTCGAGGTTACGCCTAATTCACCAAGAGCGGAAGCAACCTTGGGAGGCTTTGGAAGC
KF383120_Zika_virus_strain_ArD142623_nonfunctional_polyprotein_gene,_partial_sequence_991	GCTGTCACGCTTCCTTCCCATTCCACAAGAAAGTTACAGACACGGTCACAAACCTGGCTTGAATCACGGGAGTACACGAAGCACCTGATCAAAGTTGAGAACTGGATACTCCGGAATCCA
KF383120_Zika_virus_strain_ArD142623_nonfunctional_polyprotein_gene,_partial_sequence_1101	CCGGAATCCAGGGTTCACAGTTGCTGCTCTAGCAATGGCCTGGCTCTTGGGAAATTCCACGAGCCAGCGAGTGATCTATCTGGTGATGATCCTGTTGATTGCCCCGGCATACAGTATCAG
KF383120_Zika_virus_strain_ArD142623_nonfunctional_polyprotein_gene,_partial_sequence_6821	CCTACAAAGCAGCGGCAGCCCAACTGCCGGAGACCCTAGAGACCATCATGCTGCTGGGACTGCTAGGAGCGGTGTCAATGGGAGTGTTCTTTGTATTGATGAGGAACAAAGGCATTGGGA
KF383120_Zika_virus_strain_ArD142623_nonfunctional_polyprotein_gene,_partial_sequence_6931	GGCATTGGGAAAATGGGCTTTGGAATGGTCACTCTAGGAGCTAGTGCGTGGCTCATGTGGCTCTCAGAGATCGAACCTGCTAGAATAGCCTGCGTCCTCATTGTGGTGTTTCTGTTGTTG
